# Influence of immune history when choosing a SARS-CoV-2 booster strategy

**DOI:** 10.1038/s41598-025-19659-3

**Published:** 2025-10-13

**Authors:** Soren L. Larsen, Iffat Noor, Haylee West, Eliana Chandra, Pamela P. Martinez, Alicia N. M. Kraay

**Affiliations:** 1 Program in Ecology, Evolution, and Conservation Biology, School of Integrative Biology, University of Illinois Urbana-Champaign, Urbana, IL USA; 2 Department of Demography, University of California Berkeley, Berkeley, CA USA; 3 Department of Kinesiology and Community Health, University of Illinois Urbana-Champaign, Champaign, IL USA; 4 Department of Microbiology, School of Molecular and Cellular Biology, University of Illinois Urbana-Champaign, Urbana, IL USA; 5 Department of Sociology, University of Illinois Urbana-Champaign, Champaign, IL USA; 6 Department of Statistics, University of Illinois Urbana-Champaign, Urbana, IL USA; 7 Carl R. Woese Institute for Genomic Biology, University of Illinois Urbana-Champaign, Urbana, IL USA

**Keywords:** Computational models, Viral infection

## Abstract

Given the continued emergence of SARS-CoV-2 variants of concern as well as unprecedented vaccine development, it is crucial to understand the effect of updated vaccine formulations at the population level. While bivalent formulations developed during 2022 have had higher efficacy in vaccine trials, translating these findings to real-world effectiveness is challenging due to diversity in immune history, especially in settings with a high degree of natural immunity. Known socioeconomic disparities in key metrics such as vaccine coverage, social distancing, and access to healthcare have likely shaped the development and distribution of this immune landscape. Yet little has been done to investigate the impact of booster formulation in the context of host heterogeneity. Here, we present work undertaken in 2022-2023 to inform the World Health Organization’s Immunization and Vaccines Related Implementation Research Advisory Committee (IVIR-AC), at a time when policymakers were considering optimal boosting strategies. Using two complementary mathematical models that capture host demographics and immune histories over time, we investigated the potential impacts of bivalent and monovalent boosters, inspired by disease dynamics in low- and middle-income countries (LMICs). These models allowed us to test the role of natural immunity and cross-protection in determining optimal booster strategy. Our results show that in hypothetical populations with high pre-existing immunity in the 2022-23 season, disease-related deaths from a new variant would be more sensitive to boosting/no boosting than booster formulation (bivalent vs. monovalent) - and if using bivalent formulations would result in delayed implementation compared to monovalent, it would almost always be better to implement monovalent immediately. However, deaths might be more sensitive to bivalent formulations in populations with low pre-existing immunity. These findings suggest that for many places where acquiring new vaccine stock may be economically prohibitive, monovalent boosters could still have been implemented where pre-existing immunity was high. While this analysis focuses on policy concerns in 2022, these results remain relevant now amidst ongoing questions about optimal booster formulation and timing to combat emerging transmission waves of COVID-19.

## Introduction

Host and pathogen heterogeneity are at the core of understanding infectious disease dynamics, including the potential benefit of intervention strategies like original and updated vaccine formulations. At the individual level, there is variation in behavior, viral shedding, and/or infectiousness, which can all drive transmission events across pathogens (e.g.^1,2^). At the population level, host factors can also influence disease transmission. For instance, rent-to-income ratio and population density are associated with SARS-CoV-2 superspreading in Hong Kong, a so-called ‘double disadvantage’ for impoverished individuals living in high-risk urban residential environments^3^. Lockdown mobility, testing, vaccination, and mortality during the SARS-CoV-2 pandemic have all been shown to be associated with socioeconomic status (SES)^4–6^. At the pathogen level, evolution can drive broad impacts on transmission potential. Rapid evolution observed for SARS-CoV-2 resulted in the emergence of several variants distinct from the original wild-type virus, including Omicron - sometimes with a higher rate of transmission. One early-pandemic estimate of the household secondary attack rate for SARS-CoV-2 was placed at 18.9%, but later rose to 42.7% for Omicron cases^7^. As the first COVID-19 bivalent vaccines were given emergency-use authorization in the United States in response to these new variants in August 2022^8^, we designed computational models that could address the complex web of host and population factors underlying potential booster impacts in low- and middle-income countries, particularly in the context of growing population immunity.

It is crucial to understand the interactions of these pathogen and host factors cumulatively, through time. As historical variants give way to their successors, experimental work with serological data has documented substantial differences in immune response to SARS-CoV-2 variants and vaccination by variant-specific immune history (e.g.^9–11^). This suggests the presence of an immune imprinting effect, where an individual’s prior exposure can impact the adaptive immune response to new infections^9,12^ - a phenomenon not only observed for SARS-CoV-2, but also in other respiratory viruses such as influenza and SARS-CoV-1 (e.g.^13,14^). Despite these serological findings, questions remain regarding the impacts of SARS-CoV-2 imprinting at the population level. Given that host factors are known to have influenced transmission dynamics and protective behaviors throughout the pandemic (^3–5^), they also have the potential to shape variant history and the development of the immune landscape of the population. For example, an individual with low SES who was not able to social distance early in the pandemic^4^ might be more likely to have had an early-pandemic infection prior to the emergence of new variants. On the other hand, a low SES individual is less likely to be vaccinated than a high SES counterpart^5^. Yet socioeconomic status is still a commonly under-explored axis of host heterogeneity in disease modeling^15,16^.

Mathematical models can be wielded as a powerful tool for public health when host- and pathogen-level data are plentiful, but an understanding of complex population-level dynamics is lacking^17^. Understanding booster impacts under the influence of population and pathogen heterogeneity can inform not only the acquisition and implementation of booster vaccines but also shed light on future strategies for booster formulation. In response to questions highlighted by the World Health Organization, we synthesized serological measures of variant-specific immune history, socioeconomic disparities, temporal vaccination trends, and broad variation of historical variant wave sizes from three benchmark countries (India, Ecuador, and Malaysia) to inform models of transmission for three hypothetical settings (‘High Delta Wave’, ‘High Omicron Wave’, and ‘Mixed Variant Waves’ immune landscapes respectively) and evaluated possible landscapes of immunity more broadly across low- and middle-income country (LMIC) settings. Using this immune history, we forward-simulated the potential impacts of three formulations of bivalent or monovalent boosters in two models with complementary strengths - one capable of capturing variant-specific infection history and its consequences, and the other able to simulate diverse landscapes of immunity and varying levels of pathogen immune escape or adaptation in infectiousness. While retrospective, our results have broad implications for booster vaccine development, timing, and acquisition across settings, in a viral landscape increasingly dominated by Omicron lineages^18^.

## Results

### Landscape of immunity

**Fig. 1:**
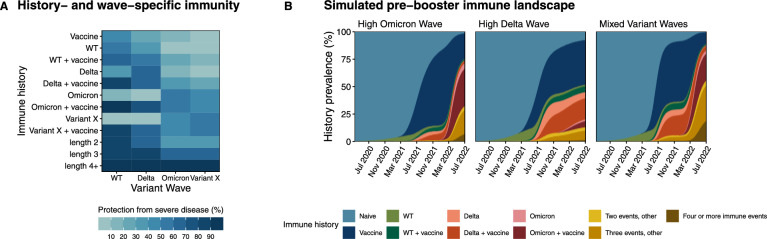
Landscapes of immunity from the history-specific model (HSM). (**A**) Level of protection from severe disease by immune history and variant wave, estimated using neutralizing antibody titers in human sera^19,20^. Immune histories of lengths 2, 3, and 4+ represent permutations of immune history that were not explicitly tracked due to insufficient data (e.g. ‘WT + Delta + Omicron’). Protection was instead treated as an average level of protection estimated for known histories of the same length. (**B**) Average immune history trajectories of 500 model runs informed by historical case incidence data from settings with diverse wave sizes shown in Supplementary Fig. S1^21^. Simulations of the prevalence of histories are shown until July 2, 2022 (day 839), one day before booster vaccinations are implemented on July 3, 2022 (28 months). The infections over time for each setting are included in Supplementary Fig. S1. General simulation parameters are shown in Supplementary Table S1, setting-specific population structure in Supplementary Table S2, contact rates by setting and SES in Supplementary Table S3, wave- and country-specific stringency in Supplementary Table S4, and vaccination parameters by setting and SES in Supplementary Table S5. The HSM compartmental diagram is shown in Supplementary Fig. S2.

In order to characterize the effect of variant-specific immunity on population-level booster impact, we implemented an individual-based model that tracks variant-specific immune history for each individual through three discrete, historical waves of SARS-CoV-2: Wild-type (WT), Delta, and Omicron. By explicitly including individual socioeconomic status - parameterized with established disparities in vaccination, contact, and infection fatality rates - the model can also capture potential asymmetry in the acquisition of immunity across groups. We refer to this as the history-specific model or HSM, and under this model, an individual’s risks of infection are explicitly influenced by their specific immune history and the currently circulating variant, while the infection fatality rate is influenced by an individual’s immune history, socioeconomic status, and age (Fig. 1A, Table S1). Using this model, we simulated three reference settings with varying historical wave sizes and vaccination trends, which we refer to as ‘High Delta Wave’, ‘High Omicron Wave’, and ‘Mixed Variant Waves’ settings.

Using the variant-specific immune histories of individuals tracked through historical simulations in the HSM, we explored the possible immune landscapes over time for ‘High Omicron Wave’, ‘High Delta Wave’, and ‘Mixed Variant Waves’ settings prior to a booster intervention (Fig. 1B) and inferred the prevalence of each history type prior to the time of boosting at 28 months (Supplementary Fig. S3). Our findings suggest that the ‘High Delta Wave’ setting had the highest share of naive individuals, with an average of 7.8% of the population having never been infected or vaccinated. Their vaccine-only population was also high (40.7%) compared to the ‘High Omicron Wave’ (23.1%) and ‘Mixed Variant Waves’ (10.8%) settings. Yet, despite having lower natural exposure overall, our simulations indicate that the ‘High Delta Wave’ setting carried by far the highest percentage of ‘Delta’ and ‘Delta + vaccine’ histories, at values of 5.5% and 19.3% respectively. In ‘Mixed Variant Waves’ simulations, ‘Delta’ exposures appeared to be less common, but there was a much higher presence of ‘Omicron’ histories than in the ‘High Delta Wave’ setting (3.0% for ‘Omicron’ and 24.4% for ‘Omicron + vaccine’). A pattern similar to that of the ‘Mixed Variant Waves’ setting was present in the ‘High Omicron Wave’ setting, where Omicron histories were also more prevalent than Delta or WT. We used these diverse immune histories, particularly in comparing vaccine-only versus hybrid immunity, to assess the population-level protection from a booster intervention under varying hypotheses of individual-level efficacy.

### Projecting the impact of booster formulation on disease incidence and deaths

**Fig. 2:**
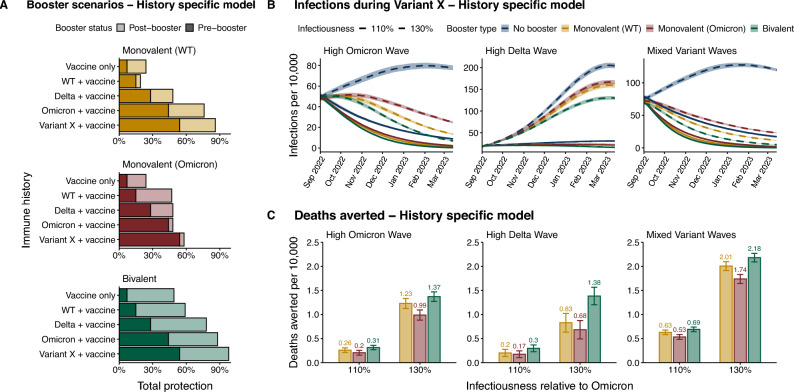
HSM booster parameters and projections during the new Variant X period. (**A**) Booster protection against severe disease during the Variant X wave, by immune history, under a bivalent, WT monovalent, or hypothetical Omicron monovalent formulation. (**B**) Mean infection trends under three boosters or a no-boosting scenario in the ‘High Omicron Wave’, ‘High Delta Wave’, and ‘Mixed Variant Waves’ settings. We simulated two scenarios of infectiousness where Variant X is 10% or 30% more infectious than Omicron. 95% confidence intervals from the t-distribution are shown (ribbons). (**C**) Deaths averted by boosting since the start of Variant X (30 months) through the end of simulations, under each booster. 95% confidence intervals from the t-distribution are shown with whiskers. Boosting parameters, which are assumed to be 10 weeks faster than for primary series vaccination, are shown in Supplementary Table S8.

Using the History-Specific Model, we introduced booster vaccines on July 3, 2022 (28 months) under three formulations - bivalent (WT + Omicron), monovalent (WT), and a hypothetical monovalent, with a single strain that was more closely matched to Omicron lineages (Omicron). Booster efficacy is stratified by prior immune history (Supplementary Fig. S4), and was parameterized based on history-specific responses to primary series vaccination. In particular, we assumed that bivalent boosters would have comparable individual level efficacy to that of primary series vaccines during the WT wave - which we estimated using neutralizing titers^19,20^ - while monovalent boosters are reduced to 40% of bivalent to have a reduced overall efficacy^22^. On September 1, 2022 (at 30 months), 2 months after the start of boosting, we introduced a hypothetical new variant which we call “Variant X”, and updated history-specific immunity for both boosted and unboosted individuals (Figs. 1A, 2A).

During this new wave, we considered two possible scenarios of changes in pathogen infectiousness, independent of immune-history conferred protection (Supplementary Table S1): one that is conservative (110% relative to Omicron) and another that more closely resembles previous increases in the observed secondary attack rate (SAR) between variants (130% relative to Omicron), considering that the SAR for Delta was $\sim 60\%$ higher than early-pandemic estimates and the SAR for Omicron was $\sim 40\%$ higher than for Delta^7^. Unsurprisingly, our results show that boosting always reduced infections (Fig. 2B) and disease-related deaths (Fig. 2C) in each of the three simulated settings during this new outbreak, regardless of formulation. For example, in the 130% infectiousness scenario, wave peaks with boosting were 19–36% lower than the peak with no boosting in the ‘High Delta Wave’ setting, 35-37% in the ‘High Omicron Wave’ setting, and 42-46% in the ‘Mixed Variant Waves’ setting. The smaller reduction observed in the ‘High Delta Wave’ simulations may be attributable to a lower boosting rate (Supplementary Fig. S5) and the distinct immune landscape compared to ‘Mixed Variant Waves’ and ‘High Omicron Wave’ settings (Fig. 1B).

In order to quantify the difference in deaths averted during the Variant X wave across booster formulations (Fig. 2C), we estimated the relative benefit of switching from monovalent (WT) to monovalent (Omicron) or to bivalent (Eq. 1) specific to each simulated setting and scenario. To quantify the relative benefit of Booster B relative to Booster A, we used the following formulation:

1 $$\begin{aligned} \text{ Relative } \text{ benefit } \text{(A,B) } = \frac{\text{ Deaths } \text{ averted } \text{ under } \text{ Booster } \text{ B }}{\text{ Deaths } \text{ averted } \text{ under } \text{ Booster } \text{ A }} \end{aligned}$$

The relative benefit is $>1$ if Booster B averts more deaths, and $<1$ if Booster A averts more deaths. Furthermore, if the relative benefit is greater than 2, the gain in deaths averted moving from Booster A to Booster B outpaces the gains moving from no boosting to Booster A. When the relative benefit is less than 2, boosting vs. no boosting has a stronger effect than booster formulation. Therefore, in cases where the relative benefit is in the interval (1, 2), then the new formulation is better than monovalent (WT) - but disease-related deaths are more sensitive to the effect of boosting than the effect of booster formulation.

When comparing the relative benefit of switching to monovalent (Omicron) from the monovalent (WT) scenario, relative benefit values were less than 1.0 in all settings (Supplementary Table S6). This poor performance may be attributable to high prevalence of recent Omicron exposure across settings (Fig. 1B). When looking at the case of bivalent boosters, we found that in the 110% infectiousness scenario, the relative benefit of bivalent boosters was 1.1, 1.19, and 1.5, for ‘Mixed Variant Waves’, ‘High Omicron Wave’, and ‘High Delta Wave’ settings, respectively. For the 130% infectiousness scenario, the relative benefit of bivalent boosters was 1.08 for ‘Mixed Variant Waves’, 1.11 for ‘High Omicron Wave’, and 1.66 for ‘High Delta Wave’ settings (Supplementary Table S6). Therefore, the relative benefit of bivalent in these scenarios was not only less than 2 - highlighting a greater sensitivity to boosting than to formulation - but also at the very bottom range of possible relative benefit in both Ecuador and Malaysia. While a low relative benefit could still lead to high absolute benefits, this was not the case for most of our scenarios. The absolute benefit was less than 0.2 deaths per 10,000 in all cases except in the ‘High Delta Wave’ setting under a 130% more infectious scenario, where 0.55 additional deaths per 10,000 could be averted by implementing a bivalent booster (Supplementary Fig. S6B).

Our finding - that switching formulations from monovalent (WT) to bivalent in a given scenario always yielded diminishing returns on the deaths averted with relative benefit values less than 2.0 - was consistent even in scenarios with conservative booster rollout speed (Figs. S5E, S7, Supplementary Table S7), in scenarios where booster curves for all countries match the superior rollout trajectory seen in Malaysia (Supplementary Fig. S8, Supplementary Table S7), and in analyses where primary series vaccination continues through the Variant X wave (Figs. S5C, S9, Supplementary Table S7). We also tested two additional assumptions on history-specific booster efficacy due to the hypothetical nature of our history-dependent booster scenarios shown in Fig. 2: (1) same efficacy, where all individuals received the same absolute change in protection from a given booster (Supplementary Fig. S10), and (2) same endpoint, where all individuals reached the same end level of protection from boosting (Supplementary Fig. S11). Bivalent boosters still broadly yielded diminishing returns (Supplementary Table S7), with the exception of the ‘High Delta Wave’ setting in the “Same Efficacy” scenario, where the relative benefit of bivalent was 2.06. We tested scenarios where monovalent booster efficacy is increased to 53% of bivalent efficacy based on work from Italy^23^, and our conclusions about the relative benefit of bivalent vs. monovalent boosting were unchanged (Supplementary Fig. S12). In the most optimistic scenario where monovalent efficacy is not reduced overall compared to bivalent, monovalent WT boosters averted more deaths per 10,000 than bivalent in the ‘High Omicron Wave’ setting (1.45 vs. 1.37) and averted equal deaths in the ‘Mixed Variant Waves’ setting (2.18) (Supplementary Fig. S13). Monovalent Omicron boosters still performed equal to or worse than WT vaccines, and were always worse than implementing a bivalent booster. In the most pessimistic scenario with uniformly poor booster efficacy compared to primary series vaccination - represented by a 50% reduction in the absolute change in protection conferred by any booster - overall deaths averted are reduced and relative benefit remains below 2.0 for bivalent boosters (Supplementary Fig. S14). Notably, in the ‘High Delta Wave’ setting, monovalent (Omicron) boosters outperformed monovalent (WT) (0.64 vs. 0.53 deaths averted per 10,000). This marginal benefit in the ‘High Delta Wave’ setting may be attributable to the higher prevalence of Delta histories and lower prevalence of Omicron histories compared to the ‘High Omicron Wave’ and ‘Mixed Variant Waves’ settings (Fig. 1B). Beyond the Variant X wave, this worst-case efficacy scenario suggests that when variant replacement progresses rapidly and results in poor strain-to-vaccine match, booster formulations based on outdated waves will perform similarly, with small differences depending on the size of preceding variant waves.

We next tested the effect of delays in booster rollout by 30, 60, or 90 days due to booster authorization and acquisition of new stock (Figs. S15–S17, Tables S13, S14). Our analysis assumed that all boosters could be implemented in July 2022, 2 months prior to the beginning of the Variant X wave, but this is an optimistic scenario. For example, bivalent boosters were announced in June 2022^24^, but not granted emergency use authorization in the United States until August 2022^8^. In these delayed scenarios, if monovalent vaccines are already approved and stocked then more deaths are averted by implementing monovalent (WT) immediately than delaying for bivalent rollout in both ‘High Omicron Wave’ and ‘Mixed Variant Waves’ settings, regardless of the length of the delay (Figs. S15–S17). In ‘High Delta Wave’ settings, a 30-day delay of bivalent is still better than implementing monovalent immediately (1.15 vs. 0.83 deaths averted per 10,000), but by 60 days, implementing monovalent immediately averts more deaths (Figs. S15–S17). While not explicitly modeled, the differences between these scenarios might be reduced if available boosters were used until new stock became available. However, the maximum potential gains that could be achieved by using a delayed bivalent booster are already minimal under our most optimistic scenarios and would remain reduced.

Finally, while neutralizing antibody titers are a strong predictor of protection from symptomatic infection^25^, and these responses are diverse across variant exposures^19,20^, in the time since this work was initially conducted T cell responses have been demonstrated to be more durable and robust to changing variants^26,27^. Therefore, in the most optimistic scenario, exposure or vaccination might induce variant-sensitive protection against infection with strong, variant-insensitive protection against severe disease. In light of this, we tested a scenario in which neutralizing antibodies dictate variable protection against infection as shown in Fig. 1A, but baseline protection against severe disease is high and uniform at 95% for all individuals with immune history, and protection after waning is reduced by 60% (history of 1 exposure), 30% (2 exposures), or 15% after (3 or more exposures). In this analysis, deaths averted are reduced overall compared to main scenarios (e.g. 0.86 vs. 2.18 deaths averted per 10,000 by a bivalent booster in the ‘Mixed Variant Waves’ setting with 130% infectiousness), but the conclusions about relative booster benefit are unchanged (Supplementary Fig. S18).

### Beyond country-specific trajectories

**Fig. 3:**
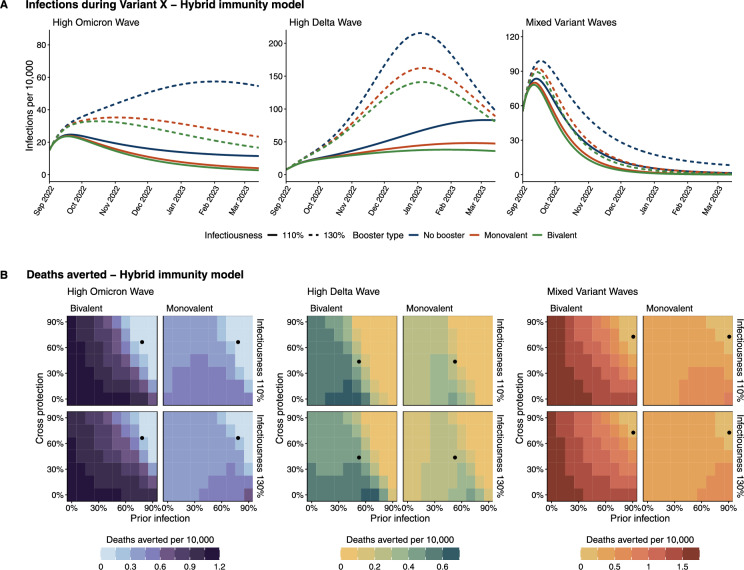
HIM projections. (**A**) Infections per 10,000 during the Variant X period, where Variant X is considered to be 10% or 30% more infectious than Omicron. (**B**) Deaths averted per 10,000 under a bivalent or monovalent booster, compared to a ‘no boosting’ scenario. Prior immunity represents the percentage of the population that has been previously infected. Cross protection represents the overlap between the population’s immune history and the currently circulating variant. Immunity levels estimated by the HSM are included for each setting (Supplementary Table S9; black dots). The HIM model structure is shown in Supplementary Fig. S2. General parameters are shown in Supplementary Table S1, stringency in Supplementary Table S4, initial conditions in Supplementary Table S10, cross-protection in Supplementary Table S9, and boosting parameters in Supplementary Table S8.

In order to explore a wider range of scenarios that capture the relative influence of pre-existing immunity and degree of overlap with the currently circulating variant in future transmission waves (which may have different immunity overlap with prior strains), we implemented an additional more flexible model, which we call the hybrid-immunity model (HIM). This model was run during the Variant X period, in addition to the HSM simulations. The HIM is a compartmental ODE model that tracks prior infection (none, one or more; not variant-specific), vaccine history (none, primary series, primary series+booster), and simulates expected cases and deaths during the Variant X wave. For the three simulated settings, the level of cross-protection, prior infection, and baseline transmission rates expected are parameterized based on the outputs of the history-specific model prior to boosting (Figs. 1B, S3, Supplementary Table S4, S9). Setting-specific booster trends are matched to the HSM (Supplementary Fig. S19), as well as prior population infection and the degree of cross protection conferred by natural infection on day 900, which are estimated using immune history distributions from day 899 in the HSM under a no-boosting scenario (Supplementary Fig. S20). These may overestimate the cross protection, but because there is limited booster impact on the number of infections during the window from day 839 to 899, this is a close approximation.

In general, projections of infections during Variant X for the three reference settings were qualitatively similar for the HIM compared with the HSM, with similar outbreaks (Fig.  3A). For the 110% infectiousness scenario, the HIM predicted small outbreaks in each of the three simulated settings. All three settings also experienced outbreaks in the 130% infectiousness scenario, with the biggest peaks in ‘Mixed Variant Waves’ and ‘High Delta Wave’ settings. Boosting reduced the size of the Variant X wave across all three settings, with bivalent boosters having a stronger impact than monovalent boosters. However, similar to the HSM, the relative and absolute impact of vaccine formulation was small (Supplementary Fig. S6C). Models revealed substantial differences across settings in the potential for booster impacts on disease-related deaths, with the ‘High Delta Wave’ setting having by far the highest number of deaths with or without boosting, matching the HSM (Figs. S21, S22). This split in deaths across places was larger overall in the HIM than in the HSM, and additionally, the HIM predicted smaller outbreaks for the ‘Mixed Variant Waves’ setting than the HSM. As a result the ‘High Delta Wave’ setting was predicted to have the strongest potential benefit of boosting on deaths averted per 10,000 (Figs. 3B (black dots), S23, Supplementary Table S11). Like the HSM, these findings from the HIM were similar in the conservative booster rollout case (Figs. S23, S24) albeit with higher overall deaths (Supplementary Fig. S21). When testing scenarios with a 60-day boosting delay (Supplementary Fig. S25), the HIM predicted similar findings to the HSM but with lower deaths averted overall (Figs. S26, S27). In all but one scenario (‘High Delta Wave’, 130% infectiousness), more deaths were averted by implementing monovalent boosters immediately than by implementing bivalent boosters with a 60-day delay, highlighting a greater sensitivity to booster timing than booster formulation.

### Consequences of immune escape and cross protection in diverse immunity contexts

**Fig. 4:**
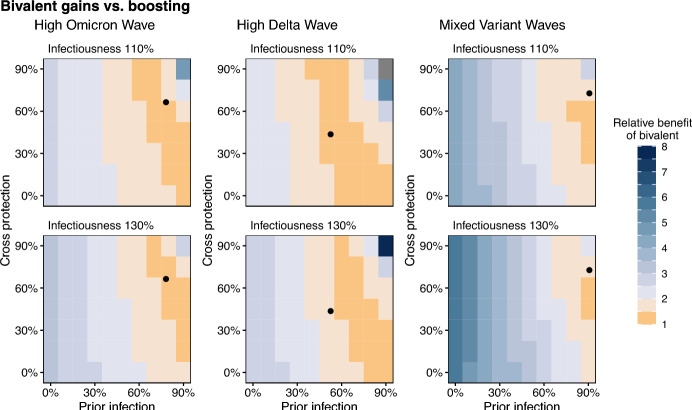
Comparison of bivalent vs. monovalent boosters in the HIM. Relative benefit of the bivalent booster is calculated with Eq. 1, matching the HSM. Grey values represent high-immunity areas where zero deaths were averted by a monovalent booster and thus Eq. 1 cannot be calculated. Prior immunity represents the percentage of the population that has been previously infected. Cross protection represents the overlap between the population’s immune history and the currently circulating variant. Immunity levels estimated by the HSM are included for each setting (Supplementary Table S9; black dots).

Broader sweeps across the immune landscape using the HIM revealed that the similarity between monovalent and bivalent booster performance was largely determined by population immunity (Figs. 3B, 4). Generally, the gains from implementing a bivalent booster were most pronounced at low levels of population immunity, but absolute differences remained relatively small for all parameter values considered (Supplementary Fig. S28). The difference between monovalent and bivalent vaccination was smallest at high levels of cross protection and prior exposure (as was seen in the three reference settings), and diverged more as the protection from natural infection decreased across both dimensions. For example, in the ‘Mixed Variant Waves’ setting, the HSM estimated that 90.7% of the population had been infected by the start of Variant X and that this protection conferred a 72.7% protection against disease-related death (Supplementary Table S9). At that pre-existing immunity level in a 130% infectiousness scenario, monovalent boosting was expected to avert 0.07 deaths per 10,000 compared with 0.11 deaths per 10,000 in the bivalent vaccination scenario, representing that the additional gain in deaths averted from jumping to an improved formulation (0.04) is just over half of what was gained when moving from no-boosting to monovalent - a relative benefit of 1.57 (Eq. 1, Fig. 4, Supplementary Table S12). These diminishing returns account for much of the high-immunity parameter space. However, in the absence of any baseline immunity, bivalent boosting could avert 1.8 deaths per 10,000 compared with 0.4 deaths per 10,000 in the monovalent case. This gain of 1.4 deaths averted per 10,000 by improving formulations, compared to 0.4 gained by simply boosting, represents a relative benefit of 4.5. In the other settings, diminishing returns accounted for a wider swath of the parameter space (Fig. 4). In ‘High Omicron Wave’ and ‘High Delta Wave’ parameter sweeps where immunity was assumed to be lower, even when the relative benefit was $>2$ the absolute difference in deaths averted per 10,000 was always less than 0.4 in ‘High Omicron Wave’ and less than 0.8 in ‘High Delta Wave’ settings (Supplementary Fig. S28). In general, at hypothetical lower levels of immunity, the ‘Mixed Variant Waves’ setting was predicted to have higher impacts than the other two settings (Fig. 3B), reflecting that booster curves in this reference setting were superior (Supplementary Fig. S19). Whereas the total deaths averted were similarly sensitive to both the cross protection and prior infections (Fig. 3B), the prevalence of prior infection was the greatest determining factor in whether a bivalent booster would be optimal.

The HSM and HIM both assumed that individuals with pre-existing immunity have strongly reduced infectiousness based on^28^, but this may not always be the case, particularly as new variants emerge. We thus tested an additional scenario in the hybrid immunity model where prior exposure or immunization had no impact on host infectiousness during the Variant X wave, under two parameterizations. First, we left all other parameters related to the force of infection unchanged, including the baseline risk of infection given contact, but removed all protection against infectiousness for groups with prior immunity during the Variant X wave so that the effective infectious proportion is equal to the total infectious proportion. In this extreme scenario, all three countries experienced large waves regardless of whether the baseline infectiousness for Variant X was 10% or 30% greater than Omicron (Supplementary Fig. S29). Under these conditions, it was almost always optimal to implement a bivalent booster (Supplementary Fig. S30). However, stringency in the HSM and HIM was calibrated to observed wave sizes for Omicron *with* the assumption of protection against infectiousness for individuals with prior immunity, so this scenario represents an extreme jump in the overall force of infection. We therefore ran an additional analysis where the overall force of infection was re-calibrated to fit the $\mathcal {R}_0$ of Omicron (Supplementary Table S1) without protection against infectiousness during the Variant X wave (Figs. S31, S32). In this scenario, cases were still increased but the relative benefit of bivalent boosting was less than 2.0 in most of the high-immunity parameter space, and in all but one wave setting (‘High Delta Wave’, 130% more infectious). In general, HIM-projected deaths tended to be lower than the HSM across sensitivity analyses (Figs. S21, S33, S34) - except in the case where transmission structure was adjusted but $\beta$ was not recalibrated (Supplementary Fig. S35) - reflecting differences in the structure of immunity. This was also reflected in the scale of the deaths averted which were generally lower across scenarios (Table 1).

**Table 1:** Comparison of HIM deaths averted per 10,000 across three scenarios: (1) Matching the HSM (column 4; as shown in Figs. 3 and 4). (2) Removing the reduction in transmission for people with pre-existing immunity (column 5), and (3) Removing the transmission reduction but recalibrating $\beta$ to remain consistent with Omicron^29^.

Setting	Infectiousness	Booster	Match HSM	Adjust transmission	Transmission recalibrated
‘High Omicron Wave’	110%	Monovalent	0.04	0.15	0.29
	110%	Bivalent	0.05	0.47	0.42
	130%	Monovalent	0.13	0.12	0.3
	130%	Bivalent	0.19	0.44	0.51
‘High Delta Wave’	110%	Monovalent	0.2	0.06	0.33
	110%	Bivalent	0.28	0.3	0.57
	130%	Monovalent	0.29	0.05	0.21
	130%	Bivalent	0.48	0.29	0.45
‘Mixed Variant Waves’	110%	Monovalent	0.03	0.16	0.23
	110%	Bivalent	0.05	0.5	0.3
	130%	Monovalent	0.07	0.14	0.28
	130%	Bivalent	0.11	0.5	0.43

Because most population immunity at the time of boosting was gained during the Omicron wave (like in ‘Mixed Variant Waves’ and ‘High Omicron Wave’ settings), we assumed that 50% of individuals with prior infection start in the recovered (R) compartment due to the short interval between transmission waves, with the rest susceptible but previously infected. This assumption could influence our estimates of booster impact, and may be less realistic for the ‘High Delta Wave’ scenario, which experienced a larger Delta wave than Omicron (Supplementary Fig. S1). We re-ran our model assuming that only 25% of individuals with prior infection start in the R compartment, and while cases and deaths were higher (Figs.S36A, S37), the overall findings were unchanged (Figs. S36, S38).

To assess how outcomes might generalize to other transmission waves (beyond assumptions on monovalent and bivalent formulations in the 2022-23 season) we performed a sensitivity analysis on booster efficacy. Here, we allowed vaccine efficacy against hospitalization to range from 50-95%, with efficacy against infection parameterized at 80% of efficacy against hospitalization. At the lower end of this range (50% efficacy against hospitalization and 40% efficacy against infection) the overall efficacy against severe disease was 70%, which was the same as the assumptions for primary series vaccination only during the 2022-23 season. In the 2022-23 season, low efficacy could reflect poor strain-to-vaccine match due to rapid immune escape of new Variant X sublineages. More generally, beyond the 2022-23 season, this lower boundary could represent a scenario where boosting provides limited benefit compared to an individual’s existing vaccine status due to continued variant replacement or antibody ceiling effects. We tested two scenarios of population immunity: one where prior infections in the population are as estimated for each setting in August 2022, and one where 90% of the population has had a prior infection, a prospect that is becoming increasingly more likely in 2023 and beyond (e.g.^30,31^). At high levels of cross-protection with the currently circulating variant, when prior infections are matched to our original estimates for each setting, we found that vaccine efficacy has a modest effect on deaths averted in both ‘High Omicron Wave’ and ‘Mixed Variant Waves’ scenarios (Supplementary Fig. S39). For example, when cross-protection is 90%, moving from 50% to 95% vaccine efficacy averts only 0.01 additional deaths per 10,000 in the ‘High Omicron Wave’, 110% infectiousness scenario. When prior infections start at 90%, then vaccine efficacy has a minimal impact in all three countries (Supplementary Fig. S40).

Finally, as in the HSM, we tested a scenario in which neutralizing antibodies dictate variable protection against infection, but peak protection against severe disease is uniformly high at 95%, reflecting the most optimistic scenario of robust and durable T cell immunity (Figs. S41, S42). Strikingly, the relative benefit of a bivalent booster is still below 2.0 in the vast majority of the parameter space, even at low levels of cross protection and base immunity. In a scenario where T cell responses are extremely robust and durable, this suggests that changing booster formulation is even less impactful.

## Discussion

The continued emergence of new variants of SARS-CoV-2 poses a challenge for maintaining effective vaccine-induced immunity in the population. As individuals continue to acquire diverse immune histories that confer highly varied protection against newer variants^9^, understanding the impact of old and new vaccine formulations in populations with varying exposure histories is crucial. For these reasons, the World Health Organization solicited models in August 2022 that could quantify booster vaccine impacts for the months ahead in the context of host heterogeneity. The models implemented here explicitly account for socioeconomic heterogeneity as a core driver of behavior and disease outcomes, and suggest that at the population level, particularly in 2022-23 where population immunity was high, disease-related deaths were more sensitive to boosting/no boosting than to booster type. Strikingly, this was largely consistent across all three simulated immune landscapes in both models, even in scenarios where rollout speed was conservative or an emerging variant was highly transmissible. Because our hybrid-immunity model simulations are not variant specific, the finding that vaccine efficacy has little impact in places with substantial natural exposure to similar variants is likely to hold not just for 2022-23 but in general. Furthermore, while bivalent benefit was higher in settings with limited immunity, this is probably an unlikely scenario for many countries. For example, a meta-analysis of serological data in November 2022 suggests seroprevalence rates at almost 100% for Africa and above 75% for some countries in Southeast Asia by the end of 2021^32^, highlighting that for many LMICs with high seroprevalence, booster formulation is likely to have a modest impact.

Limited additional benefit of bivalent boosters compared to monovalent WT boosters was exacerbated in scenarios with approval and acquisition delays, where in most cases more deaths were averted by implementing a monovalent WT booster immediately. In part, this result will depend on the level of transmission while waiting for new stock. While not explicitly tested here, continuing to use existing stock while awaiting updated formulations would be a better strategy, avoiding the excess risk occurring while awaiting the arrival of new stock, particularly if transmission rates are already high and/or increasing. This implication is at odds with initial policy recommendations by the Centers for Disease Control in the United States, which recommended that high risk individuals wait to receive their COVID-19 boosters until the new formulation became available^33^.

While bivalent boosters have since receded in favor of newer vaccine formulations, including monovalent vaccines that target the KP.2 Omicron lineage for the 2024-25 season, our findings raise questions about the tradeoff between improvements in efficacy against new variants, and speed of rollout. This is particularly true given our finding that increases in booster efficacy have only moderate impact at high levels of cross protection. Furthermore, monovalent boosters targeting the most recently circulating variant in our models did not perform better than WT monovalent formulations in populations with a high degree of natural exposure. Red Queen dynamics^34^ as well as documented imprinting effects^9^ may give rise to an endless race to capture the newest variant match. New Omicron sublineages continue to emerge, increasingly dominating the viral landscape^18,35^. If cross-protection with newer variants remains high due to overlap with original Omicron variants, then our analyses indicate that improvements in vaccine efficacy will have limited impact. In the face of this, a possible future direction for SARS-CoV-2 to make population-level gains in immunity is the development of pan-sarbecovirus or pan-coronavirus vaccines that elicit broad, cross-reactive immune responses^36,37^. Experimental work is already underway that supports this goal, but with a potentially long road ahead^36,37^.

Several limiting factors should be considered when interpreting the findings of this paper. First, our models were built in August 2022 and assumptions on the force of infection, rate of booster uptake, level of immune escape, and other factors were exploratory and hypothetical. While neutralizing antibody titers allowed us to parameterize variant-specific protection in a way that could not be done with existing clinical trial data, and correlation with protection has been previously suggested for SARS-CoV-2^38^, we acknowledge that titers may not represent protection in all cases (e.g.^39^). However, in exploratory sensitivity analyses assuming durable, variant-insensitive T cell immunity protecting against severe disease, we observed comparable tradeoffs between bivalent and monovalent boosters. In the hybrid-immunity model, booster impacts might be underestimated due to generalized booster efficacy, reduced specificity of immune history, and inclusion of age-stratified contacts. Additionally, we did not directly fit our models to data due to the difficulties of fitting individual-based models and incorporating individual immune trajectories to compartmental models. However, our findings have retrospective similarities to fall 2022 case trends for the countries that informed our reference scenarios^40^. In India for example, the peak died out faster than expected, but was very small, consistent with our 110% infectiousness scenario. Malaysia and Ecuador experienced protracted and larger waves more consistent with our predictions for the 130% infectiousness scenario, with Malaysia having more cases than Ecuador, similar to our findings when comparing the ‘Mixed Variant Waves’ setting with the ‘High Omicron Wave’ one. The resemblance of India to the 110% infectiousness scenario and Malaysia and Ecuador to 130% infectiousness scenario could be attributable to changes in contact patterns or reporting rates.

Care should be taken in interpreting the effect size of booster formulation. While we found that trying to improve booster formulation might result in diminishing returns in many cases, particularly if existing vaccine stock were not used in the interim, the absolute gain may still be sufficient to warrant implementing bivalent boosters once available. Caution should be exercised in interpreting booster impacts across countries with varying population sizes, since 1 death averted per 10,000 is a larger absolute number in a setting with 1.5 billion people compared to a setting with 20 million. When comparing the two models, the hybrid-immunity model might produce lower estimates of incidence and deaths than the history-specific model for several reasons - for example, the inclusion of age-specific contact rates (which are lower for older adults), reduced specificity of immune histories, and because immunity acquired during the current wave is likely to be stronger than pre-existing immunity, causing the cross-protection achieved by natural infection to change gradually over time during the Variant X wave for the HSM but is fixed for the HIM. Deaths averted predicted by the HIM might also be lower than the HSM because booster vaccination does not confer even short term sterilizing immunity and only reduces probability of infection and severe disease for future exposures. Beyond differences in our two models, our population-level findings are complementary to previous individual-level findings of only moderate improvements in immunity and protection against infection when boosting with bivalent formulations^24^, but other modeling work has suggested greater benefits to updated booster vaccines than estimated here^41,42^. Policymakers should exercise caution in balancing a range of estimates and metrics when deciding on optimal vaccine formulation.

In summary, incorporating numerous sources of host heterogeneity across diverse settings and alternative model structures enabled us to quantify the relative impact of boosting compared to booster formulation for the 2022-23 season. We have demonstrated a consistent finding across two models that in settings with high prior immunity, the original generation of SARS-CoV-2 monovalent boosters could have averted a similar number of deaths to the WT-Omicron bivalent boosters, without requiring the acquisition of a new vaccine stock. These results have potential implications for future vaccine policy and development. Despite differences in currently circulating variants and newly available boosters, many of our hybrid immunity model simulations are not variant or vaccine specific, adding to the generalizability of our findings. Sensitivity analysis shows that in future transmission waves, the relevance of updated boosters will depend on the level of cross protection, highlighting the benefits of mathematical models in the development of future vaccines. However, our results suggest that disease-related deaths are likely to be more sensitive to boosting rather than updated booster formulation in most scenarios, providing reassurance that in regions with limited economic resources, older vaccine stocks still have utility.

## Methods

### Selection and implementation of model settings

We chose India, Ecuador, and Malaysia as reference countries due to the diverse sizes of their observed variant waves (Supplementary Fig. S1), the availability of temporal, socioeconomically-stratified vaccine data^5^, and representation of different geographical regions. Whereas variant- and socioeconomically-specific temporal serological data is challenging to obtain, the data available for these three countries enabled us to parameterize a History-Specific Model that tracks the variant-specific immune history of individuals at each time-point, captures relative wave size for these reference locations (Supplementary Fig. S1, Supplementary Table S4), and incorporates the observed temporal vaccination trends for each setting and socioeconomic group (Supplementary Fig. S5, Supplementary Table S5). Simulations benchmarked off of India, Ecuador, and Malaysia are referred to as ‘High Delta Wave’, ‘High Omicron Wave’, and ‘Mixed Variant Waves’ settings, respectively. Simulated landscapes of immunity from the History-Specific Model (Fig. 1B) are later translated into a compartmental ODE model, referred to as the Hybrid-Immunity Model, which we use to develop a more general framework for booster impacts across settings with varying levels of immunity.

### History-specific model (HSM)

#### Immune histories

We rescaled previously reported data - neutralizing antibody titers from human sera which were stratified by immune history^19,20^ - onto a scale from 0.05-0.95, with larger numbers representing higher protection against SARS-CoV-2 (Fig. 1A). This scaling was done in order to estimate relative protection against a given variant, by variant-specific immune history. Scaling was estimated from 5-95% to avoid sterilizing or nonexistent pre-booster immunity given prior exposure. Unvaccinated titers were those from individuals with no prior vaccine doses, and vaccinated titers were those from individuals with 2 or more doses (fully vaccinated).

The base probabilities of (1) being infected after contact and (2) dying due to infections are scaled down based on these wave- and history-specific parameters. This allowed us to parameterize single-infection histories as well as hybrid histories for the WT, Delta, and Omicron waves. For individuals with 2 prior infections, which was not accounted for in this data, we took an average of the ‘strain + vaccine’ serotypes for each wave. For four or more immune history events, we assumed a protection parameter of 0.05 (95%). For three history events (three infections or two infections + vaccine), we assumed the protection would fall halfway between 2 events and 4+ events for each wave. Finally, we constructed an Variant X wave in the HSM by drifting protection down by 0.10 for all groups, equal to the change in protection moving from the WT wave to Delta. Individuals with ‘Variant X’ or ‘Variant X + vaccine’ histories are given protection during the Variant X wave equal to what ‘Omicron’ and ‘Omicron + vaccine’ have during the Omicron wave.

#### Model structure

We implemented a stochastic, individual-based transmission model in Python. This History-Specific Model spans a period of three years, through 4 discrete waves of SARS-CoV-2 variants: WT (beginning in March 2020), Delta, Omicron, and a hypothetical Omicron lineage called Variant X which we modeled to begin transmitting in September 2022. Each wave has a distinct risk of infection given contact, estimated from household secondary attack rates^7^. The simulated populations are grouped into children (0-20), adults (21-65), and elderly (older than 65) whose proportions reflect the age structure of each country^43^, with 50% being high SES and 50% low SES.

While fitting this individual-based model was not possible based on the available data, we instead implemented a stringency index with a fixed value for each wave (Supplementary Table S4) to qualitatively capture relative wave sizes for each benchmarked country that are inspired by WHO reported cases^21^. This model was then run during the Variant X wave to estimate booster impacts, keeping stringency fixed from the previous wave. We ran 500 replicates for each set of simulation parameters. Simulations progress through a modified tau-leap algorithm^44^. General parameters are shown in Supplementary Table S1. For each set of parameters in a given scenario, we calculated the 95% confidence interval across trajectories using the function smean.cl.normal() from R package HMISC^45^.

Susceptible (*S*) individuals can become exposed (*E*) through contact with an infectious individual (*I*). The probability of becoming exposed given contact is stratified by variant-specific immune history. After exposure, they become infectious. The probability that an infected individual will recover or die is stratified by age and SES^4^, as well as variant-specific immune history. While recently recovered (*R*), an individual does not have sterilizing immunity, but rather a peak level of protection dictated by their immune history (Fig. 1A), which comes from neutralizing antibody titers in human sera^19,20^, and is order-agnostic. Recently recovered individuals have a lower risk of infection and disease-related death than those in the susceptible class. Here, the protection against infection is assumed to be 80% of the values shown in (Fig. 1A) which apply to the risk of death - reflecting evidence that in the early months after SARS-CoV-2 infection or vaccination, protection against severe disease is higher than protection against any infection^46^. After recovery, individuals eventually wane into the susceptible class again, with a dampened immune history protection parameter, and the amount of this dampening is dictated by their previous number of immune exposures - with more exposure resulting in a lower degree of waning (Supplementary Table S1). The incorporation of peak (recently recovered class) and waned (susceptible class) protection as opposed to sterilizing immunity and susceptibility reflects evidence that individuals are not fully protected against reinfection even when their immune history is fresh^46^. This granularity in considering the peak and waned protection of an individual, across immune histories, is a core strength of this model.

#### Socioeconomic parameterization

Country-specific, SES-stratified contact rates in this model are estimated from pre-pandemic contact data for each country^47^ combined with estimates of unequal mobility by SES during the pandemic^4^ and the tendency towards within-SES contact vs. across-SES^48,49^ (Supplementary Table S3).

SES-stratified vaccination is implemented from day 320 (during the WT wave, around January 2021) until the beginning of Variant X, according to the true rates observed for each benchmarked country^5^ (Supplementary Fig. S5, Supplementary Table S5). Susceptible, exposed, and recently recovered individuals can be vaccinated - and if they are not already recently recovered, they move to the recently recovered class. We stopped primary series vaccination at the start of Variant X reflecting that a small number of primary series doses were projected to be given during Variant X (Supplementary Fig. S5), and to isolate the effect of boosting from interference by the primary series vaccines.

#### Boosting

Boosters function similarly to vaccination, but with faster timing and lower coverage (Supplementary Fig. S5, Supplementary Table S8). Booster rollout was assumed to be 10 weeks faster than primary series vaccination, to reflect possible improvements in infrastructure (Supplementary Fig. S5D, Supplementary Table S8). We assumed that the coverage of monovalent boosters in October 2023 represented a peak booster coverage level in the population^40^. Children and unvaccinated individuals are not eligible to receive a booster, reflecting booster eligibility at the time of the study. We note that this restrictive policy is still the case in many countries and that WHO continues to recommend that children do not need to be targeted for boosting^50^.

Data on booster efficacy by variant-specific immune history and formulation are limited. We therefore used the neutralizing titer data^19,20^ to estimate booster efficacy against severe disease. We compared the percent protection of individuals with single-variant histories during the wild-type (WT) wave (as determined by neutralizing titers) to those with [variant + vaccine] histories, and used the difference in protection to define the primary series vaccine response. We used these responses to primary series vaccination during the WT wave, stratified by variant-specific immune history, to parameterize the net gains in protection from a bivalent booster. Thus, bivalent boosters are assumed to have the same efficacy during Variant X as primary series vaccines did during the WT wave. Because Variant X is a hypothetical variant with no data on primary series vaccination responses, we assumed that the change in protection from boosting would be the same as for the Omicron variant. We considered monovalent boosters to have 40% of the efficacy of bivalent boosters against Variant X infection based on estimates of efficacy against severe infection^22^, and also tested sensitivity analyses where monovalent efficacy was 53% of bivalent^23^ or 100% of bivalent. Because we started boosting two months prior to Variant X, there was a two-month period when the bivalent and Omicron monovalent formulations were matched to the currently circulating variant, but the WT monovalent was not. To account for this, during the Omicron wave only (first two months of boosting) we increased the Omicron monovalent efficacy by 10 percentage points (the change in protection for all groups when moving from the Omicron wave to Variant X) and bivalent protection by 5 percentage points (half of what was applied for Omicron monovalent, because it contains both WT and Omicron) (Supplementary Fig. S4). After this two-month period, booster efficacy is updated on September 1, 2022, to the values shown in Fig. 2A.

### Hybrid-immunity model (HIM)

#### Model structure

We also implemented a compartmental transmission model in R, which extended a published COVID-19 transmission model^51^ to account for vaccine-specific (none, vaccinated, vaccinated + boosted), natural (0, 1+ infection), and hybrid immunity (combination). This complementary model was implemented in addition to the History-Specific Model during the Variant X period to evaluate broader sensitivity analyses. Our SEIR-like model includes six infection compartments: Susceptible *S*, Exposed *E*, Asymptomatic *A*, Symptomatic *I*, Recovered *R*, and Deceased *D* (Supplementary Fig. S2). The model was also stratified by age ($< 20$, $20-64$, and $\ge 65$ years), socioeconomic status (high vs. low), prior natural infection (no natural infection or one or more prior infection), and vaccine status (unvaccinated, primary series, and boosted). Susceptible individuals become exposed through contact with an infectious person (asymptomatic or asymptomatic), after which they enter a latent period (in the exposed *E* class). After leaving the latent class, individuals develop either symptomatic or asymptomatic infection. All asymptomatic individuals recover. Some symptomatic individuals recover, but some fraction dies. Those in the low socioeconomic group have higher mortality rates^4^, higher transmission rates because of differences in pandemic mobility^4^, and slower rates of vaccine rollout^5^ than their higher income counterparts. General parameters are shown in Supplementary Table S1.

#### Natural infection

After recovery, individuals infected for the first time have sterilizing immunity for 300 days (10 months), after which point they enter the susceptible class for individuals with prior infection ($S_2$). Our main simulations assume that 50% of those who have been infected previously have waned as of September 2022, consistent with most natural immunity having been acquired during the omicron wave, but we consider an alternative where 75% have waned as a sensitivity analysis. Individuals who have been previously infected have a lower rate of infection ($1-0.8 cp$) and mortality ($1-cp$), which is influenced by the level of cross protection (*cp*) between the prior infecting strains (based on the population-averaged immune history) and the currently circulating strain. Upon re-infection, previously infected individuals can follow the same steps as for the compartments in the base model. We only model two levels of infection, assuming that infectivity and severity are similar for secondary and higher infections. The level of pre-existing immunity and cross protection at the start of the Variant X wave was estimated for each setting based on the population immune history profiles from the history-specific model (Supplementary Table S9).

#### Vaccination

*Primary series vaccination*. Vaccination was implemented by adding additional compartments for vaccinated/vaccinated and boosted individuals, which mirror the compartments in the base model: $S_V$, $E_V$, $A_V$, $I_V$, $R_V$, and $D_V$ for vaccination. We assumed that everyone who was planning to receive their primary vaccine series had already done so as of September 2022, so only booster vaccines were included in our main model simulations, matching the HSM. Baseline vaccine prevalence in September 2022 was set in the same was as the HSM, following published data from^5^.

Vaccine efficacy for the primary series was assumed to reduce the risk of mortality by 70%, consistent with data on Astra-Zeneca protection after 6 months^52–55^. While primary series vaccination for Astra-Zeneca can reduce risk of infection by about 50%, these benefits are only present shortly after vaccination^52–55^. Given that the populations included in our model completed their vaccine campaigns earlier and current rollout rates are low, we do not model any protection against infection for the primary vaccination class.

*Booster vaccination*. To capture the potential for booster vaccination, we added additional compartments ($S_{B}$, $E_{B}$, $A_{B}$, $I_{B}$, $R_{B}$, and $D_{B}$). For simplicity, we assume that individuals in the *S*, *E*, or *R* classes can be vaccinated but individuals in the other compartments will not be. To reflect the fact that coverage of vaccine boosters was low in LMICs in September 2022, when HIM simulations began, only one boosted class was used and its efficacy was varied to reflect the potential benefit of variant specific boosters. Booster vaccination moves vaccinated individuals to the corresponding booster class, but does not confer specific temporary immunity (unlike the HSM). In other words, $S_V$ individuals move to the $S_B$ class, not the $R_B$ class. Similar to the HSM, we only model adult boosting. We implemented vaccination using dynamic daily rates using the same approach as the history-specific model (Supplementary Table S8), where daily doses were administered until coverage saturates at its peak level. We considered an optimistic roll out as our default scenario, where the halfway week of the rollout curve was sped up by 10 weeks compared to primary series vaccination, but also considered a conservative rollout as a sensitivity analysis where the speed of boosting matches primary series vaccination (Supplementary Table S5).

#### Hybrid immunity

We also explicitly accounted for joint natural immunity and vaccine-derived immunity–individuals who have both exposures are tracked in a vaccinated and infected classes ($S_{V2}$, $E_{V2}$, $A_{V2}$, $I_{V2}$, $R_{V2}$, and $D_{V2}$) or vaccinated and boosted classes ($S_{B2}$, $E_{B2}$, $A_{B2}$, $I_{B2}$, $R_{B2}$, and $D_{B2}$) and their protection from both exposures is modeled as multiplicative. For example, bivalent vaccination reduces risk of infection by 74%. In the ‘Mixed Variant Waves’ setting, cross protection from prior infection was estimated at 72.8%. Thus, an individual with both exposures has an infection risk of $(1-0.74)\times (1-0.73) \times \beta$ or a combined reduction in infection risk of 93%. We do not differentiate between the order of vaccination and natural infection in the hybrid immunity model.

Booster vaccination can enhance protection against both infection ($VE_i$) and severe disease ($VE_h$). We use scenarios consistent with the individual-based model bivalent booster efficacy ($VE_i=0.74$, $VE_h=0.925$) and monovalent booster efficacy ($VE_i=0.657$, $VE_h=0.5256$) in the main analysis. While this yields a lower efficacy against severe disease specifically for the monovalent booster ($VE_h$) compared with primary series vaccination, the total protection against severe disease is higher (84%) due to the inclusion of protection against infection.

## Supplementary Information

Supplementary Information.

## Data Availability

Neutralizing antibody titers data were obtained from^19,20^. SARS-CoV-2 household secondary attack rates were obtained from^7^. Population estimates and projections come from census data^44^. Reported cases were downloaded from the WHO portal^21^. Country-specific contact matrices were obtained from^48^. Estimates of relative mobility by SES where obtained from^4^. Within-SES contact vs. across-SES were inferred using data from^49,50^. SES-stratified vaccination rates were obtained from^5^. Booster coverage was downloaded from Our World Data website^41^. Monovalent booster efficacy values with respect to the bivalent booster were obtained from^22,23^. Vaccine efficacy values used to parameterize the hybrid-immunity model were obtained from^53–55^. Code and data generated and analyzed during the current study are publically available at the following Zenodo repository: 10.5281/zenodo.17122487.
