# Supplementary material for: Influence of immune history when choosing a SARS-CoV-2 booster strategy
Source: Sci Rep. 2025 Oct 13;15:35640. doi: 10.1038/s41598-025-19659-3 (PMC12518628; doi:10.1038/s41598-025-19659-3)
Supplement: Supplementary file 1 — Supplementary Information. [file 41598_2025_19659_MOESM1_ESM.pdf]

Supplementary material for: Influence of immune history when choosing a SARS-CoV-2 booster strategy

Soren L. Larsen[1,2], Iffat Noor[3], Haylee West[4], Eliana Chandra[5,6], Pamela P. Martinez[4,6,7*], and Alicia N. M. Kraay[3,7*]

[1]*Program in Ecology, Evolution, and Conservation Biology, School of Integrative Biology, University of Illinois Urbana-Champaign, Urbana, IL, USA*
[2]*Department of Demography, University of California Berkeley, Berkeley, CA, USA*
[3]*Department of Kinesiology and Community Health, University of Illinois Urbana-Champaign, Champaign, IL, USA*
[4]*Department of Microbiology, School of Molecular and Cellular Biology, University of Illinois Urbana-Champaign, Urbana, IL, USA*
[5]*Department of Sociology, University of Illinois Urbana-Champaign, Champaign, IL, USA*
[6]*Department of Statistics, University of Illinois Urbana-Champaign, Urbana, IL, USA*
[7]*Carl R. Woese Institute for Genomic Biology, University of Illinois Urbana-Champaign, Urbana, IL, USA*

S1 Supplementary Text

Population structure

In the HSM, we divided the population of each setting into age groups of 0-20, 21-65, and 66+ relative to their true country-specific distributions [1]. These population distributions were scaled to have a total size of approximately 100,000 individuals (Supplementary Table S2). For the HIM, actual population sizes were used in the simulations with age groups 0-19, 20-64, and 65+. For both models, 50% of each population was assigned to be high and low socioeconomic status (SES) respectively.

History specific model

Variant-wave transitions

Beginning with Wild Type (WT) SARS-CoV-2 at day 0 of the pandemic, we then simulated the Delta wave (beginning on day 420), Omicron wave (day 630) and a new hypothetical variant Variant X (day 900) which lasts until the end of the simulations on day 1095 (year 3). Due to the computational intensity of a four-variant, history-specific model as well as limitations in data availability for multi-variant histories, we made a simplifying assumption that each variant wave is discrete (no co-circulation of variants). At the end of a previous wave, when a new wave begins any remaining infected individuals are converted to the new variant. In addition, at the beginning of each wave 25 exposed individuals per SES class are seeded into the model to ensure that immediate die-out does not occur.

Force of infection

Each of the first three waves has a unique baseline probability of infection given contact, denoted by β. This value is also scaled by the agent's prior immune history at 80% of the protection for severe disease that are shown in Figure 1A. To capture relative differences in contact across countries, we started with pre-pandemic estimates of contact for children, adults, and elderly [2] and took weighted averages to get total contact across countries. Because we modeled historical waves, we required wave-specific estimates of the probability of infection given contact, and these were taken from household secondary attack rates [3]. Consequently, to account that our probabilities of infection were taken from close contact, we scaled down total contacts by 75% to get baseline contact values for the model. Because mobility tends to occur more within SES-groups rather than outside of it [4, 5], we assumed 60% of contacts are with individuals from the same SES group and 40% are from the opposite group.

Next, we applied pandemic-related reductions in contact. Social distancing is known to be unequal across SES-groups [6], and we accounted for this relative disparity by giving low SES a 30% social distancing reduction and high SES a 60% reduction within each setting (Supplementary Table S3). Finally, each benchmarked country has a varied history of NPIs including masking or stay-at-home orders. To capture the relative sizes of each wave, reflected in historical confirmed cases for each country (Supplementary Fig. S1), we parameterized a stringency index from 0-1 that applies to both socioeconomic groups within a given setting, that reduces the effective contact for each wave (Supplementary Table S4). During the transition from Omicron to Variant X, stringency is held constant, but we consider the probability of becoming infected to be 10% or 30% higher, representing an increase in infectiousness of the pathogen.

Death or recovery

The probability of death following infection, denoted by α, is stratified by age and socioeconomic status using previous estimates by our group [6], and scaled by immune history (Figure 1A). If an individual recovers instead, the variant they were infected with is appended to their immune history. We assume that peak immunity, whether natural or vaccine-induced, lasts 10 months.

Waned immunity

Ten months after an immune event, immunity wanes to a lower level and agents move to the susceptible class. The degree to which their protection from infection and severe disease is dampened depends on how many immune events n they have previously had, with more prior events n reducing the amount of waning – if $n = 1$, protection wanes to 40% of initial peak protection, if $n = 2$ then 70%, and if $n \geq 3$ then 85%.

Vaccines and boosting

Using country- and SES-specific vaccination rates over time previously published by our group [7], we incorporated an initial vaccine intervention during the WT, Delta, and Omicron waves, considering the shape of initial rollout (k), eventual maximum covered (V_m) and week at which half of the population is vaccinated (W_h) (Supplementary Table S5). The vaccine intervention starts at day 320, representing that vaccination started several months before Delta became the prevailing strain, and continues until the end of the Omicron wave. Anyone who is not currently infectious and has not previously received a vaccine is eligible to be vaccinated, and vaccination moves the agent to the recovered class, with vaccination appended to their immune history. At each time step, the model computes the proportion of the total population that should be vaccinated that day, and attempts to draw that number of individuals from the vaccine-eligible pool. If the eligible pool is smaller than the prescribed number to be vaccinated, the model vaccinates all individuals in the eligible pool.

Starting on day 840 (two months before Variant X) and continuing until the end of the simulations, we implemented a booster intervention. Maximum potential booster uptake (B_m)for each setting was based on uptake for the previous campaign of monovalent boosters [8], with the assumption that only vaccinated adults are eligible. To get these coverages, we first calculated the proportion of the total vaccinated individuals that belong to each SES class, based on the maximum vaccinated values for each country (Supplementary Table S5). We then calculated the number of these doses which went to adults, using the population structure of each country (Supplementary Table S2). Assuming that boosters would continue to have the same distribution across low and high SES as for the primary series vaccines, we calculated the maximum number of booster doses given to each SES class, then divided these values over the number of vaccinated adults to parameterize the peak coverage among vaccinated adults (Supplementary Table S8). We used the same shape (kB) as the primary series vaccination. We considered two speeds, where timing (WB_h) was the same (supplementary analysis) or 10 weeks faster (main text) than primary series vaccination (Supplementary Fig. S5).

Booster protection scenarios

The precise responses to booster vaccines stratified by strain-specific immune history, are presently unknown. For this reason, we considered several hypotheses of booster efficacy during Variant X, using proportional changes deduced from the known immune response to monovalent primary series vaccines during

the WT wave. We considered bivalent and WT monovalent boosters, as well as a hypothetical Omicron monovalent booster. Previous work suggests that monovalent boosters may have 40% of the efficacy of bivalent boosters against severe infection [9]. Informed by this estimate, we assumed that a bivalent formulation would have the same absolute changein protection that was originally conferred by primary series vaccination during the WT wave, while monovalent boosters confer 40% of this original absolute change.

Scenario 1: History Dependent

For WT monovalent boosters, each individual's absolute change in protection is 40% of their absolute change in protection during primary series vaccination against WT. We also constructed hypothetical Omicron monovalent boosters, where the response of 'WT + vaccine' and 'Omicron(*) + vaccine' are flipped. In the bivalent case, WT and Omicron(*) histories receive a response equal to the average of the WT and Omicron(*) responses during the WT wave, because the booster contains both WT and Omicron antigens (Figure 2A). There is no data on primary series vaccination for individuals with an Variant X history since this is a hypothetical scenario, and thus their responses cannot be individually calculated. Consequently, for the purposes of vaccination and boosting, we assume that individuals who have been exposed to the new Variant X variant respond the same to each booster as those with Omicron histories.

Scenario 2: Same Efficacy

In this scenario, individuals receive an absolute change in protection equal (bivalent) or 40% less (monovalent) to the average observed change across 'WT + vaccine', 'Delta + vaccine', 'Omicron + vaccine', and 'Vaccine' histories during primary series vaccination against WT (Supplementary Fig. S10A).

Scenario 3: Same Endpoint

In this scenario, we move all individuals to the same end protection parameter, equal to the maximum protection observed in the same efficacy scenario (Supplementary Fig. S11A).

Scenario 4: No boosting

Finally, we considered a scenario in which no booster is implemented.

Calculating events

We used a modified tau-leap algorithm to determine which events (infection, recovery, etc.) would occur and when [10]. Vaccination and boosting proceeds separately from the tau-leap, and is performed at time intervals of 1 day.

Hybrid immunity model

Model structure

A model diagram is shown in Fig. S2A. The flow of individuals through the model was as follows. Initially, many individuals are susceptible to infection (S). Upon exposure, they enter a latent period (E), during which they cannot transmit. They can then develop asymptomatic infection (entering the A class) or symptomatic infection (entering the I class). We assume that all asymptomatic individuals will recover (R). Those with

symptomatic infections can either recover (entering the R class) or die (entering the deceased class D), and the rest will recover (entering the R class).

Immunity

For country-specific benchmarking for the HIM, we calculated the weighted average of cross protection among existing prior infections prior to boosting (Supplementary Table S9). This was calculated by combining the wave specific cross protection for each natural infection history against Variant X and multiplying by their relative abundance among the previously infected population.

Force of infection

As a starting point, we used contact matrices estimated for each country pre-pandemic, stratified by age [2]. The probability of infection given contact among all contacts was calibrated to match the force of infection in the HSM (Supplementary Table S1). We also tested two scenarios where infectiousness is not reduced by pre-existing immunity: one where the probability of infection is unchanged, and another where the probabilities of infection were recalibrated at 4.79% for low SES and 1.82% for high SES to still match the infectiousness of Omicron [11]. While the HIM included assortatitivity in contacts by age, it did not include assortativity by SES, though overall contact was lower for high SES matching the HSM. We also used results from the HSM calibration to parameterize the baseline stringency for each site so that our force of infection values were relatively comparable.

Booster coverage, efficacy, and rollout rates

Since the HIM starts at the start of the Variant X wave and the main simulations assumed that boosting started 60 days before Variant X, we calculated the starting booster coverages using the type-III functional response functions with the expected rollout curve from days 0 to 60 and using day 60 as initial coverage on day 1 of the HIM simulations. Rollout rates were parameterized to match the HSM, including the peak booster coverages adjusted to exclude children and unvaccinated from boosting (Supplementary Table S8). Because immune history was not explicitly modeled and was captured using baseline immunity and cross protection due to natural infection, the HIM roughly corresponds to the same endpoint or same efficacy scenarios from the individual-based model (Figures S11, S10).

Initial conditions

The initial age distribution of infections and deaths were estimated using seroprevalence data and incidence data from Our World In Data (OWID) [8]. To initialize the model, we calculated a baseline reporting rate by comparing seroprevalence data reported from the individual countries with reported cases from Our World In Data as of 12 days before the estimated seroprevalence study in each country was conducted to allow time for seroconversion [12–14]. The ratio between reported cases 12 days prior to the completion of each seroprevalence study and the population incidence corresponding to the seroprevalence reported in each study was the direct estimate of the reporting rate. The estimated reporting rates for both symptomatic and asymptomatic infections were 0.178 for Malaysia, 0.053 for India, and 0.189 for Ecuador. We assumed that only symptomatic infections were reported. Reported cases as of October 12, 2022 were multiplied by 1/reporting rate to get the total number of active symptomatic infections on that date. We assumed that only 40% of infections were symptomatic, so asymptomatic infections were assumed to be 1.5 times the total active symptomatic conditions. Initial latent infections were set equal to I(0)+E(0).

For our main simulations, we set baseline immunity for the country-benchmarked simulations to its estimated value from the HSM. Then, to explore how future dynamics for any country depend on prior infection, we varied the level of baseline immunity from 0-100%. For each level of immunity, we assumed that the prevalence of immunity was the same by vaccine strata and 50% of previously infected cases were assumed to have waned immunity into the higher S class. For example, for a scenario with 50% baseline immunity, 25% of the unvaccinated population was put into the R class and 25% of this class was put into S_2. No individuals were assumed to be in the R_2 classes at the start of the simulation, equivalent to assuming that no individuals had experienced more than 1 prior infection. We also relaxed this assumption in sensitivity analyses, and the results were similar.

S2 Figures

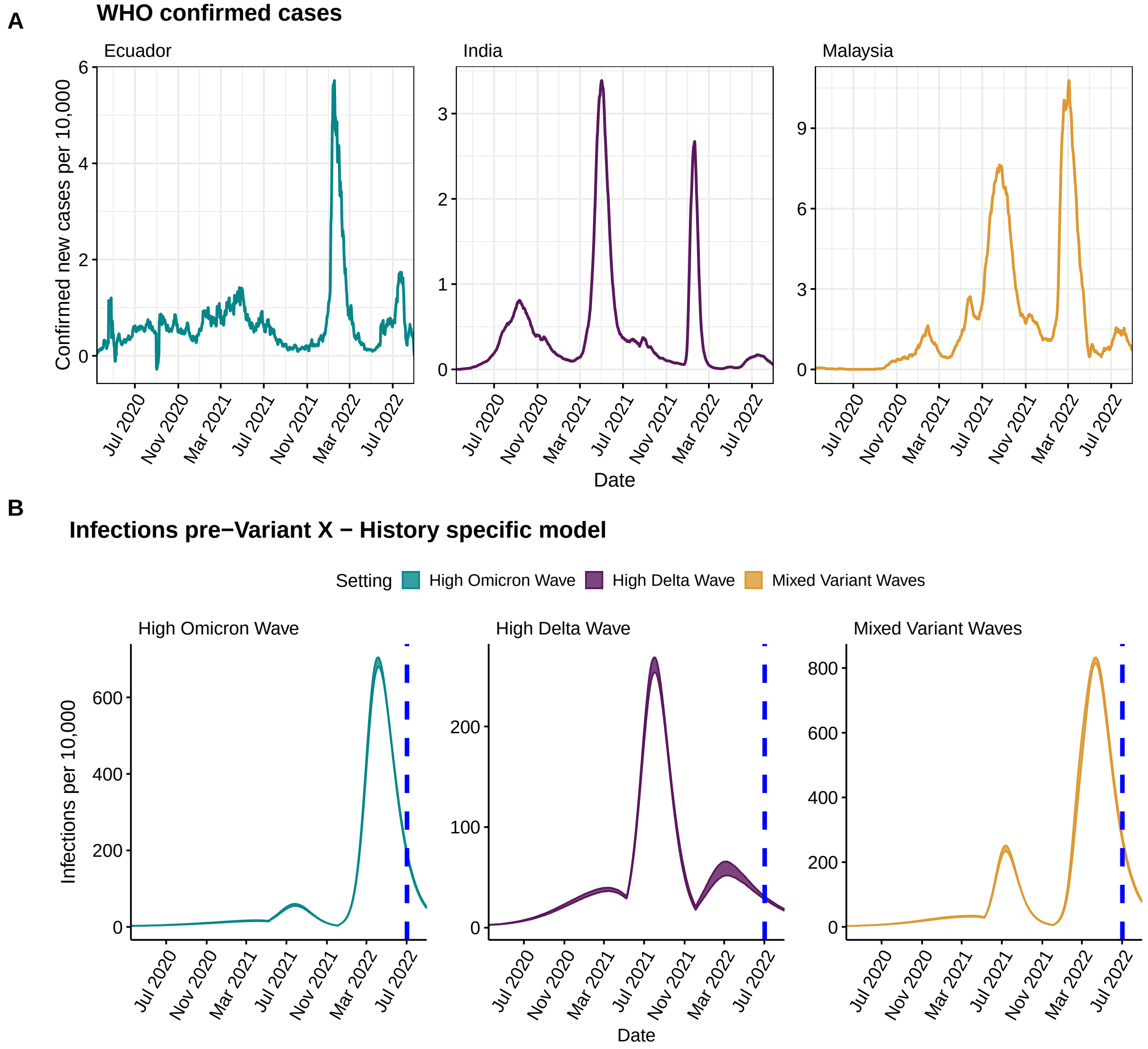

Figure S1: Infections before Variant X. (A) Moving 7-day average of confirmed cases per 10,000 population prior to the Variant X wave. Data from WHO [15]. (B) HSM-simulated infections for 'High Delta Wave', 'High Omicron Wave', and 'Mixed Variant Waves' before Variant X in the "No booster" scenario, with 500 replicates in each setting. 95% confidence intervals from the t-distribution are shown (ribbons). Dashed lines denote the start of booster vaccination.

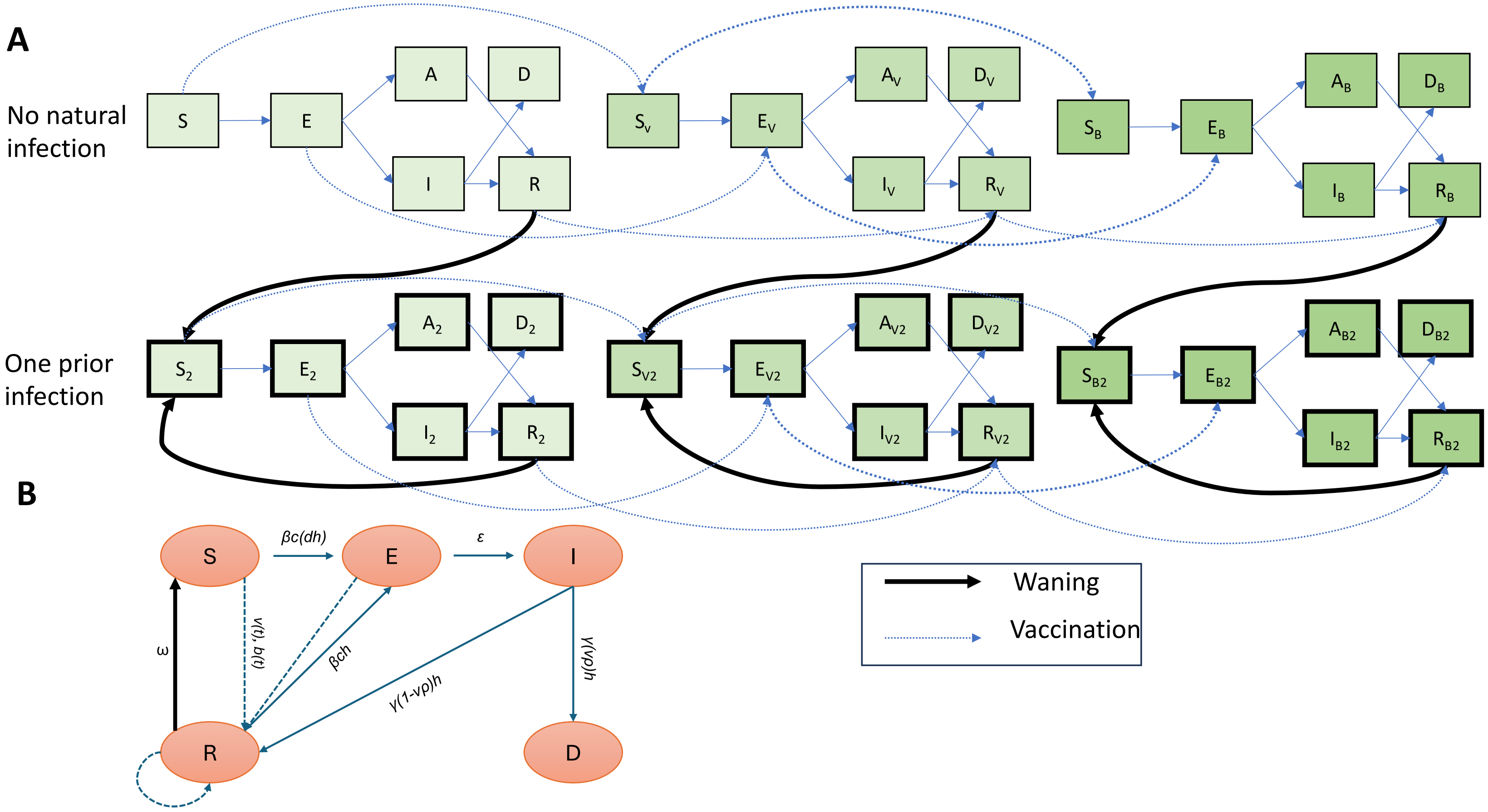

Figure S2: Compartmental model diagram for the hybrid immunity model (A) and history specific model (B). In panel A, box colors indicate vaccine status (lightest green: unvaccinated, medium green: primary series vaccination, dark green: boosted); V and B subscripts denote vaccinated and boosted status, respectively; a subscript of 2 indicates at least one prior infection. For both models, the dark black line indicates waning immunity and the dotted line shows vaccination events. In panel (B), parameter h represents the immune history scaling parameter for infections (when multiplied by β) or deaths (when multiplied by ρ). In both models, actively infectious individuals are not eligible for vaccination, but become eligible upon recovery.

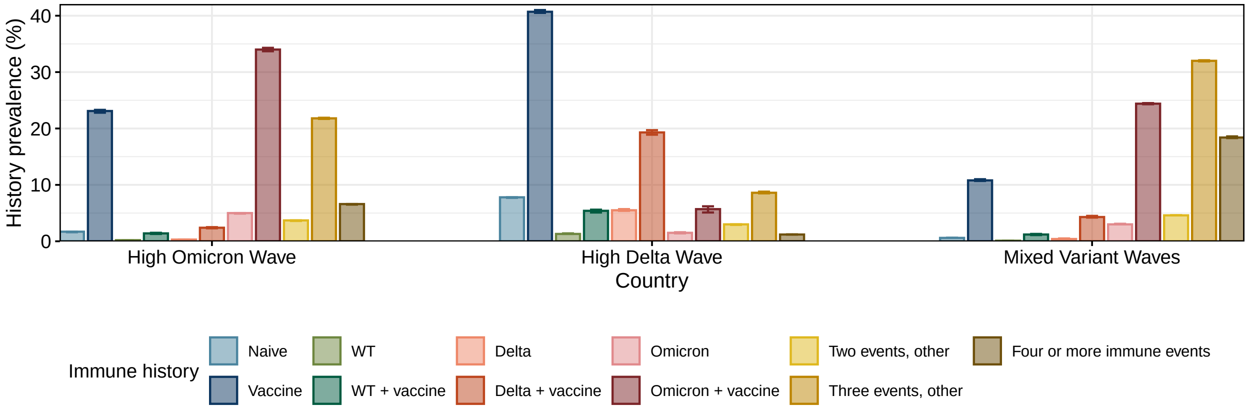

Figure S3: Distribution of immune histories by setting in the HSM on day 839 with 500 replicates, as seen in Figure 1A, prior to the beginning of booster vaccination. 95% confidence intervals from t-distribution are shown (whiskers).

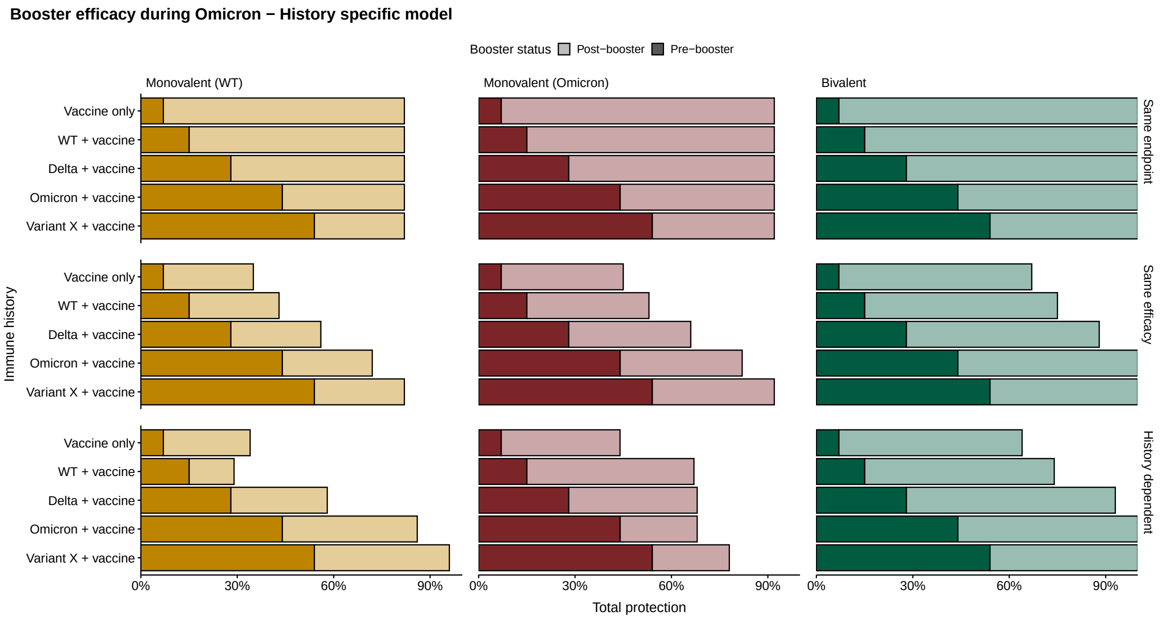

Figure S4: Booster protection against severe disease during the Omicron wave by immune history, under a bivalent, WT monovalent, or hypothetical Omicron monovalent formulation. These values are used for 60 days, from the start of boosting at day 840 until the start of the Variant X wave on day 900.

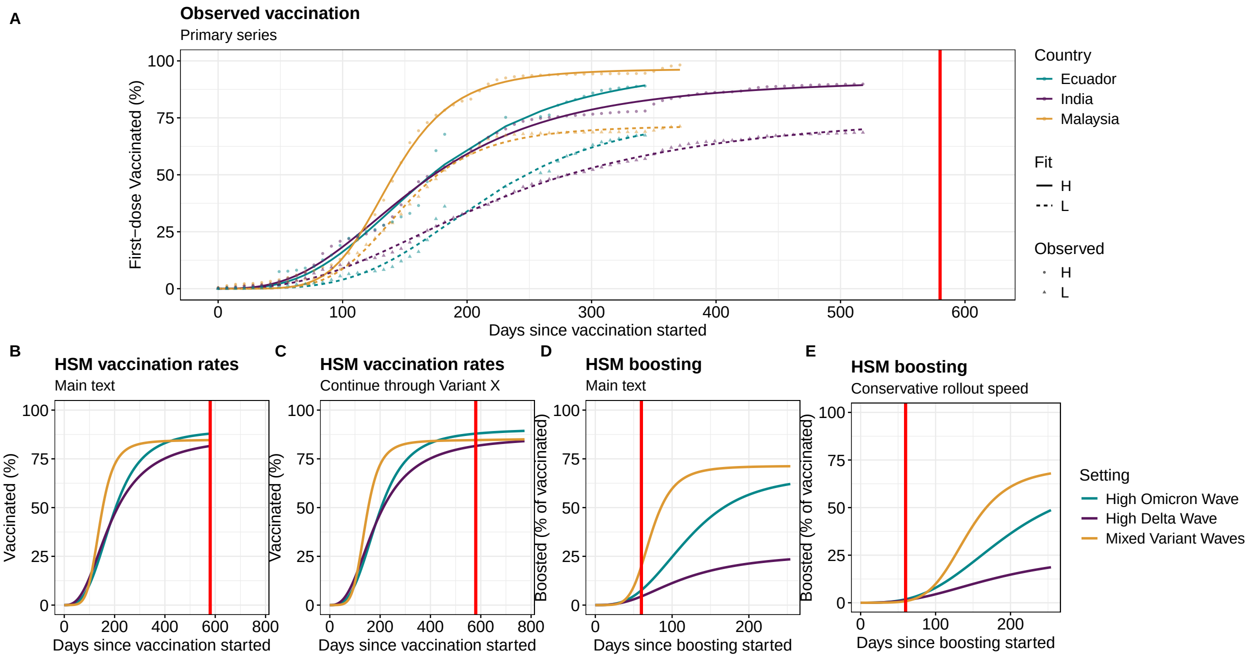

Figure S5: HSM vaccine and booster trends. (A) Observed and fit data of primary series vaccination for Ecuador, India, and Malaysia [7]. (B) Vaccination rates in the HSM, if vaccination stops at the end of the Omicron wave. (C) Vaccination rates in the HSM, if vaccination continues through Variant X. (D) Booster rates in the HSM, if booster timing is 10 weeks faster than primary series vaccination for all groups. (E) Booster rates in the HSM, if booster timing is the same as primary series vaccination. Red lines denote the start of Variant X.

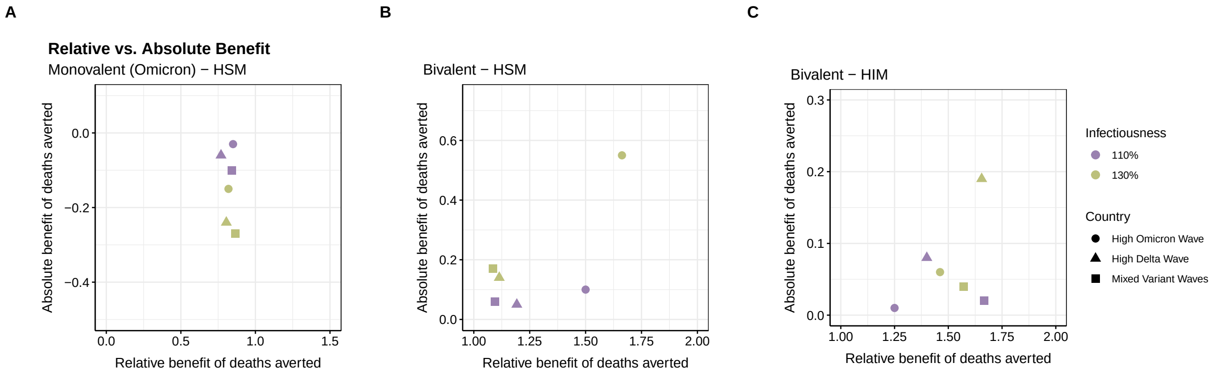

Figure S6: Relative versus absolute benefit of monovalent (Omicron) and bivalent boosters during the Variant X wave on the deaths averted per 10,000. Country-benchmarked estimates are shown for the HSM (A,B) and HIM (C).

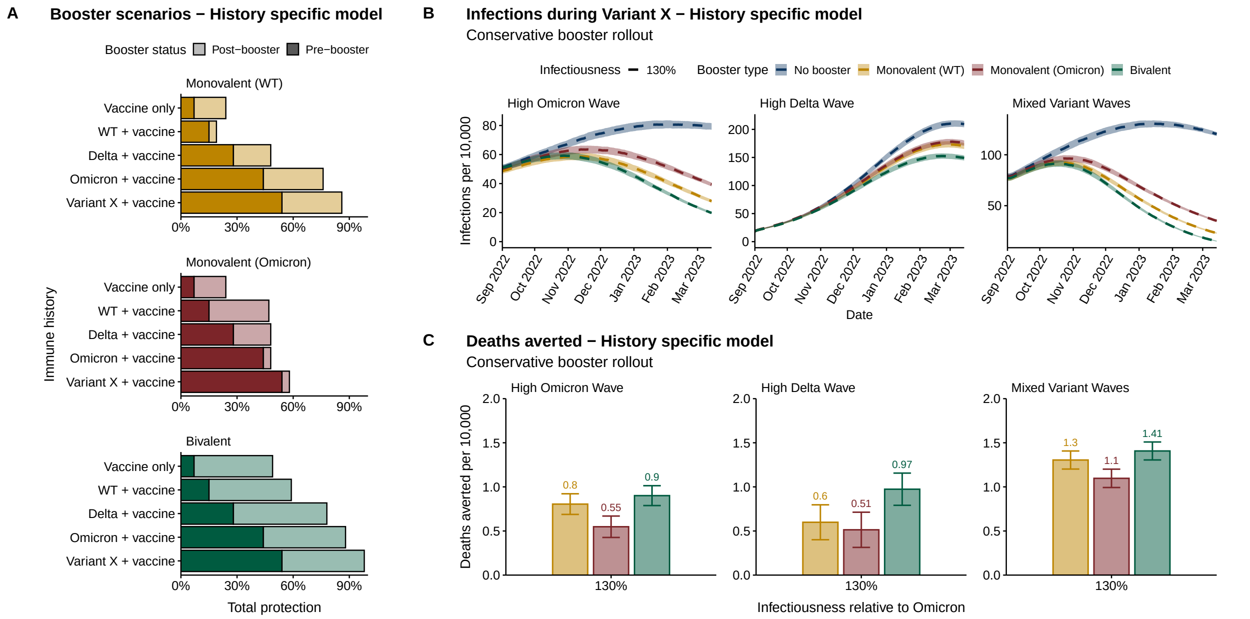

Figure S7: HSM booster impacts under the assumption that the timing of booster vaccination is the same as primary series rollout. (A) Booster protection against severe disease during the Variant X wave, by immune history, under a bivalent, WT monovalent, or hypothetical Omicron monovalent formulation. (B) Mean infection trends with 500 replicates under three boosters or a no-boosting scenario for 'High Omicron Wave', 'High Delta Wave', and 'Mixed Variant Waves' settings if Variant X is 30% more infectious than Omicron. 95% confidence intervals from the t-distribution are shown (ribbons). (C) Deaths averted by boosting since the start of Variant X (30 months) through the end of simulations (36.5 months), with 500 replicates under each booster. 95% confidence intervals from the t-distribution are shown with whiskers. The HSM model structure is shown in Supplementary Fig. S2. General simulation parameters are shown in Supplementary Table S1, population structure in Supplementary Table S2, contact rates by country and SES in Supplementary Table S3, wave- and country-specific stringency in Supplementary Table S4, and vaccination parameters by country and SES in Supplementary Table S5. The maximum boosted and shape of boosting parameters are shown in Supplementary Table S8, and the timing of boosting matches primary series vaccination (Supplementary Table S5).

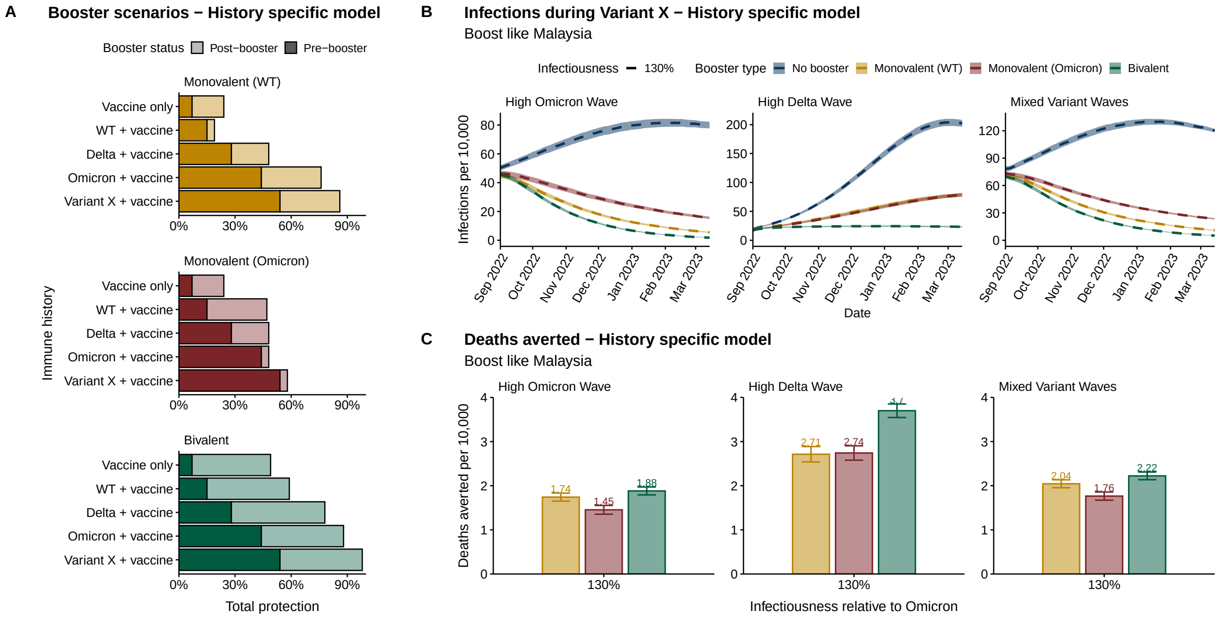

Figure S8: HSM booster impacts under the assumption that all countries boost with the same booster trend as Malaysia. (A) Booster protection against severe disease during the Variant X wave, by immune history, under a bivalent, WT monovalent, or hypothetical Omicron monovalent formulation. (B) Mean infection trends with 500 replicates under three boosters or a no-boosting scenario for 'High Omicron Wave', 'High Delta Wave', and 'Mixed Variant Waves' settings if Variant X is 30% more infectious than Omicron. 95% confidence intervals from the t-distribution are shown (ribbons). (C) Deaths averted by boosting since the start of Variant X (30 months) through the end of simulations (36.5 months), with 500 replicates under each booster. 95% confidence intervals from the t-distribution are shown with whiskers. The HSM model structure is shown in Supplementary Fig. S2. General simulation parameters are shown in Supplementary Table S1, population structure in Supplementary Table S2, contact rates by country and SES in Supplementary Table S3, wave- and country-specific stringency in Supplementary Table S4, and vaccination parameters by country and SES in Supplementary Table S5. Boosting parameters for Malaysia are shown in Supplementary Table S8.

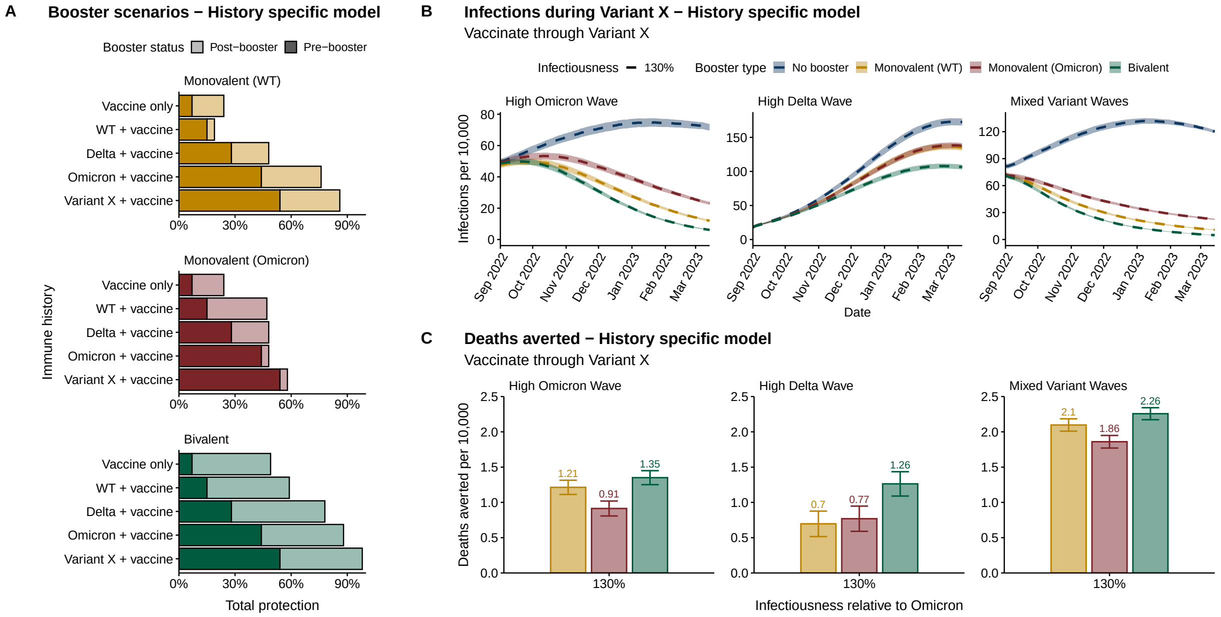

Figure S9: HSM booster impacts under the assumption that vaccination continues through Variant X. (A) Booster protection against severe disease during the Variant X wave, by immune history, under a bivalent, WT monovalent, or hypothetical Omicron monovalent formulation. (B) Mean infection trends with 500 replicates under three boosters or a no-boosting scenario for 'High Omicron Wave', 'High Delta Wave', and 'Mixed Variant Waves' settings if Variant X is 30% more infectious than Omicron. 95% confidence intervals from the t-distribution are shown (ribbons). (C) Deaths averted by boosting since the start of Variant X (30 months) through the end of simulations (36.5 months), with 500 replicates under each booster. 95% confidence intervals from the t-distribution are shown with whiskers. The HSM model structure is shown in Supplementary Fig. S2. General simulation parameters are shown in Supplementary Table S1, population structure in Supplementary Table S2, contact rates by country and SES in Supplementary Table S3, wave- and country-specific stringency in Supplementary Table S4, and vaccination parameters by country and SES in Supplementary Table S5. Boosting parameters are shown in Supplementary Table S8.

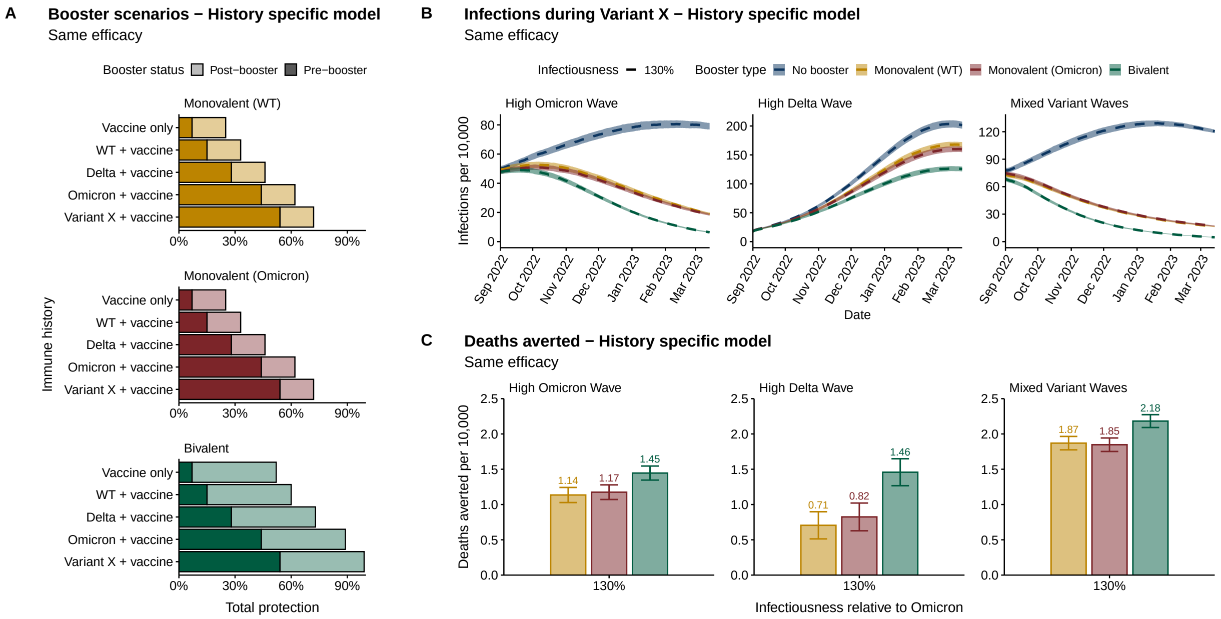

Figure S10: HSM booster impacts under the assumption that boosting results in the same absolute change in protection for all individuals ("Same efficacy"). (A) Booster protection against severe disease during the Variant X wave, by immune history, under a bivalent, WT monovalent, or hypothetical Omicron monovalent formulation. (B) Mean infection trends with 500 replicates under three boosters or a no-boosting scenario for 'High Omicron Wave', 'High Delta Wave', and 'Mixed Variant Waves' settings if Variant X is 30% more infectious than Omicron. 95% confidence intervals from the t-distribution are shown (ribbons). (C) Deaths averted by boosting since the start of Variant X (30 months) through the end of simulations (36.5 months), with 500 replicates under each booster. 95% confidence intervals from the t-distribution are shown with whiskers. The HSM model structure is shown in Supplementary Fig. S2. General simulation parameters are shown in Supplementary Table S1, population structure in Supplementary Table S2, contact rates by country and SES in Supplementary Table S3, wave- and country-specific stringency in Supplementary Table S4, and vaccination parameters by country and SES in Supplementary Table S5. Boosting parameters, which are assumed to be 10 weeks faster than for primary series vaccination, are shown in Supplementary Table S8.

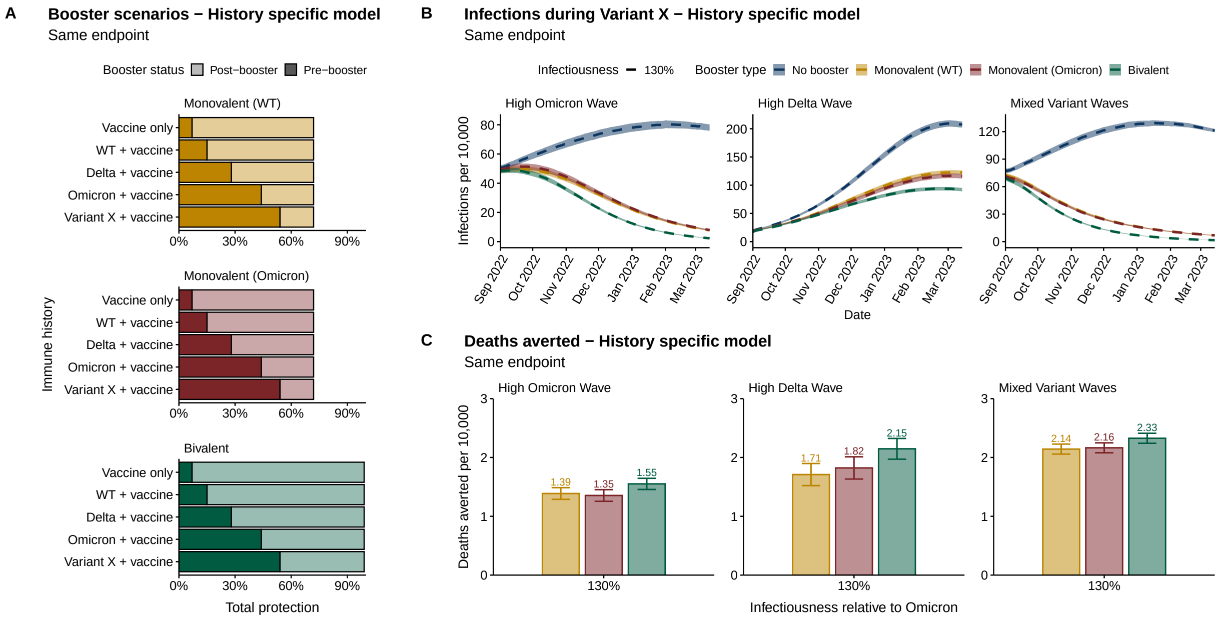

Figure S11: HSM booster impacts under the assumption that boosting results in the same end protection for all individuals ("Same endpoint"). (A) Booster protection against severe disease during the Variant X wave, by immune history, under a bivalent, WT monovalent, or hypothetical Omicron monovalent formulation. (B) Mean infection trends with 500 replicates under three boosters or a no-boosting scenario for 'High Omicron Wave', 'High Delta Wave', and 'Mixed Variant Waves' settings if Variant X is 30% more infectious than Omicron. 95% confidence intervals from the t-distribution are shown (ribbons). (C) Deaths averted by boosting since the start of Variant X (30 months) through the end of simulations (36.5 months), with 500 replicates under each booster. 95% confidence intervals from the t-distribution are shown with whiskers. The HSM model structure is shown in Supplementary Fig. S2. General simulation parameters are shown in Supplementary Table S1, population structure in Supplementary Table S2, contact rates by country and SES in Supplementary Table S3, wave- and country-specific stringency in Supplementary Table S4, and vaccination parameters by country and SES in Supplementary Table S5. Boosting parameters, which are assumed to be 10 weeks faster than for primary series vaccination, are shown in Supplementary Table S8.

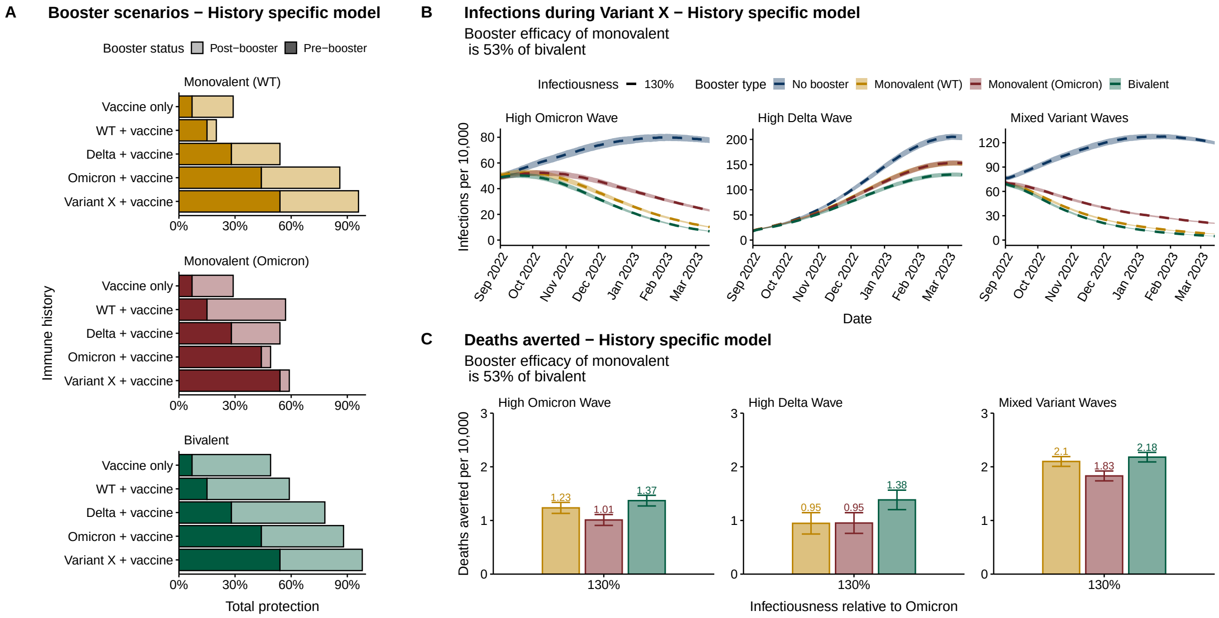

Figure S12: HSM booster impacts under the assumption that monovalent booster efficacy is reduced to 53% of that of bivalent [16]. For both "Bivalent" and "No booster" scenarios, main text replicates from Figure 2 are shown. (A) Booster protection against severe disease during the Variant X wave, by immune history, under a bivalent, WT monovalent, or hypothetical Omicron monovalent formulation. (B) Mean infection trends with 500 replicates under three boosters or a no-boosting scenario for 'High Omicron Wave', 'High Delta Wave', and 'Mixed Variant Waves' settings if Variant X is 30% more infectious than Omicron. 95% confidence intervals from the t-distribution are shown (ribbons). (C) Deaths averted by boosting since the start of Variant X (30 months) through the end of simulations (36.5 months), with 500 replicates under each booster. 95% confidence intervals from the t-distribution are shown with whiskers. The HSM model structure is shown in Supplementary Fig. S2. General simulation parameters are shown in Supplementary Table S1, population structure in Supplementary Table S2, contact rates by country and SES in Supplementary Table S3, wave- and country-specific stringency in Supplementary Table S4, and vaccination parameters by country and SES in Supplementary Table S5. Boosting parameters, which are assumed to be 10 weeks faster than for primary series vaccination, are shown in Supplementary Table S8. For both (B) and (C), "No booster" and "Bivalent" replicates from the main text scenarios are shown.

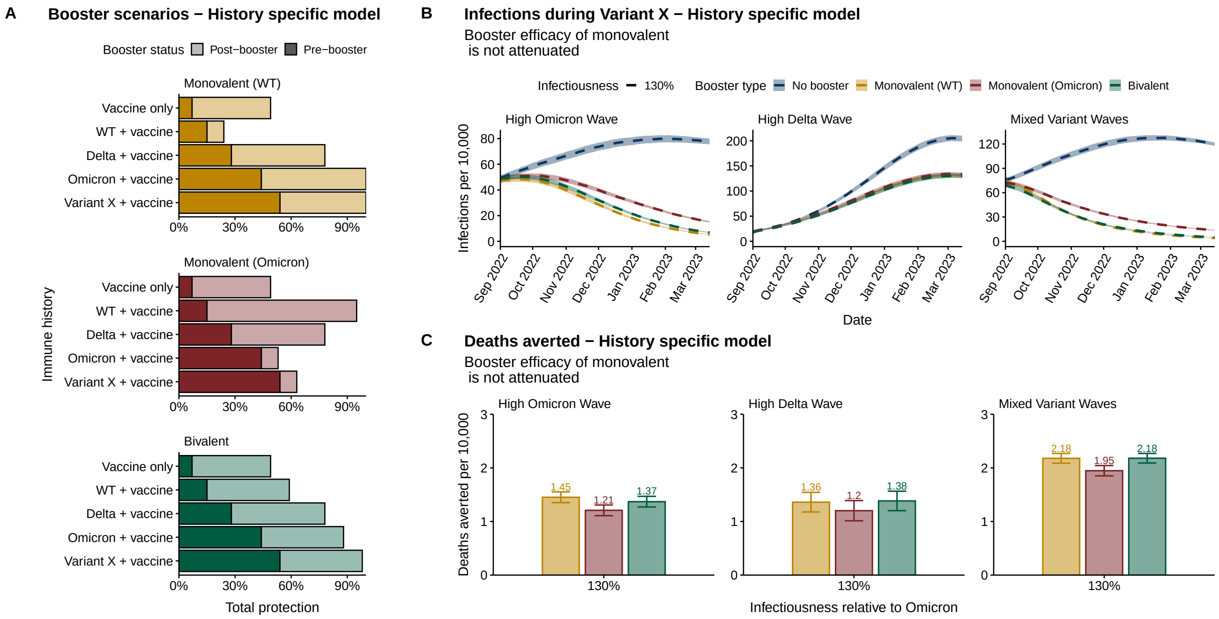

Figure S13: HSM booster impacts under the assumption that monovalent booster efficacy is *not* reduced compared to bivalent. For both "Bivalent" and "No booster" scenarios, main text replicates from Figure 2 are shown. (A) Booster protection against severe disease during the Variant X wave, by immune history, under a bivalent, WT monovalent, or hypothetical Omicron monovalent formulation. (B) Mean infection trends with 500 replicates under three boosters or a no-boosting scenario for 'High Omicron Wave', 'High Delta Wave', and 'Mixed Variant Waves' settings if Variant X is 30% more infectious than Omicron. 95% confidence intervals from the t-distribution are shown (ribbons). (C) Deaths averted by boosting since the start of Variant X (30 months) through the end of simulations (36.5 months), with 500 replicates under each booster. 95% confidence intervals from the t-distribution are shown with whiskers. The HSM model structure is shown in Supplementary Fig. S2. General simulation parameters are shown in Supplementary Table S1, population structure in Supplementary Table S2, contact rates by country and SES in Supplementary Table S3, wave- and country-specific stringency in Supplementary Table S4, and vaccination parameters by country and SES in Supplementary Table S5. Boosting parameters, which are assumed to be 10 weeks faster than for primary series vaccination, are shown in Supplementary Table S8.

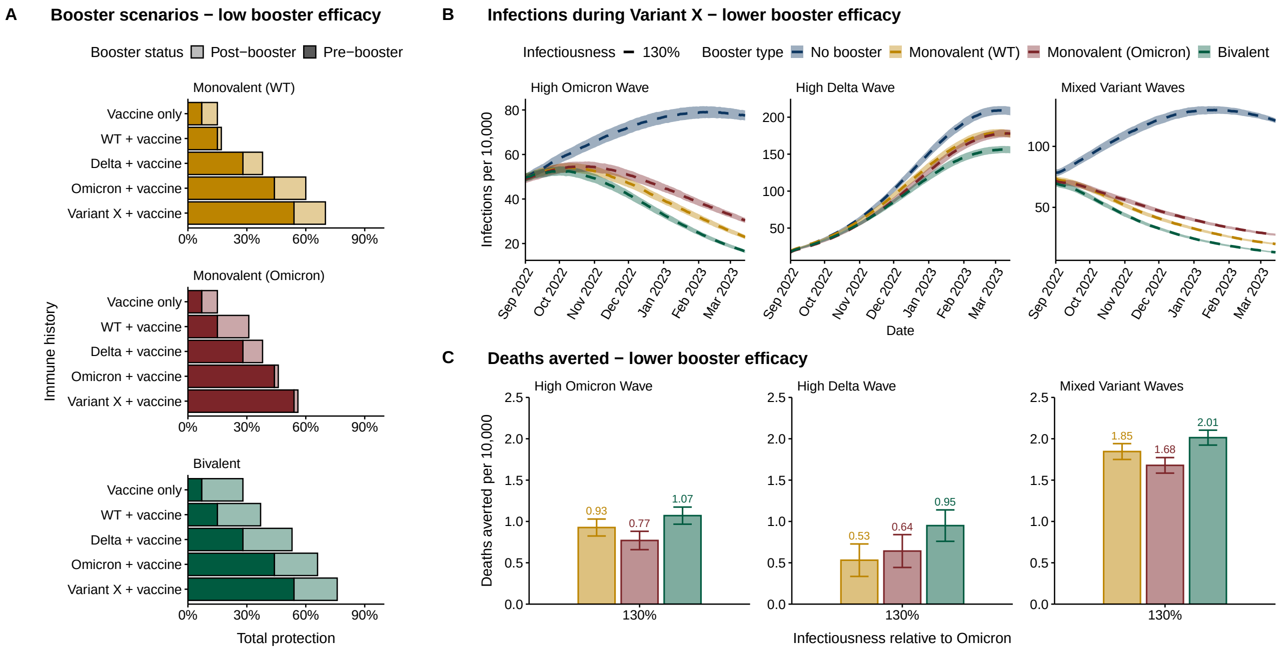

Figure S14: HSM booster impacts under the assumption that the current round of boosters has uniformly poor efficacy compared to primary series vaccination, represented by a 50% reduction in the absolute change in protection conferred by any booster. (A) Mean infection trends with 500 replicates under three boosters or a no-boosting scenario for 'High Omicron Wave', 'High Delta Wave', and 'Mixed Variant Waves' settings if Variant X is 30% more infectious than Omicron. 95% confidence intervals from the t-distribution are shown (ribbons). (B) Deaths averted by boosting since the start of Variant X (30 months) through the end of simulations (36.5 months), with 500 replicates under each booster. 95% confidence intervals from the t-distribution are shown with whiskers. The HSM model structure is shown in Supplementary Fig. S2. General simulation parameters are shown in Supplementary Table S1, population structure in Supplementary Table S2, contact rates by country and SES in Supplementary Table S3, wave- and country-specific stringency in Supplementary Table S4, and vaccination parameters by country and SES in Supplementary Table S5. Boosting parameters, which are assumed to be 10 weeks faster than for primary series vaccination, are shown in Supplementary Table S8.

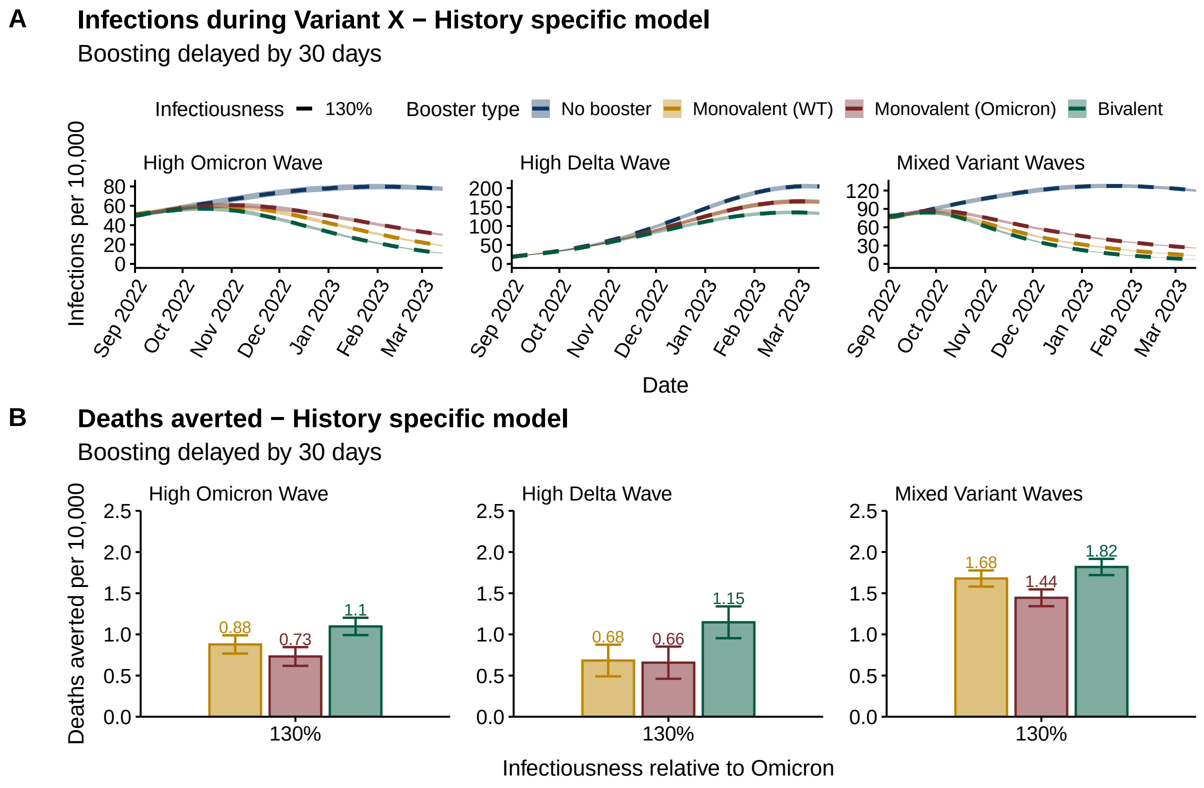

Figure S15: HSM booster impacts under the assumption that all boosting is 30 days delayed, regardless of formulation. For "No booster" scenarios, main text replicates from Figure 2 are shown. (A) Mean infection trends with 500 replicates under three boosters or a no-boosting scenario for 'High Omicron Wave', 'High Delta Wave', and 'Mixed Variant Waves' settings if Variant X is 30% more infectious than Omicron. 95% confidence intervals from the t-distribution are shown (ribbons). (B) Deaths averted by boosting since the start of Variant X (30 months) through the end of simulations (36.5 months), with 500 replicates under each booster. 95% confidence intervals from the t-distribution are shown with whiskers. The HSM model structure is shown in Supplementary Fig. S2. General simulation parameters are shown in Supplementary Table S1, population structure in Supplementary Table S2, contact rates by country and SES in Supplementary Table S3, wave- and country-specific stringency in Supplementary Table S4, and vaccination parameters by country and SES in Supplementary Table S5. Boosting parameters, which are assumed to be 10 weeks faster than for primary series vaccination, are shown in Supplementary Table S8.

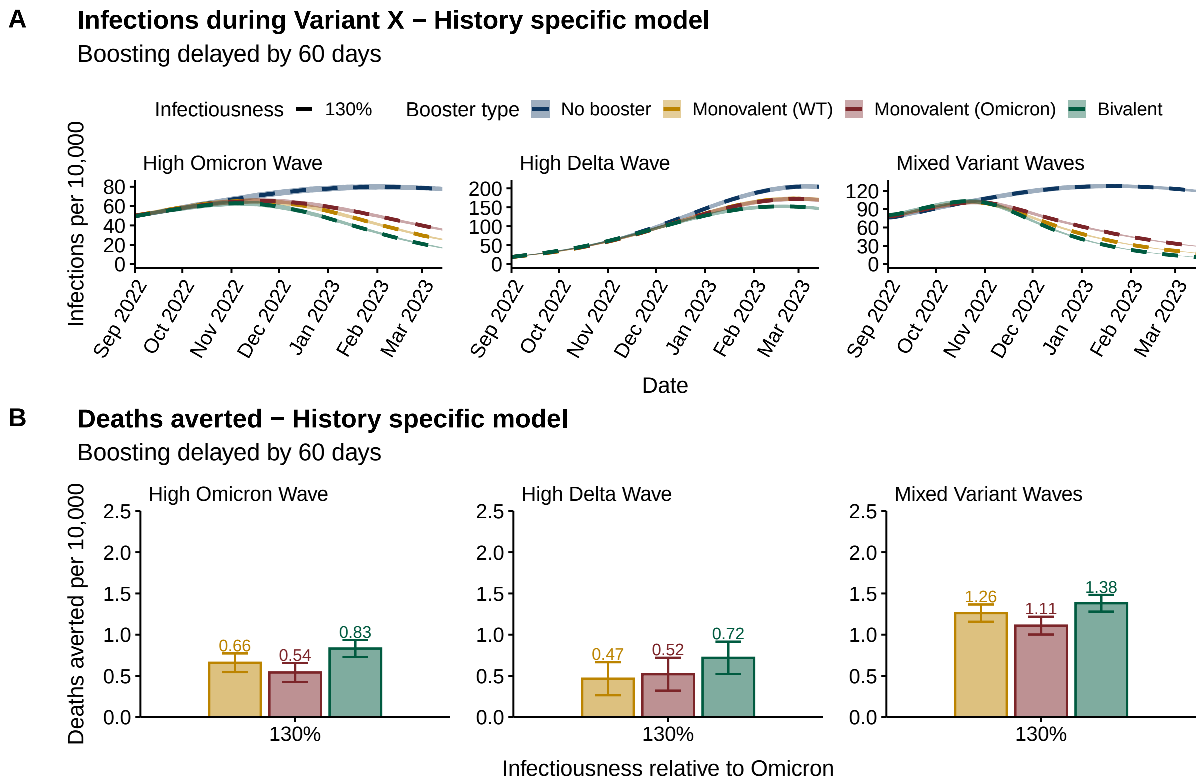

Figure S16: HSM booster impacts under the assumption that all boosting is 60 days delayed, regardless of formulation. For "No booster" scenarios, main text replicates from Figure 2 are shown. (A) Mean infection trends with 500 replicates under three boosters or a no-boosting scenario for 'High Omicron Wave', 'High Delta Wave', and 'Mixed Variant Waves' settings if Variant X is 30% more infectious than Omicron. 95% confidence intervals from the t-distribution are shown (ribbons). (B) Deaths averted by boosting since the start of Variant X (30 months) through the end of simulations (36.5 months), with 500 replicates under each booster. 95% confidence intervals from the t-distribution are shown with whiskers. The HSM model structure is shown in Supplementary Fig. S2. General simulation parameters are shown in Supplementary Table S1, population structure in Supplementary Table S2, contact rates by country and SES in Supplementary Table S3, wave- and country-specific stringency in Supplementary Table S4, and vaccination parameters by country and SES in Supplementary Table S5. Boosting parameters, which are assumed to be 10 weeks faster than for primary series vaccination, are shown in Supplementary Table S8.

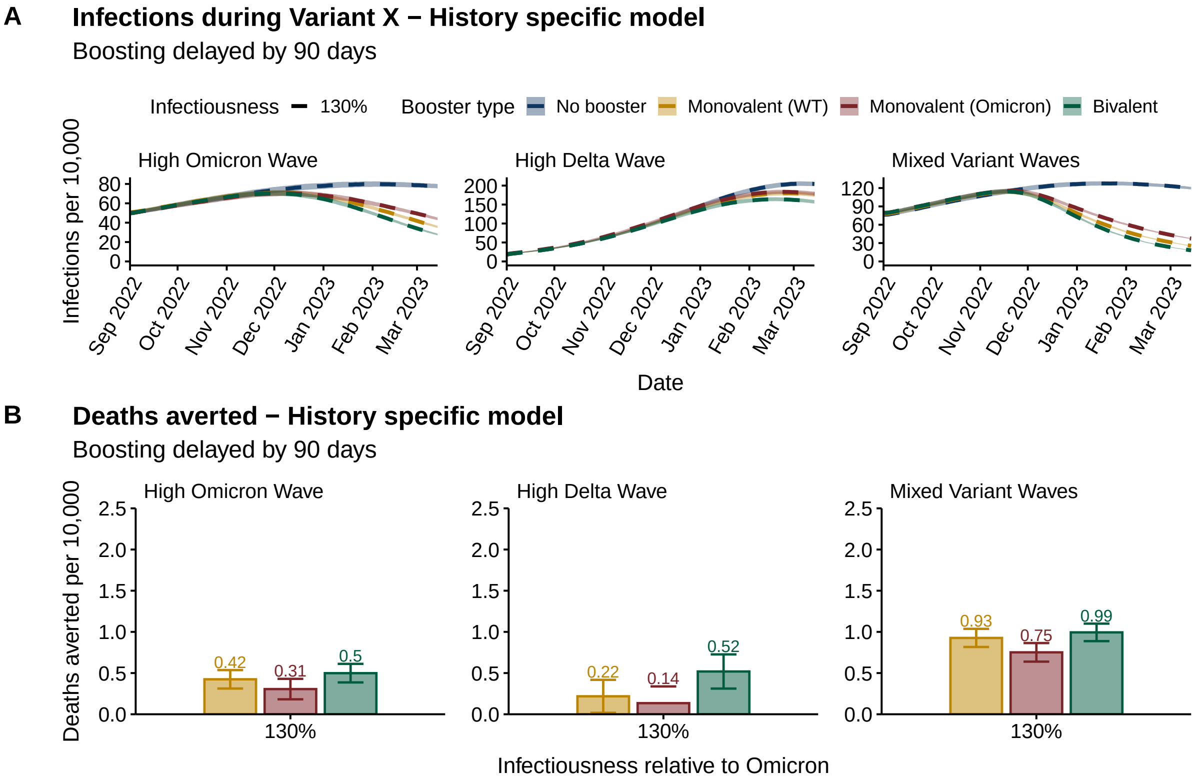

Figure S17: HSM booster impacts under the assumption that all boosting is 90 days delayed, regardless of formulation. For "No booster" scenarios, main text replicates from Figure 2 are shown. (A) Mean infection trends with 500 replicates under three boosters or a no-boosting scenario for 'High Omicron Wave', 'High Delta Wave', and 'Mixed Variant Waves' settings if Variant X is 30% more infectious than Omicron. 95% confidence intervals from the t-distribution are shown (ribbons). (B) Deaths averted by boosting since the start of Variant X (30 months) through the end of simulations (36.5 months), with 500 replicates under each booster. 95% confidence intervals from the t-distribution are shown with whiskers. The HSM model structure is shown in Supplementary Fig. S2. General simulation parameters are shown in Supplementary Table S1, population structure in Supplementary Table S2, contact rates by country and SES in Supplementary Table S3, wave- and country-specific stringency in Supplementary Table S4, and vaccination parameters by country and SES in Supplementary Table S5. Boosting parameters, which are assumed to be 10 weeks faster than for primary series vaccination, are shown in Supplementary Table S8.

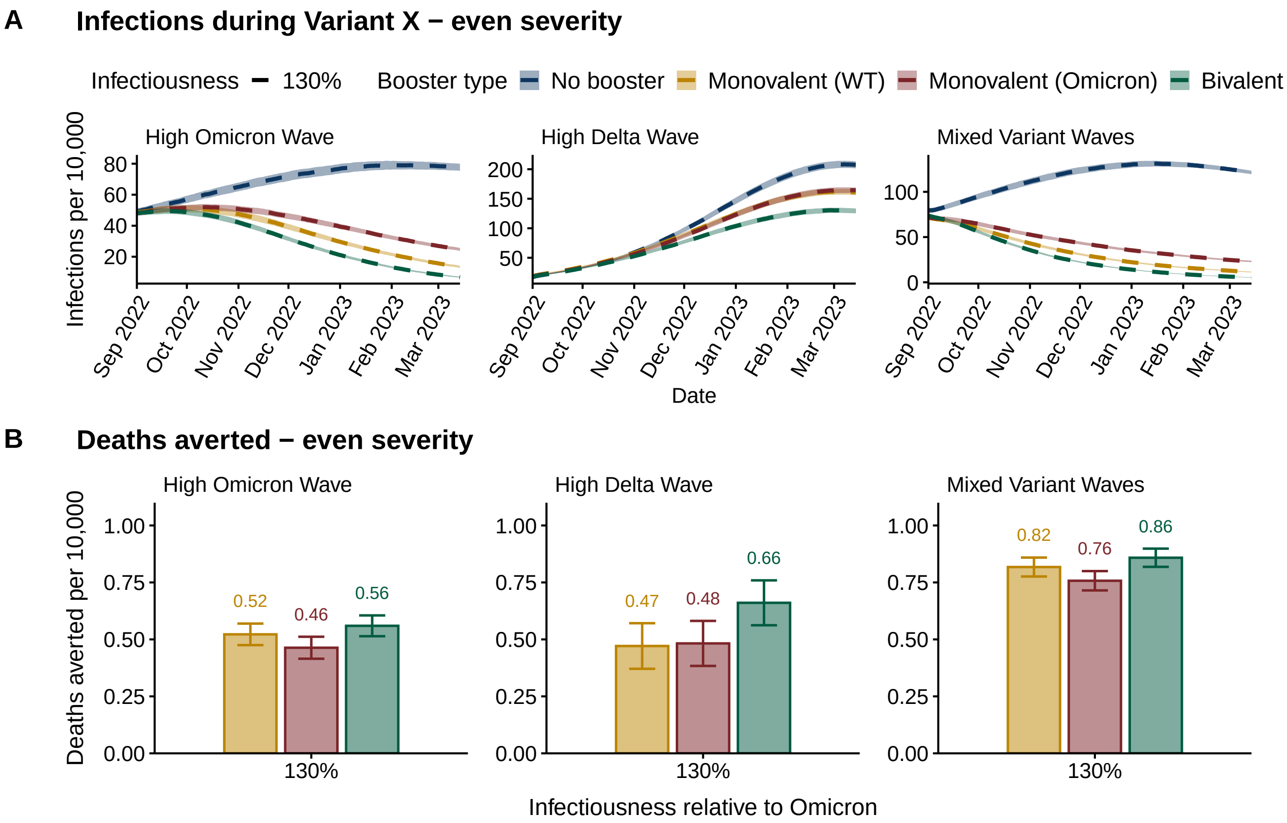

Figure S18: HSM booster impacts under the assumption that protection against severe disease is uniformly 95% after a recent exposure or vaccination, and wanes at the same rate for all individuals. This represents a scenario where protection against infection is driven by neutralizing antibody responses, which differ in quality across immune histories and are sensitive to immune escape, but protection against severe disease is driven by durable T and B cell responses that are less sensitive to the changing variant landscape. (A) Mean infection trends with 500 replicates under three boosters or a no-boosting scenario for 'High Omicron Wave', 'High Delta Wave', and 'Mixed Variant Waves' settings if Variant X is 30% more infectious than Omicron. 95% confidence intervals from the t-distribution are shown (ribbons). (B) Deaths averted by boosting since the start of Variant X (30 months) through the end of simulations (36.5 months), with 500 replicates under each booster. 95% confidence intervals from the t-distribution are shown with whiskers. The HSM model structure is shown in Supplementary Fig. S2. General simulation parameters are shown in Supplementary Table S1, population structure in Supplementary Table S2, contact rates by country and SES in Supplementary Table S3, wave- and country-specific stringency in Supplementary Table S4, and vaccination parameters by country and SES in Supplementary Table S5. Boosting parameters, which are assumed to be 10 weeks faster than for primary series vaccination, are shown in Supplementary Table S8.

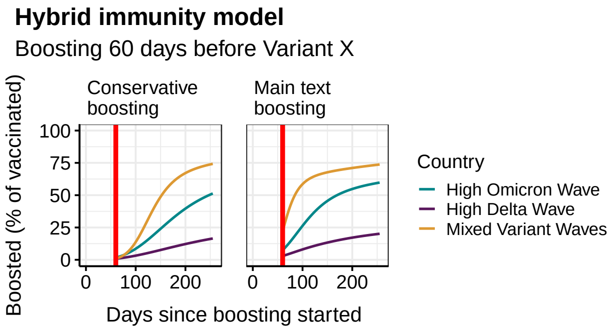

Figure S19: HIM booster curves. Percentage of vaccinated adults boosted over time, under conservative or main-text booster rollout, if boosting starts 60 days prior to Variant X. Red lines denote the start of Variant X.

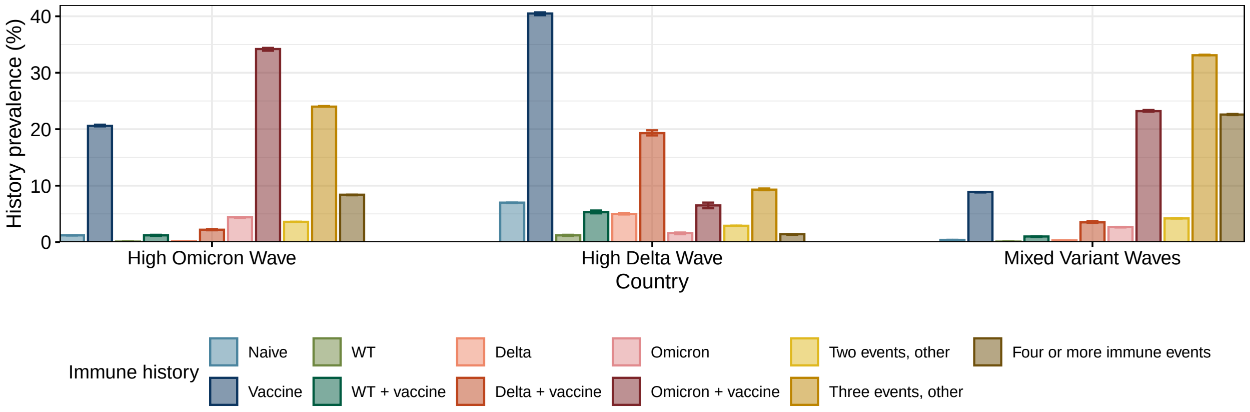

Figure S20: Distribution of immune histories by setting in the HSM on day 899, under a no booster scenario with 500 replicates - used to estimate cross protection and number of prior infections in the HIM (Supplementary Table S9). 95% confidence intervals from t-distribution are shown (whiskers).

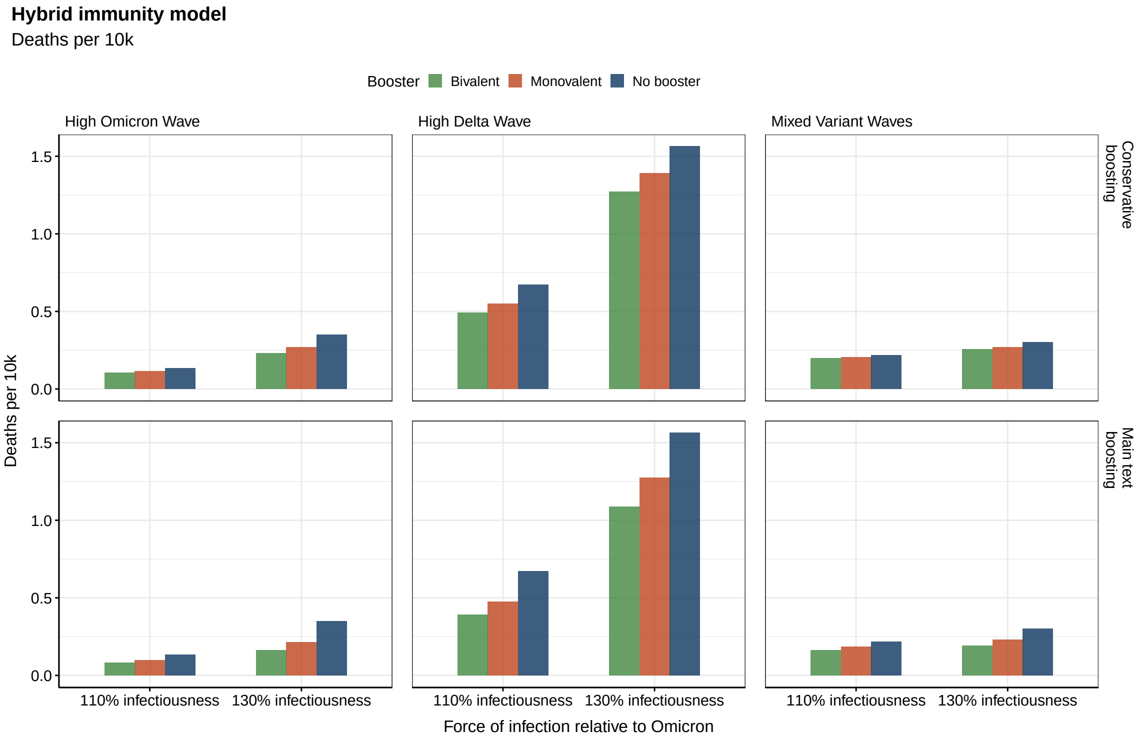

Figure S21: Total deaths per 10,000 in the HIM, during Variant X, if booster rollout is conservative or follows main-text speed. The HIM model structure is shown in Supplementary Fig. S2. General parameters are shown in Supplementary Table S1, stringency in Supplementary Table S4, initial conditions in Supplementary Table S10, cross-protection in Supplementary Table S9, and boosting parameters in Supplementary Table S8. In the conservative booster scenario, booster rollout speed WB_h matches the timing of primary series vaccination (Supplementary Table S5).

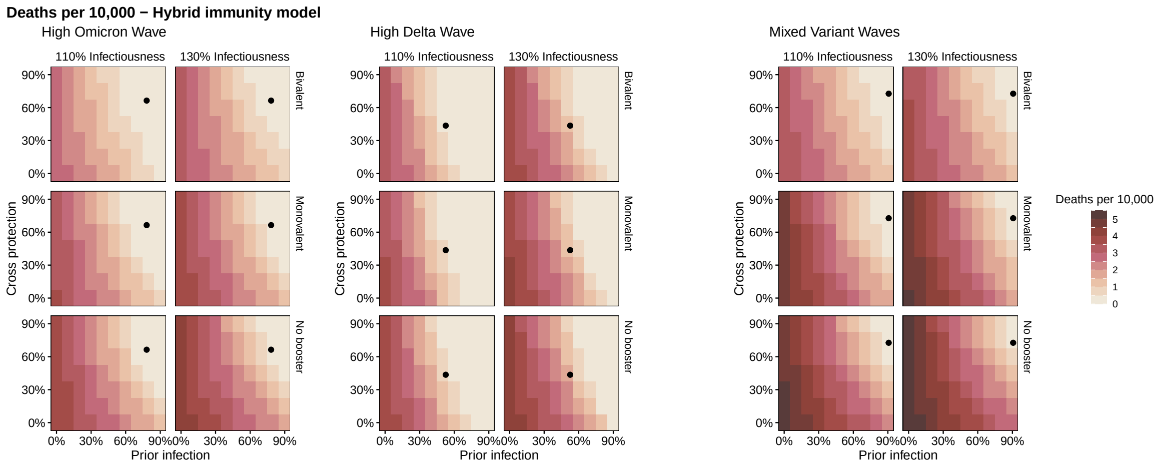

Figure S22: Total deaths per 10,000 in the HIM, during Variant X, corresponding to main text scenarios. The HIM model structure is shown in Supplementary Fig. S2. General parameters are shown in Supplementary Table S1, stringency in Supplementary Table S4, initial conditions in Supplementary Table S10, cross-protection in Supplementary Table S9, and boosting parameters in Supplementary Table S8.

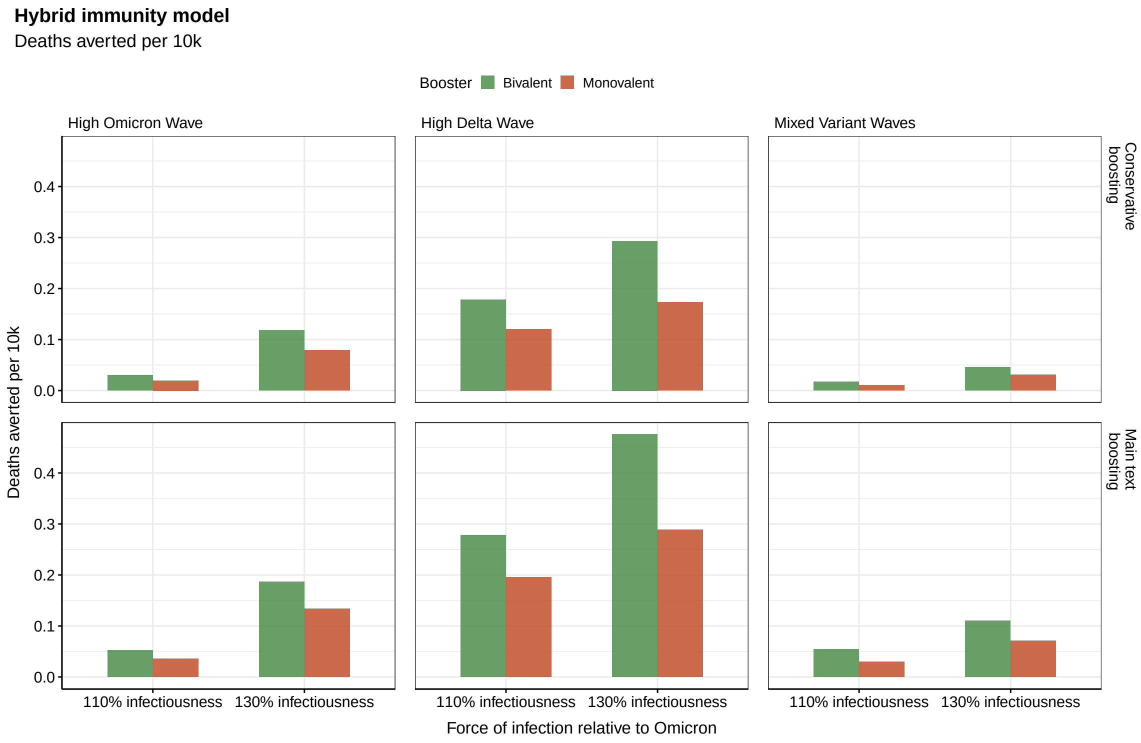

Figure S23: Deaths averted per 10,000 in the HIM during Variant X, under conservative or main-text booster rollout, if boosting starts 60 days prior to Variant X. The HIM model structure is shown in Supplementary Fig. S2. General parameters are shown in Supplementary Table S1, stringency in Supplementary Table S4, initial conditions in Supplementary Table S10, cross-protection in Supplementary Table S9, and boosting parameters in Supplementary Table S8. In the conservative booster scenario, booster rollout speed WB_h matches the timing of primary series vaccination (Supplementary Table S5).

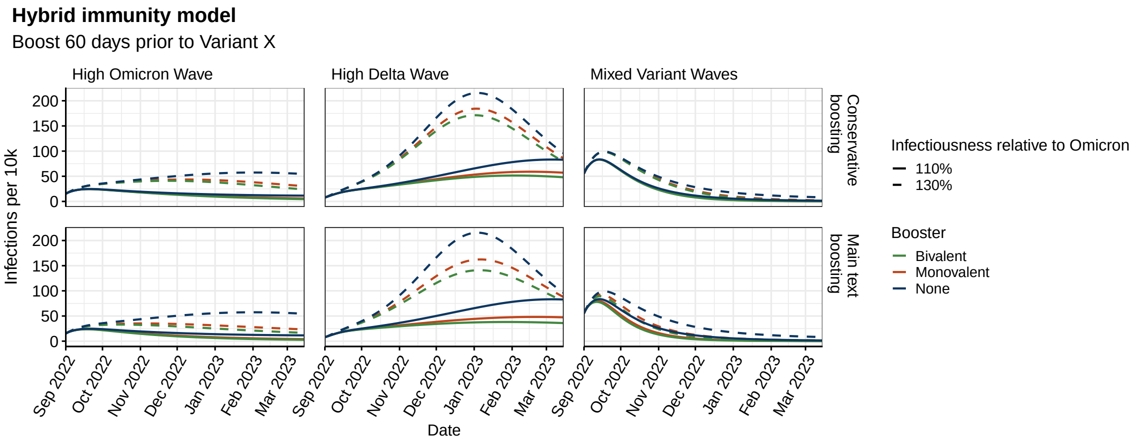

Figure S24: Cases per 10,000 in the HIM during Variant X under conservative or main-text booster rollout, if boosting starts 60 days prior to Variant X. The HIM model structure is shown in Supplementary Fig. S2. General parameters are shown in Supplementary Table S1, stringency in Supplementary Table S4, initial conditions in Supplementary Table S10, cross-protection in Supplementary Table S9, and boosting parameters in Supplementary Table S8. In the conservative booster scenario, booster rollout speed WB_h matches the timing of primary series vaccination (Supplementary Table S5).

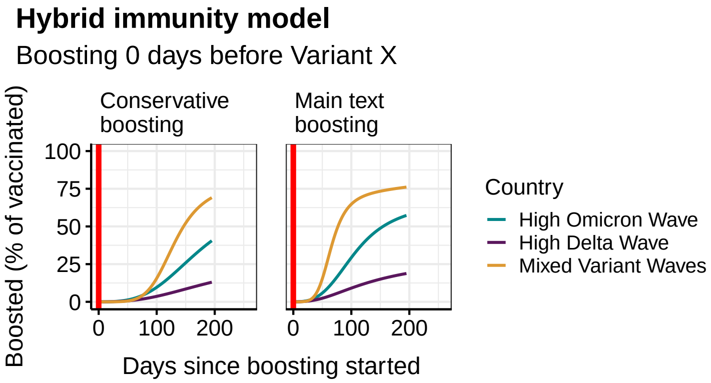

Figure S25: HIM booster curves. Percentage of vaccinated adults boosted over time, under conservative or main-text booster rollout, if boosting starts at the same time as Variant X. Red lines denote the start of the Variant X wave.

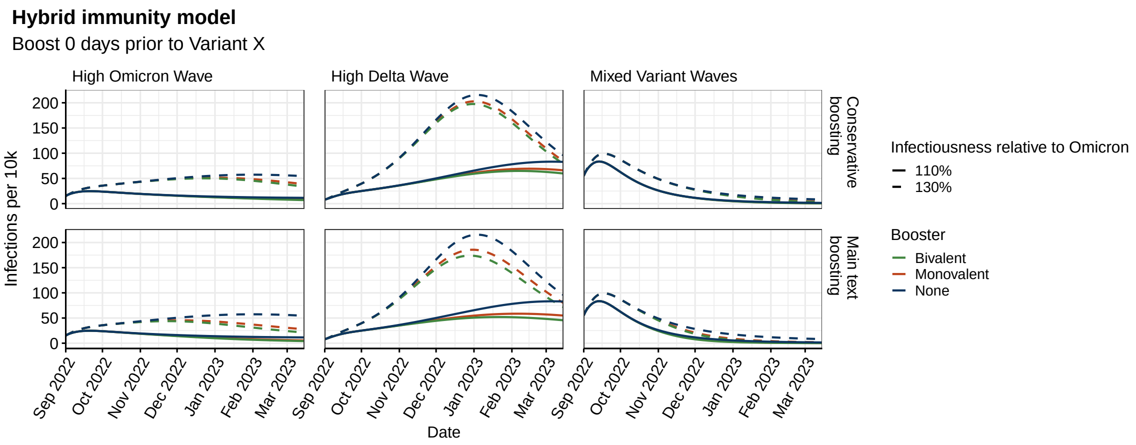

Figure S26: Cases per 10,000 in the HIM during the Variant X wave, under conservative or main-text booster rollout, if boosting starts at the same time as Variant X. The HIM model structure is shown in Supplementary Fig. S2. General parameters are shown in Supplementary Table S1, stringency in Supplementary Table S4, initial conditions in Supplementary Table S10, cross-protection in Supplementary Table S9, and boosting parameters in Supplementary Table S8. In the conservative booster scenario, booster rollout speed WB_h matches the timing of primary series vaccination (Supplementary Table S5).

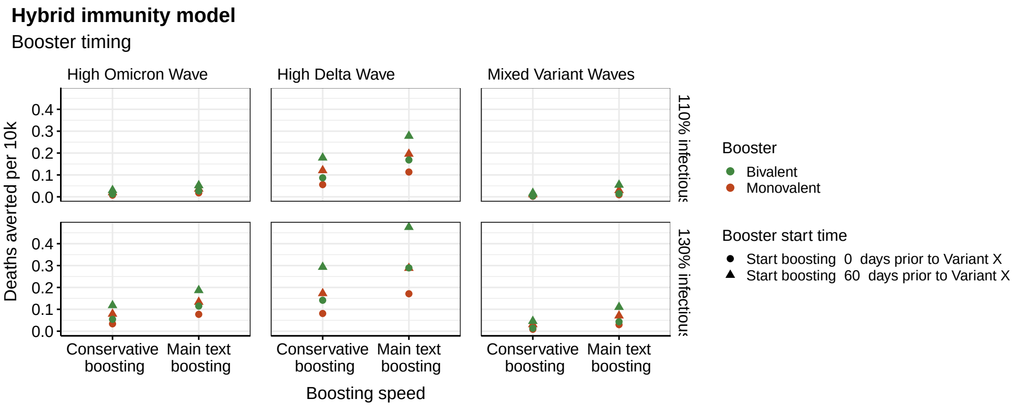

Figure S27: Deaths averted per 10,000 population in the HIM during Variant X, under conservative or main-text booster rollout. Boosting starts either at the start of Variant X (circles) or 60 days prior (triangles). The HIM model structure is shown in Supplementary Fig. S2. General parameters are shown in Supplementary Table S1, stringency in Supplementary Table S4, initial conditions in Supplementary Table S10, cross-protection in Supplementary Table S9, and boosting parameters in Supplementary Table S8. In the conservative booster scenario, booster rollout speed WB_h matches the timing of primary series vaccination (Supplementary Table S5).

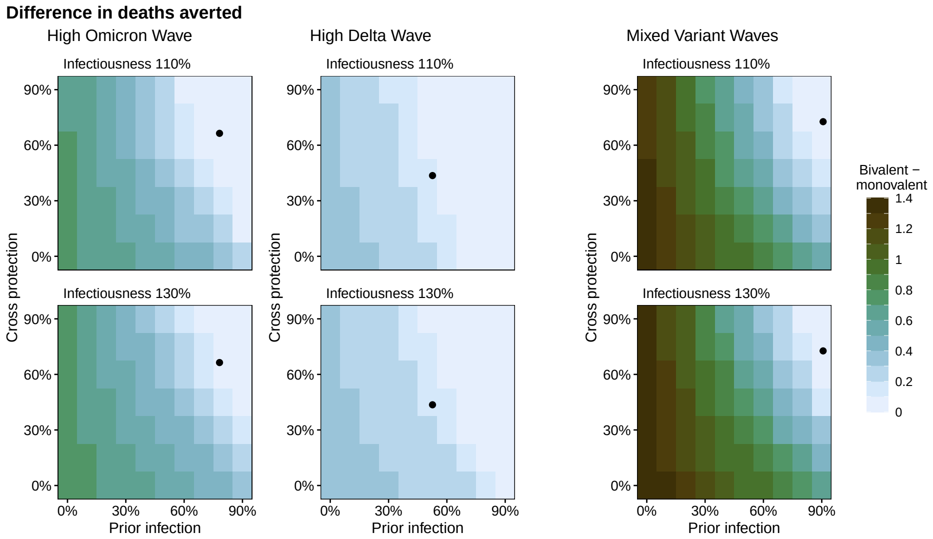

Figure S28: Difference in deaths averted per 10,000 between bivalent and monovalent boosters in the Hybrid-Immunity Model, corresponding to Figures 3 and 4.

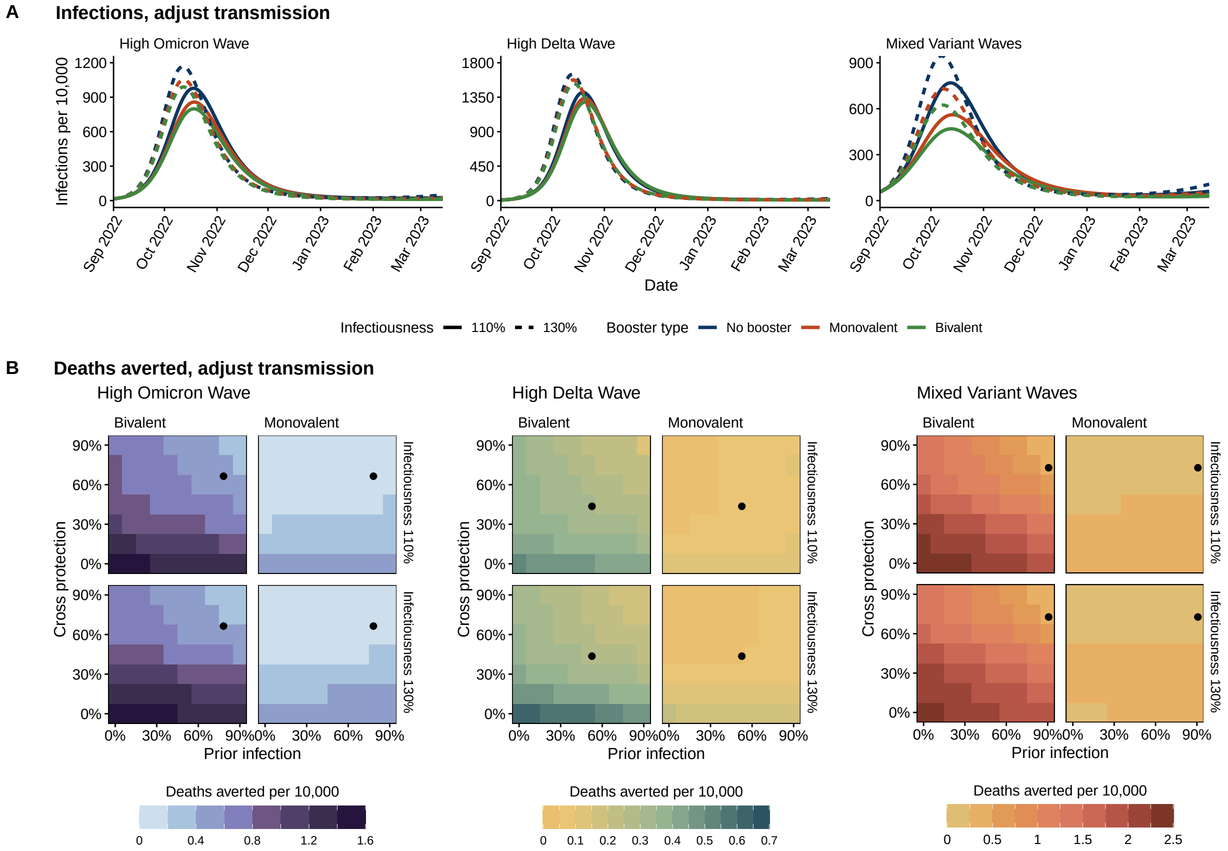

Figure S29: HIM projections, where the assumption that prior immunity reduces transmission, is lifted, but β is not recalibrated. (A) Infections per 10,000 during the Variant X period, where Variant X is considered to be 10% or 30% more infectious than Omicron. (B) Deaths averted per 10,000 under a bivalent or monovalent booster, compared to a "no boosting" scenario. Prior immunity represents the percentage of the population that has been previously infected. Cross protection represents the overlap between the population's immune history and the currently circulating variant. Immunity levels estimated by the HSM are included for each setting (black dots). The HIM model structure is shown in Supplementary Fig. S2. General parameters are shown in Supplementary Table S1, stringency in Supplementary Table S4, initial conditions in Supplementary Table S10, cross-protection in Supplementary Table S9, and boosting parameters in Supplementary Table S8.

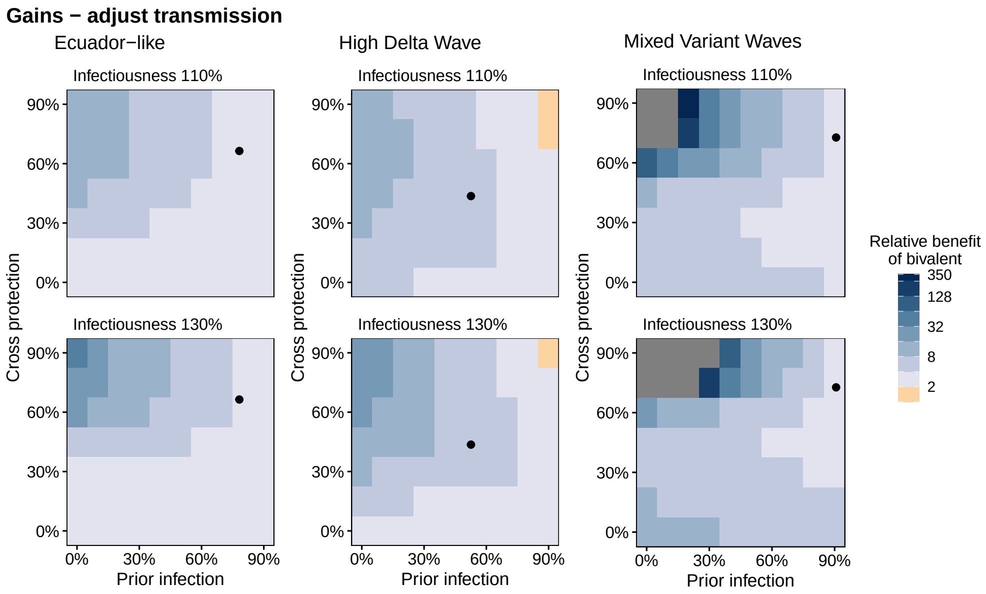

Figure S30: Comparison of bivalent vs. monovalent boosters in the HIM, where the assumption that prior immunity reduces transmission, is lifted, but β is not recalibrated. Relative benefit of the bivalent booster is calculated with Equation 1, matching the HSM. Grey values represent areas where zero deaths were averted by a monovalent booster and thus Equation 1 cannot be calculated. Prior immunity represents the percentage of the population that has been previously infected. Cross protection represents the overlap between the population's immune history and the currently circulating variant. Immunity levels estimated by the HSM are included for each setting (Supplementary Table S9; black dots). The HIM model structure is shown in Supplementary Fig. S2. General parameters are shown in Supplementary Table S1, stringency in Supplementary Table S4, initial conditions in Supplementary Table S10, cross-protection in Supplementary Table S9, and boosting parameters in Supplementary Table S8.

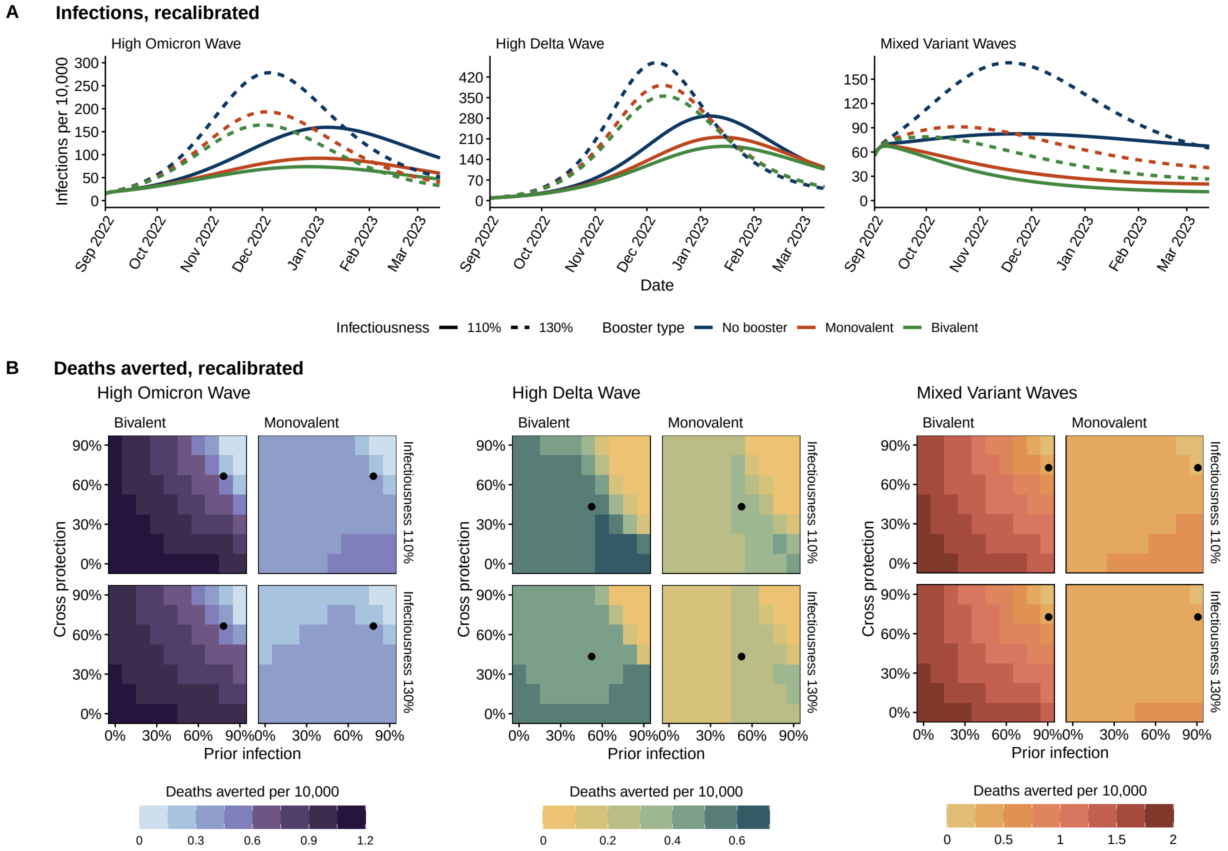

Figure S31: HIM projections, where the assumption that prior immunity reduces transmission, is lifted, and β is recalibrated. (A) Infections per 10,000 during the Variant X period, where Variant X is considered to be 10% or 30% more infectious than Omicron. (B) Deaths averted per 10,000 under a bivalent or monovalent booster, compared to a "no boosting" scenario. Prior immunity represents the percentage of the population that has been previously infected. Cross protection represents the overlap between the population's immune history and the currently circulating variant. Immunity levels estimated by the HSM are included for each setting (Supplementary Table S9; black dots). The HIM model structure is shown in Supplementary Fig. S2. General parameters are shown in Supplementary Table S1, stringency in Supplementary Table S4, initial conditions in Supplementary Table S10, cross-protection in Supplementary Table S9, and boosting parameters in Supplementary Table S8.

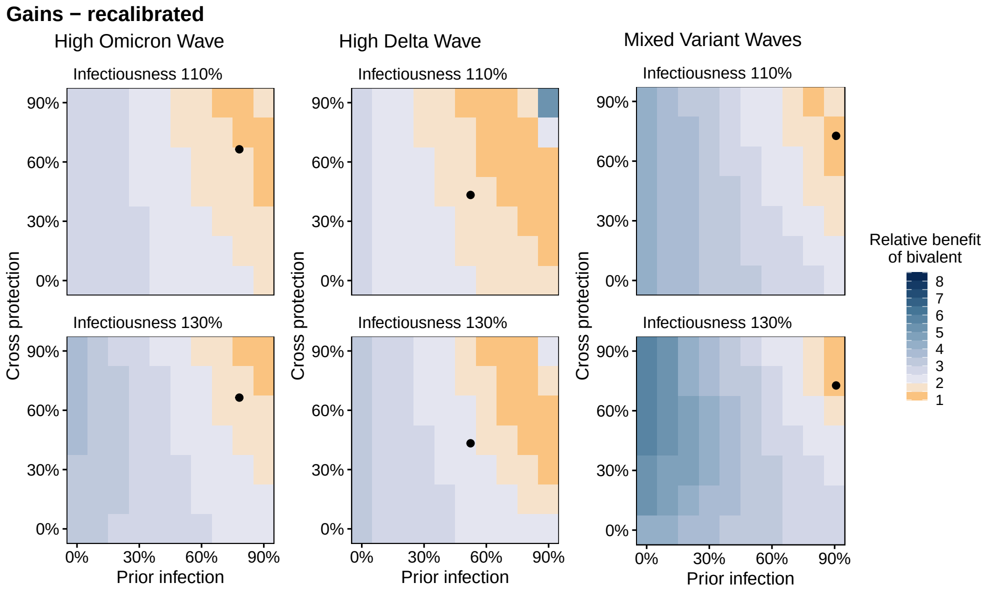

Figure S32: Comparison of bivalent vs. monovalent boosters in the HIM, where the assumption that prior immunity reduces transmission, is lifted, and β is recalibrated. Relative benefit of the bivalent booster is calculated with Equation 1, matching the HSM. Prior immunity represents the percentage of the population that has been previously infected. Cross protection represents the overlap between the population's immune history and the currently circulating variant. Immunity levels estimated by the HSM are included for each setting (Supplementary Table S9; black dots). The HIM model structure is shown in Supplementary Fig. S2. General parameters are shown in Supplementary Table S1, stringency in Supplementary Table S4, initial conditions in Supplementary Table S10, cross-protection in Supplementary Table S9, and boosting parameters in Supplementary Table S8.

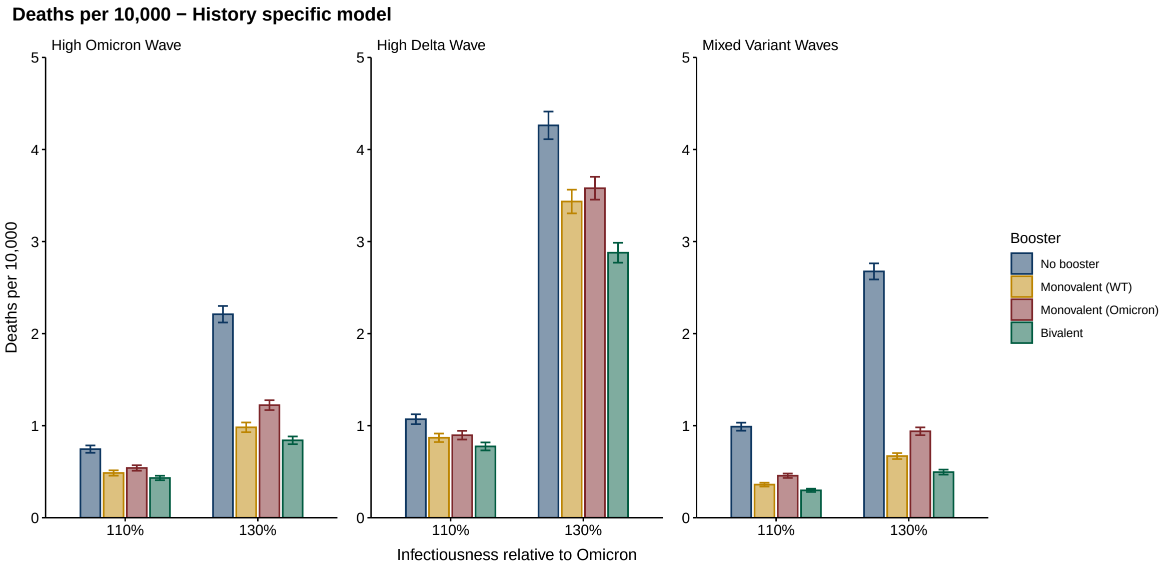

Figure S33: Deaths per 10,000 individuals in the HSM, with 500 replicates per scenario. 95% confidence intervals from t-distribution are shown (whiskers).

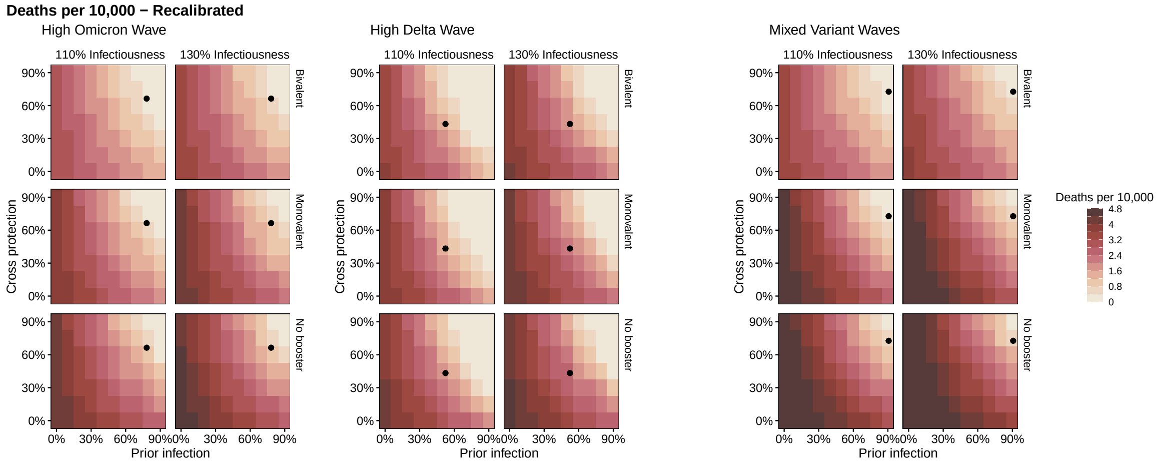

Figure S34: Total deaths per 10,000 in the HIM if the assumption that prior immunity reduces transmission is lifted and β is recalibrated. The HIM model structure is shown in Supplementary Fig. S2. General parameters are shown in Supplementary Table S1, stringency in Supplementary Table S4, initial conditions in Supplementary Table S10, cross-protection in Supplementary Table S9, and boosting parameters in Supplementary Table S8.

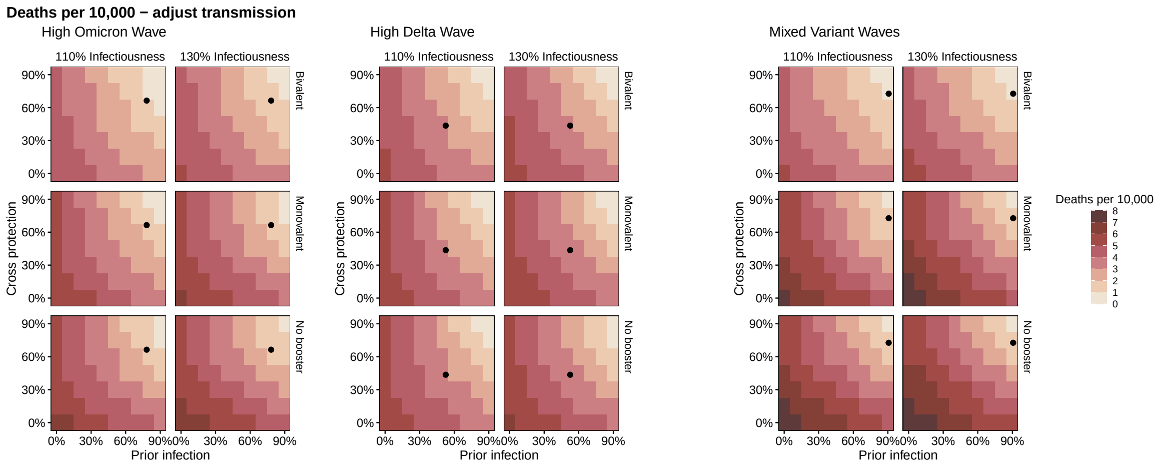

Figure S35: Total deaths per 10,000 in the HIM if the assumption that prior immunity reduces transmission is lifted but β is not recalibrated. The HIM model structure is shown in Supplementary Fig. S2. General parameters are shown in Supplementary Table S1, stringency in Supplementary Table S4, initial conditions in Supplementary Table S10, cross-protection in Supplementary Table S9, and boosting parameters in Supplementary Table S8.

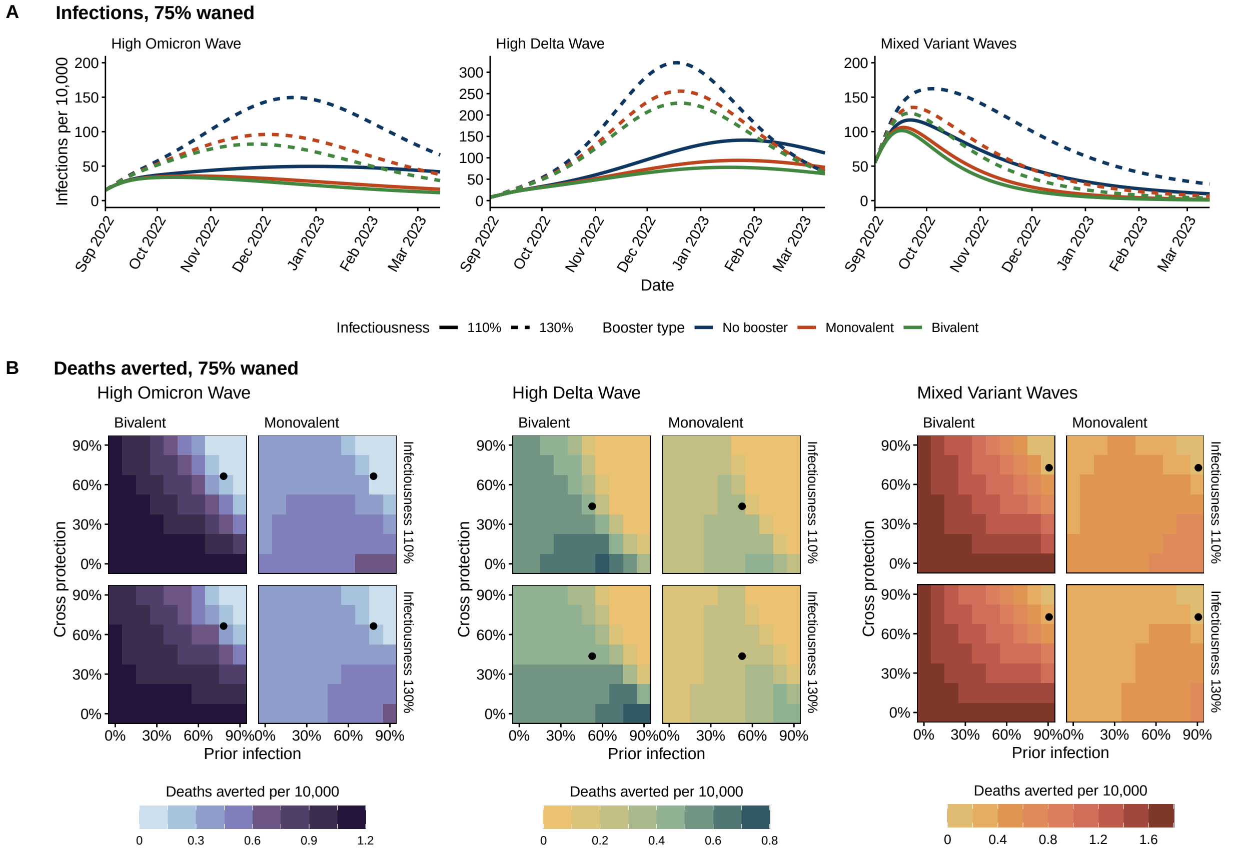

Figure S36: HIM projections, where 75% of previously infected individuals have waned at the start of simulations. (A) Infections per 10,000 during the Variant X period, where Variant X is considered to be 10% or 30% more infectious than Omicron. (B) Deaths averted per 10,000 under a bivalent or monovalent booster, compared to a ”no boosting” scenario. Prior immunity represents the percentage of the population that has been previously infected. Cross protection represents the overlap between the population's immune history and the currently circulating variant. Immunity levels estimated by the HSM are included for each setting (Supplementary Table S9; black dots). The HIM model structure is shown in Supplementary Fig. S2. General parameters are shown in Supplementary Table S1, stringency in Supplementary Table S4, initial conditions in Supplementary Table S10, cross-protection in Supplementary Table S9, and boosting parameters in Supplementary Table S8.

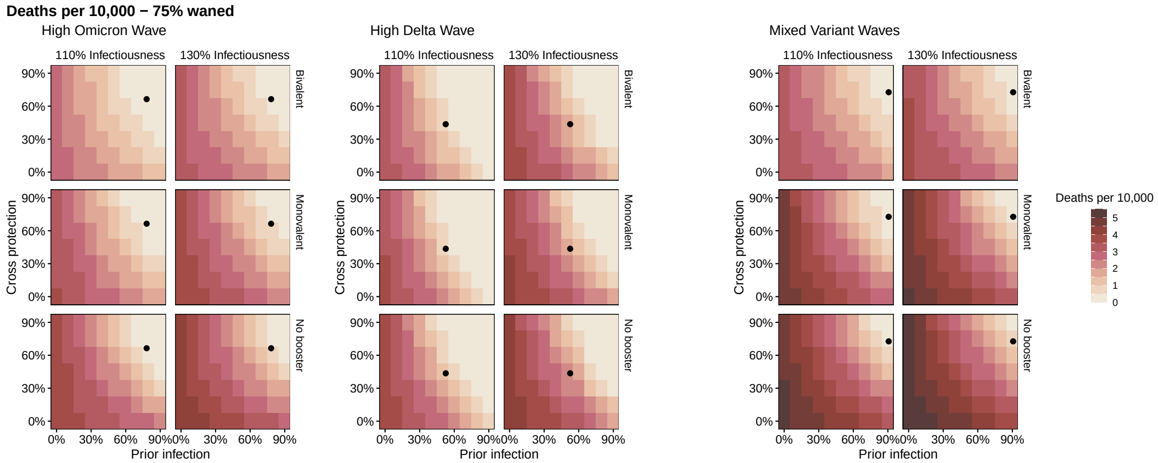

Figure S37: Total deaths per 10,000 in the HIM if 75% of previously infected individuals have waned. The HIM model structure is shown in Supplementary Fig. S2. General parameters are shown in Supplementary Table S1, stringency in Supplementary Table S4, initial conditions in Supplementary Table S10, cross-protection in Supplementary Table S9, and boosting parameters in Supplementary Table S8. In the conservative booster scenario, booster rollout speed WB_h matches the timing of primary series vaccination (Supplementary Table S5).

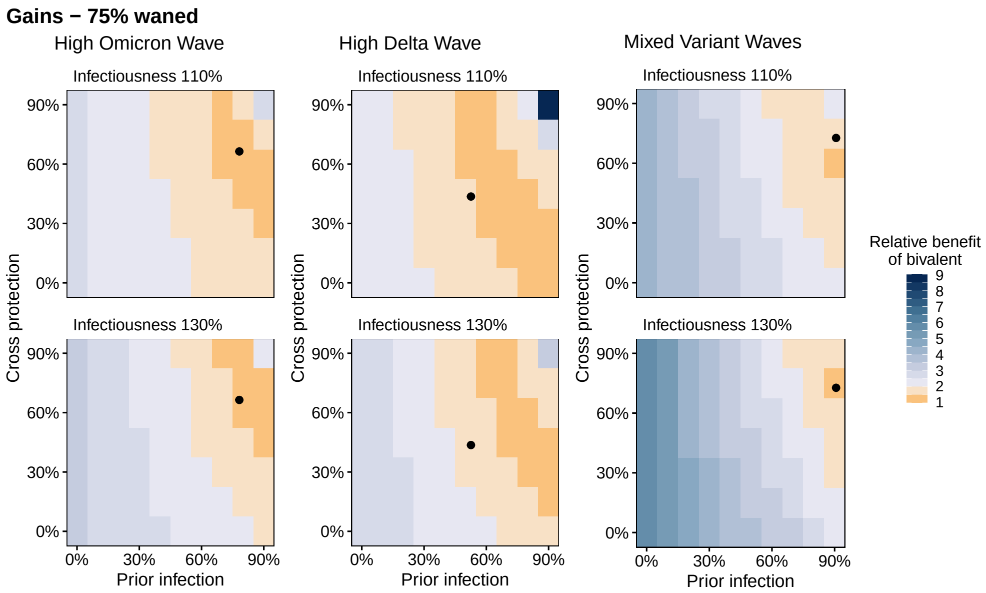

Figure S38: Comparison of bivalent vs. monovalent boosters in the HIM, where 75% of previously infected individuals have waned at the start of simulations. Relative benefit of the bivalent booster is calculated with Equation 1, matching the HSM. Prior immunity represents the percentage of the population that has been previously infected. Cross protection represents the overlap between the population's immune history and the currently circulating variant. Immunity levels estimated by the HSM are included for each setting (Supplementary Table S9; black dots). The HIM model structure is shown in Supplementary Fig. S2. General parameters are shown in Supplementary Table S1, stringency in Supplementary Table S4, initial conditions in Supplementary Table S10, cross-protection in Supplementary Table S9, and boosting parameters in Supplementary Table S8.

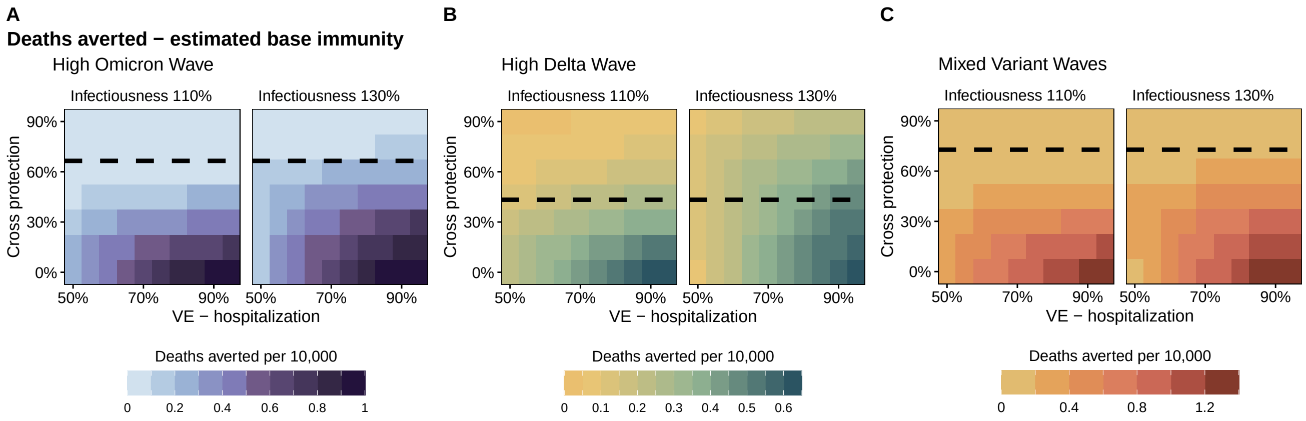

Figure S39: HIM projections, where the number of prior infections is fixed to the setting-specific estimate and cross protection and booster efficacy are varied. Cross protection represents the overlap between the population's immune history and the currently circulating variant. Setting-specific cross-protection estimates are included (black lines). The HIM model structure is shown in Supplementary Fig. S2. General parameters are shown in Supplementary Table S1, stringency in Supplementary Table S4, initial conditions in Supplementary Table S10, cross-protection in Supplementary Table S9, and boosting parameters in Supplementary Table S8.

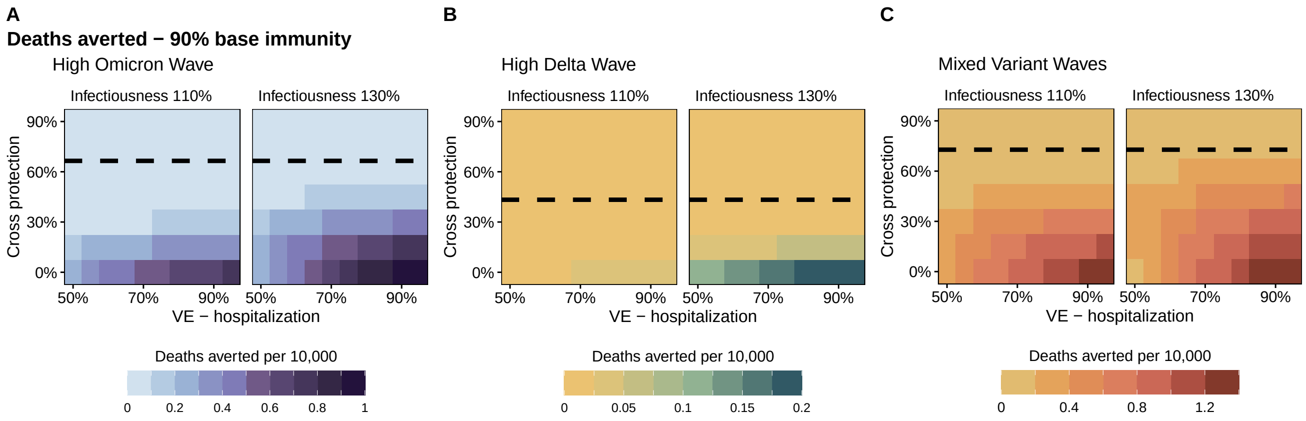

Figure S40: HIM projections, where the number of prior infections is set at 90% and cross protection and booster efficacy are varied. Cross protection represents the overlap between the population's immune history and the currently circulating variant. Setting-specific cross-protection estimates are included (black lines). The HIM model structure is shown in Supplementary Fig. S2. General parameters are shown in Supplementary Table S1, stringency in Supplementary Table S4, initial conditions in Supplementary Table S10, cross-protection in Supplementary Table S9, and boosting parameters in Supplementary Table S8.

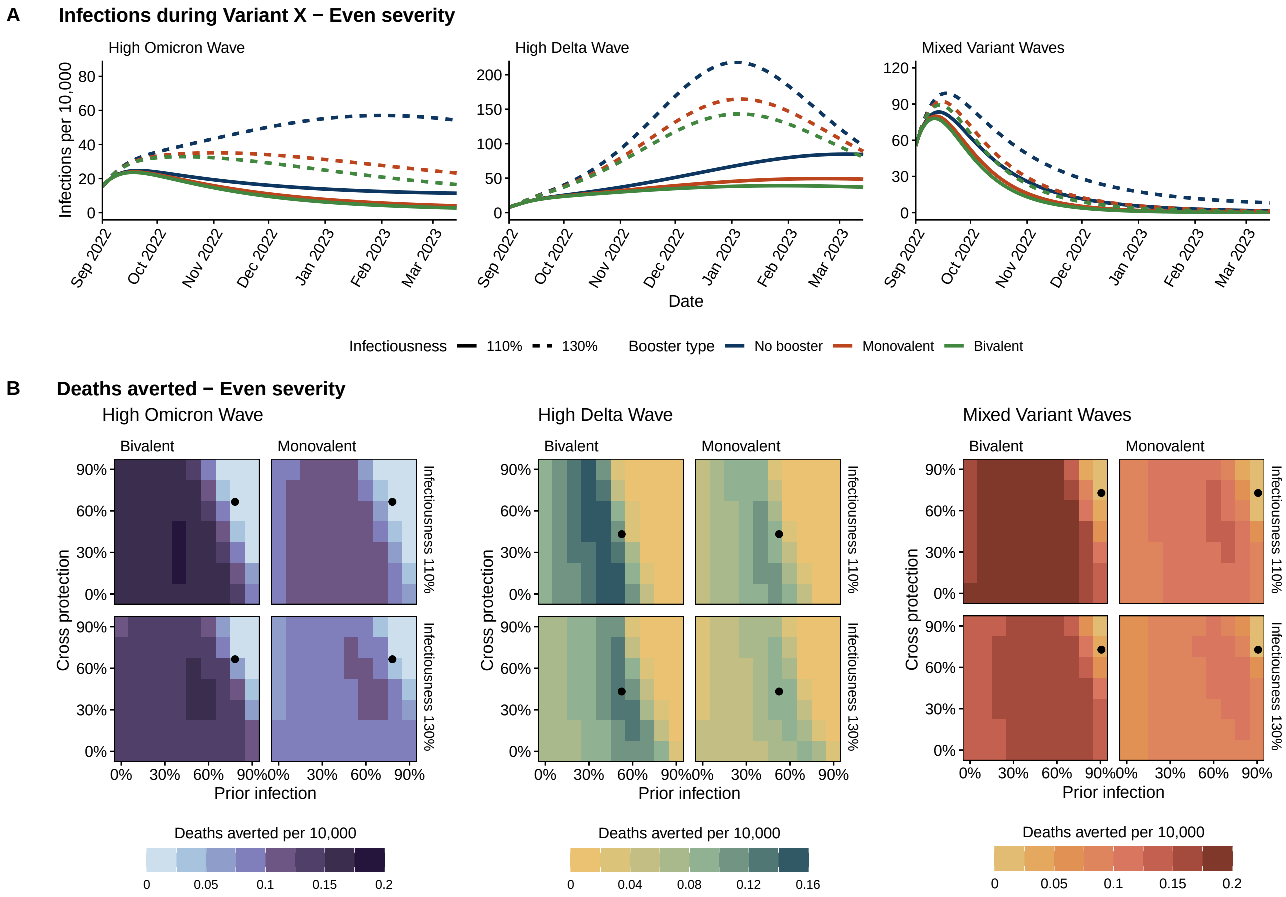

Figure S41: HIM booster impacts under the assumption that protection against severe disease is uniformly 95% after a recent exposure or vaccination, and wanes at the same rate for all individuals. This represents a scenario where protection against infection is driven by neutralizing antibody responses which differ in quality across immune histories and are sensitive to immune escape, but protection against severe disease is driven by durable T and B cell responses that are less sensitive to the changing variant landscape. (A) Infections per 10,000 during the Variant X period, where Variant X is considered to be 10% or 30% more infectious than Omicron. (B) Deaths averted per 10,000 under a bivalent or monovalent booster, compared to a 'no boosting' scenario. Prior immunity represents the percentage of the population that has been previously infected. Cross protection represents the overlap between the population's immune history and the currently circulating variant. Immunity levels estimated by the HSM are included for each setting (Supplementary Table S9; black dots). The HIM model structure is shown in Supplementary Fig. S2. General parameters are shown in Supplementary Table S1, stringency in Supplementary Table S4, initial conditions in Supplementary Table S10, cross-protection in Supplementary Table S9, and boosting parameters in Supplementary Table S8.

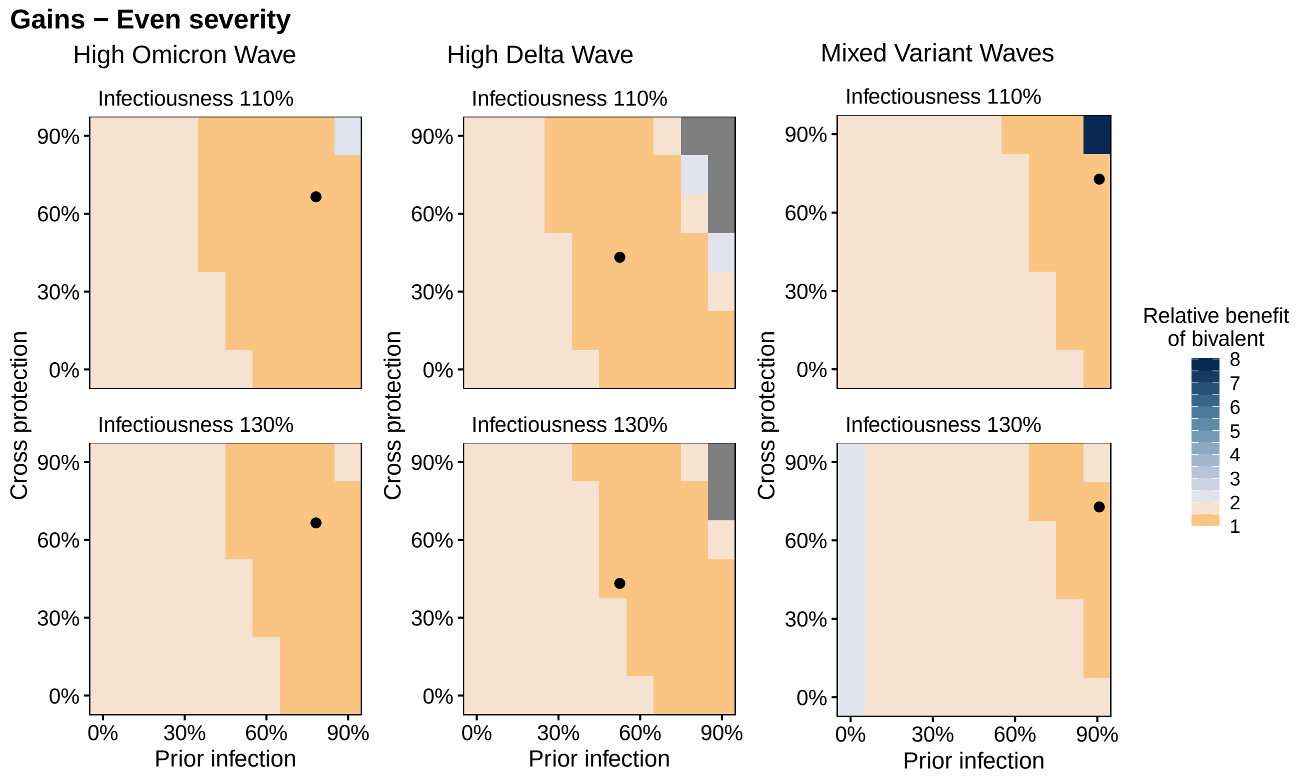

Figure S42: Comparison of bivalent vs. monovalent boosters in the HIM, under the assumption that protection against severe disease is uniformly 95% after a recent exposure or vaccination, and wanes at the same rate for all individuals. This represents a scenario where protection against infection is driven by neutralizing antibody responses which differ in quality across immune histories and are sensitive to immune escape, but protection against severe disease is driven by durable T and B cell responses that are less sensitive to the changing variant landscape. Relative benefit of the bivalent booster is calculated with Equation **??**, matching the HSM. Grey values represent high-immunity areas where zero deaths were averted by a monovalent booster and thus Equation **??** cannot be calculated. Prior immunity represents the percentage of the population that has been previously infected. Cross protection represents the overlap between the population's immune history and the currently circulating variant. Immunity levels estimated by the HSM are included for each setting (Supplementary Table S9; black dots).

S3 Tables

Parameter	Symbol	Value	Reference
Probability of infection given contact			
Wild type	β	18.9%	[3]
Delta	β	29.7%	[3]
Omicron	β	42.7%	[3]
Variant X	β	46.97, 55.5%	
Scale infectiousness for secondary infections	si	0.27	[17]
Incubation period	$\frac{1}{\epsilon}$	4 days	[18]
Fraction symptomatic	ν	0.4	[19–21]
Infectious Period	$\frac{1}{\gamma}$	7 days	[22]
Natural immunity[1]	$\frac{1}{\omega}$	10 months	[23, 24]
Case fatality rate			[6, 20]
Children	ρ_{ch}	High SES: 0.00044	
	ρ_{cl}	Low SES: 0.001364	
Adults	ρ_{ah}	High SES: 0.00356	
	ρ_{al}	Low SES: 0.00854	
Older adults	ρ_{eh}	High SES: 0.0346	
	ρ_{el}	Low SES: 0.0346	
HSM-specific			
Scale down protection after waning of immunity			
1 immune event	$d_{\omega,1}$	0.4	
2 immune events	$d_{\omega,2}$	0.7	
3+ immune events	$d_{\omega,3}$	0.85	
Protection against infection relative to severe disease	ϕ	80%	
HIM-specific			
Scale infection probability to match HSM force of infection			
Low SES	β_L	9.34%	
High SES	β_H	5.34%	
Vaccine effectiveness			
Two doses, no booster			[25–27]
Infection effectiveness	VE_i	0	
Severe disease effectiveness	VE_h	0.7	
Two doses, with monovalent booster			[25–28]
Infection effectiveness	VE_i	0.5256	
Severe disease effectiveness	VE_h	0.657	
Two doses, with bivalent booster			[25–28]
Infection effectiveness	VE_i	0.74	
Severe disease effectiveness	VE_h	0.925	
Probability of infection given contact (si sensitivity analysis)			[11]
Low SES	β_L	4.79%	
High SES	β_H	1.82%	

[1] HSM model used same duration of immunity for vaccine and booster-derived immunity.

Table S1: Parameter values used in HSM and HIM simulations. The case fatality rate is reduced by 50% during the Omicron and Variant X waves.

Age	'Mixed Variant Waves'	'High Omicron Wave'	'High Delta Wave'
Child	21,450	23,632	24,046
Adult	66,772	63,254	64,678
Old	11,774	13,112	11,272
Total	99,996	99,998	99,996

Table S2: HSM simulation sizes by setting.

Contact type	‘Mixed Variant Waves’	‘High Omicron Wave’	‘High Delta Wave’
Low to Low	1.53	1.55	2.89
Low to High	1.02	1.03	1.92
High to Low	0.58	0.59	1.10
High to High	0.87	0.89	1.65
Total contact	4.00	4.06	7.56

Table S3: HSM baseline contact rates across settings and SES, prior to application of stringency index.

Wave	‘Mixed Variant Waves’	‘High Omicron Wave’	‘High Delta Wave’
WT	0.42	0.40	0.22
Delta	0.51	0.35	0.24
Omicron	0.93	0.71	0.29
Variant X	0.93	0.71	0.29

Table S4: Wave-specific stringency across countries. These values were estimated in order to match relative wave sizes in the HSM to observed data (Supplementary Fig. S1).

Parameter	Meaning	'Mixed Variant Waves'	'High Omicron Wave'	'High Delta Wave'
k_L	Shape of vaccination trends, low SES	5.0	3.9	2.5
k_H	Shape of vaccination trends, high SES	5.5	3.1	2.8
V_{m_L}	Maximum vaccinated, low SES	0.72	0.79	0.78
V_{m_H}	Maximum vaccinated, high SES	0.97	1	0.93
W_{h_L}	Halfway week, low SES	21.3	30.6	32.1
W_{h_H}	Halfway week, high SES	19.9	24.5	23.4

Table S5: Setting- and SES-specific vaccination trends; parameters correspond to a Type-III functional response as detailed in [7] and shown in Supplementary Fig. S5.

Infectiousness	Setting	Scenario	Booster	Relative Benefit
110%	'High Omicron Wave'	History dependent	Bivalent	1.19
		History dependent	Monovalent (Omicron)	0.77
	'High Delta Wave'	History dependent	Bivalent	1.5
		History dependent	Monovalent (Omicron)	0.85
	'Mixed Variant Waves'	History dependent	Bivalent	1.1
		History dependent	Monovalent (Omicron)	0.84
130%	'High Omicron Wave'	History dependent	Bivalent	1.11
		History dependent	Monovalent (Omicron)	0.8
	'High Delta Wave'	History dependent	Bivalent	1.66
		History dependent	Monovalent (Omicron)	0.82
	'Mixed Variant Waves'	History dependent	Bivalent	1.08
		History dependent	Monovalent (Omicron)	0.87

Table S6: Relative benefit (Equation 1) of bivalent vs. WT monovalent booster formulation for HSM main text scenarios.

Setting	Analysis	Booster	Relative Benefit
'High Omicron Wave'	Boost like Malaysia	Bivalent	1.08
	Boost like Malaysia	Monovalent (Omicron)	0.83
	Conservative vaccine rollout	Bivalent	1.12
	Conservative vaccine rollout	Monovalent (Omicron)	0.69
	Same efficacy scenario	Bivalent	1.27
	Same efficacy scenario	Monovalent (Omicron)	1.03
	Same endpoint scenario	Bivalent	1.12
	Same endpoint scenario	Monovalent (Omicron)	0.97
	Vaccinate through Variant X	Bivalent	1.12
	Vaccinate through Variant X	Monovalent (Omicron)	0.75
	Even severity	Bivalent	1.08
	Even severity	Monovalent (Omicron)	0.88
	Poor efficacy	Bivalent	1.15
	Poor efficacy	Monovalent (Omicron)	0.83
	Monovalent attenuated to 53%	Bivalent	1.11
	Monovalent attenuated to 53%	Monovalent (Omicron)	0.74
	Monovalent not attenuated	Bivalent	0.94
	Monovalent not attenuated	Monovalent (Omicron)	0.83
'High Delta Wave'	Boost like Malaysia	Bivalent	1.37
	Boost like Malaysia	Monovalent (Omicron)	1.01
	Conservative vaccine rollout	Bivalent	1.62
	Conservative vaccine rollout	Monovalent (Omicron)	0.85
	Same efficacy scenario	Bivalent	2.06
	Same efficacy scenario	Monovalent (Omicron)	1.15
	Same endpoint scenario	Bivalent	1.26
	Same endpoint scenario	Monovalent (Omicron)	1.06
	Vaccinate through Variant X	Bivalent	1.80
	Vaccinate through Variant X	Monovalent (Omicron)	1.10
	Even severity	Bivalent	1.40
	Even severity	Monovalent (Omicron)	1.02
	Poor efficacy	Bivalent	1.79
	Poor efficacy	Monovalent (Omicron)	1.21
	Monovalent attenuated to 53%	Bivalent	1.45
	Monovalent attenuated to 53%	Monovalent (Omicron)	1.00
	Monovalent not attenuated	Bivalent	1.01
	Monovalent not attenuated	Monovalent (Omicron)	0.88
'Mixed Variant Waves'	Boost like Malaysia	Bivalent	1.09
	Boost like Malaysia	Monovalent (Omicron)	0.86
	Conservative vaccine rollout	Bivalent	1.08
	Conservative vaccine rollout	Monovalent (Omicron)	0.85
	Same efficacy scenario	Bivalent	1.17
	Same efficacy scenario	Monovalent (Omicron)	0.99
	Same endpoint scenario	Bivalent	1.09

	Same endpoint scenario	Monovalent (Omicron)	1.01
	Vaccinate through Variant X	Bivalent	1.08
	Vaccinate through Variant X	Monovalent (Omicron)	0.89
	Even severity	Bivalent	1.05
	Even severity	Monovalent (Omicron)	0.93
	Poor efficacy	Bivalent	1.09
	Poor efficacy	Monovalent (Omicron)	0.91
	Monovalent attenuated to 53%	Bivalent	1.04
	Monovalent attenuated to 53%	Monovalent (Omicron)	0.87
	Monovalent not attenuated	Bivalent	1.00
	Monovalent not attenuated	Monovalent (Omicron)	0.89

Table S7: Relative benefit (Equation 1) of bivalent vs. WT monovalent booster formulation for supplementary analyses in the HSM. All analyses assume that Variant X is 30% more infectious than Omicron.

Parameter	Meaning	'Mixed Variant Waves'	'High Omicron Wave'	'High Delta Wave'
kB_L	Shape of booster trends, low SES	5.0	3.9	2.5
kB_H	Shape of booster trends, high SES	5.5	3.1	2.8
B_{m_L}	Maximum boosted (excluding children), low SES	0.91	0.87	0.35
B_{m_H}	Maximum boosted (excluding children), high SES	0.90	0.88	0.35
WB_{h_L}	Halfway week, low SES	11.3	20.6	22.1
WB_{h_H}	Halfway week, high SES	9.9	14.5	13.4

Table S8: Setting- and SES-specific booster trends.

Setting	Prior infection	Cross protection
‘High Omicron Wave’	78.2%	0.664
‘High Delta Wave’	52.6%	0.436
‘Mixed Variant Waves’	90.7%	0.727

Table S9: Setting-specific benchmarking parameters based on no booster states estimated from the HSM at the start of the simulation. Cross protection is calculated using the Variant X column of Figure 1A and prior infection is calculated based on final states shown in Figures 1B and S3.

Parameter	Setting			Reference
	'High Omicron Wave'	'High Delta Wave'	'Mixed Variant Waves'	
Age distribution of infection				[12–14]
Child	53.4%	35.4%	36.7%	
Adult	37.8%	58.7%	57.8%	
Older adults	8.8%	6.0%	5.5%	
Population age distribution				
Child	41.0%	35.5%	36.2%	
Adult	52.6%	58.7%	59.0%	
Older adults	6.4%	5.8%	4.8%	
Initial reported infections	1798	19153	11236	[8]
Reporting rate (symptomatic)	0.189	0.053	0.178	[12–14]

Table S10: Initial conditions used in Hybrid Immunity Model simulations by setting

Setting	SAR	Booster	HIM	HSM	Lower	Upper
'High Omicron Wave'	110%	Bivalent	0.05	0.31	0.27	0.36
		Monovalent (WT)	0.04	0.26	0.21	0.31
		Monovalent (Omicron)		0.2	0.15	0.26
	130%	Bivalent	0.19	1.37	1.27	1.47
		Monovalent (WT)	0.13	1.23	1.13	1.33
		Monovalent (Omicron)		0.99	0.88	1.09
'High Delta Wave'	110%	Bivalent	0.28	0.3	0.23	0.37
		Monovalent (WT)	0.2	0.2	0.13	0.28
		Monovalent (Omicron)		0.17	0.1	0.25
	130%	Bivalent	0.48	1.38	1.2	1.57
		Monovalent (WT)	0.29	0.83	0.63	1.02
		Monovalent (Omicron)		0.68	0.49	0.87
'Mixed Variant Waves'	110%	Bivalent	0.05	0.69	0.64	0.74
		Monovalent (WT)	0.03	0.63	0.58	0.68
		Monovalent (Omicron)		0.53	0.48	0.58
	130%	Bivalent	0.11	2.18	2.09	2.27
		Monovalent (WT)	0.07	2.01	1.91	2.1
		Monovalent (Omicron)		1.74	1.64	1.83

Table S11: Deaths averted per 10,000 in the HIM versus HSM.

Setting	SAR	Relative Benefit
‘High Omicron Wave’	110%	1.25
	130%	1.46
‘High Delta Wave’	110%	1.4
	130%	1.66
‘Mixed Variant Waves’	110%	1.67
	130%	1.57

Table S12: Setting-specific relative benefit in the HIM main text scenarios (black dots in Figure 4).

Setting	Analysis	Booster	Relative Benefit
'High Omicron Wave'	Delay 30 days	Bivalent	1.25
	Delay 30 days	Monovalent (Omicron)	0.83
	Delay 60 days	Bivalent	1.26
	Delay 60 days	Monovalent (Omicron)	0.82
	Delay 90 days	Bivalent	1.19
	Delay 90 days	Monovalent (Omicron)	0.74
'High Delta Wave'	Delay 30 days	Bivalent	1.69
	Delay 30 days	Monovalent (Omicron)	0.97
	Delay 60 days	Bivalent	1.53
	Delay 60 days	Monovalent (Omicron)	1.11
	Delay 90 days	Bivalent	2.36
	Delay 90 days	Monovalent (Omicron)	0.64
'Mixed Variant Waves'	Delay 30 days	Bivalent	1.08
	Delay 30 days	Monovalent (Omicron)	0.86
	Delay 60 days	Bivalent	1.10
	Delay 60 days	Monovalent (Omicron)	0.88
	Delay 90 days	Bivalent	1.06
	Delay 90 days	Monovalent (Omicron)	0.81

Table S13: Relative benefit (Equation 1) of bivalent vs. WT monovalent booster formulation for delayed booster rollout scenarios in the HSM. All analyses assume that Variant X is 30% more infectious than Omicron.

Setting	Bivalent Delay	Relative Benefit
‘High Omicron Wave’	30 days	0.89
	60 days	0.67
	90 days	0.41
‘High Delta Wave’	30 days	1.39
	60 days	0.87
	90 days	0.63
‘Mixed Variant Waves’	30 days	0.91
	60 days	0.69
	90 days	0.49

Table S14: Setting-specific relative benefit in the HSM if bivalent boosting is delayed 30, 60 or 90 days compared to immediate rollout of monovalent (WT) boosters.

References

[1] Population estimates and projections for 227 countries and areas. `https://www.census.gov/data-tools/demo/idb/#/pop?COUNTRY_YEAR=2022&COUNTRY_YR_ANIM=2022&FIPS_SINGLE=EC&FIPS=EC&popPages=BYAGE&POP_YEARS=2022&menu=popViz` (2022).

[2] Prem, K., Cook, A. R. & Jit, M. Projecting social contact matrices in 152 countries using contact surveys and demographic data. *PLOS Computational Biology* **13**, e1005697 (2017).

[3] Madewell, Z. J., Yang, Y., Longini, I. M., Halloran, M. E. & Dean, N. E. Household secondary attack rates of sars-cov-2 by variant and vaccination status: an updated systematic review and meta-analysis. *JAMA network open* **5**, e229317 (2022).

[4] Yechezkel, M. *et al.* Human mobility and poverty as key drivers of covid-19 transmission and control. *BMC public health* **21**, 1–13 (2021).

[5] Bokányi, E., Juhász, S., Karsai, M. & Lengyel, B. Universal patterns of long-distance commuting and social assortativity in cities. *Scientific reports* **11**, 1–10 (2021).

[6] Mena, G. E. *et al.* Socioeconomic status determines covid-19 incidence and related mortality in santiago, chile. *Science* **372**, eabg5298 (2021).

[7] Larsen, S. L. *et al.* Quantifying the impact of sars-cov-2 temporal vaccination trends and disparities on disease control. *Science Advances* **9**, eadh9920 (2023).

[8] Mathieu, E. *et al.* Coronavirus pandemic (covid-19). *Our World in Data* (2020). Https://ourworldindata.org/coronavirus.

[9] Lin, D.-Y. *et al.* Effectiveness of bivalent boosters against severe omicron infection. *New England Journal of Medicine* **388**, 764–766 (2023).

[10] Cao, Y., Gillespie, D. T. & Petzold, L. R. Avoiding negative populations in explicit poisson tau-leaping. *The Journal of chemical physics* **123**, 054104 (2005).

[11] Burki, T. K. Omicron variant and booster covid-19 vaccines. *Lancet Respiratory Medicine* **10** (2022).

[12] Murhekar, M. V. *et al.* Sars-cov-2 antibody seroprevalence in india, august–september, 2020: findings from the second nationwide household serosurvey. *Lancet Global Health* **59**, E257–E266 (2021).

[13] Megasari, N. L. A. *et al.* Seroepidemiological study of sars-cov-2 infection in east java, indonesia. *Plos one* **16**, e0251234 (2021).

[14] Acurio-Páez, D. *et al.* Seroprevalence of sars-cov-2 infection and adherence to preventive measures in cuenca, ecuador, october 2020, a cross-sectional study. *Int J Environ Res Public Health* **18**, 4657 (2021).

[15] Who coronavirus (covid-19) dashboard. `https://covid19.who.int/` (2022).

[16] Mateo-Urdiales, A. *et al.* Relative effectiveness of monovalent and bivalent mrna boosters in preventing severe covid-19 due to omicron ba. 5 infection up to 4 months post-administration in people aged 60 years or older in italy: a retrospective matched cohort study. *The Lancet Infectious Diseases* **23**, 1349–1359 (2023).

[17] Jung, J. *et al.* Transmission and infectious sars-cov-2 shedding kinetics in vaccinated and unvaccinated individuals. *JAMA Network Open* **5**, e2213606–e2213606 (2022).

[18] Wu, Y. *et al.* Incubation period of covid-19 caused by unique sars-cov-2 strains: A systematic review and meta-analysis. *JAMA Network Open* **5** (2022).

[19] Jung, C.-Y. *et al.* Clinical characteristics of asymptomatic patients with covid-19: a nationwide cohort study in south korea. *International Journal of Infectious Diseases* **99**, 266–268 (2020).

[20] CDC. COVID-19 Pandemic Planning Scenarios (2020). URL `https://www.cdc.gov/coronavirus/2019-ncov/hcp/planning-scenarios.html#box1`.

[21] Subramanian, R., He, Q. & Pascual, M. Quantifying asymptomatic infection and transmission of covid-19 in new york city using observed cases, serology, and testing capacity. *Proceedings of the National Academy of Sciences* **118**, e2019716118 (2021).

[22] Kissler, S. M., Tedijanto, C., Goldstein, E., Grad, Y. H. & Lipsitch, M. Projecting the transmission dynamics of sars-cov-2 through the postpandemic period. *Science* **368**, 860–868 (2020).

[23] Dan, J. M. *et al.* Immunological memory to sars-cov-2 assessed for up to 8 months after infection. *Science* **371** (2021).

[24] Altawalah, H. Antibody responses to natural sars-cov-2 infection or after covid-19 vaccination. *Vaccines (Basel)* **9** (2021).

[25] Andrews, N. *et al.* Covid-19 vaccine effectiveness against the omicron (b.1.1.529) variant. *New England Journal of Medicine* **386**, 1532–1546 (2022).

[26] Zeneca, A. Covid-19 vaccine astrazeneca real-world evidence summary (2021). URL `https://www.astrazeneca.com/content/dam/az/covid-19/media/factsheets/COVID-19_Vaccine_AstraZeneca_Real-World_Evidence_Summary.pdf`.

[27] Solante, R. *et al.* Expert review of global real-world data on covid-19 vaccine booster effectiveness & safety during the omicron-dominant phase of the pandemic. *Research Square* **22**, 1–16 (2022).

[28] Zeneca, A. Boosting with astrazeneca‘s vaccine provides high protection against omicron, equivalent to mrna covid-19 vaccines (2022). URL `https://www.astrazeneca.com/country-sites/thailand/press-releases/boosting-with-astrazenecas-vaccine-provides-high-protection-against-omicron-equivalent-to-mrna-covid-19-vaccines.html`.
